# Additive effect of wildfires on hospital admission in the Pantanal wetland, Brazil

**DOI:** 10.1038/s41598-025-13257-z

**Published:** 2025-07-29

**Authors:** André Calixto Gonçalves, Marcelo Marques de Magalhães, Gustavo Andrey de Almeida Lopes Fernandes, Ivan Filipe Fernandes, Kaylane de Almeida Faria, Gabriel Alexandre dos Santos, R. Valentim, Gabriela Moraes do Nascimento, Francisco Aparecido Rodrigues, Ricardo Ceneviva, Maria Clara Mendes Stama, Daniel Tetsuo G. Mori, Carolina Nascimento Capellini, Maria Eduarda Feres Garcia, Thiago Bruschi, Gabriel Poveda Gonçalves, Laís Costa Brito, Djeansy Djarny Etchiamiadzy Toussaint, Rejane Calixto Gonçalves, O.C. Luiz

**Affiliations:** 1 CECS-Center for Engineering, Modeling and Applied Social Sciences, Federal University of ABC, São Bernardo do Campo, Brazil; 2 School of Sciences and Engineering, São Paulo State University, Tupã, SP Brazil; 3 Department of Pharmaceutical Sciences, Federal University of São Paulo, Diadema, Brazil; 4 São Paulo Business School of the Getulio Vargas Foundation (FGV EAESP), São Paulo, Brazil; 5 Department of Physics, Federal University of São Paulo, Diadema, Brazil; 6 Institute of Mathematical and Computer Sciences (ICMC), University of São Paulo (USP), São Paulo, Brazil; 7 Department of Chemistry, Federal University of São Paulo, Diadema, Brazil; 8 Department of Environmental Sciences, Federal University of São Paulo, Diadema, Brazil; 9 Serviço Nacional de Aprendizagem Comercial-SENAC, São Paulo, Brazil; 10 Department of Chemistry Engineering, Federal University of São Paulo, Diadema, Brazil; 11 Department of Preventive Medicine, USP Medical School, São Paulo, Brazil

**Keywords:** Brazilian pantanal, Forest fires, Hospital admission, Respiratory diseases, Cardiovascular diseases, General linear model, Climate-change impacts, Environmental impact, Environmental impact, Health care, Public health, Epidemiology, Population screening

## Abstract

Forest fires release toxic pollutants from burning vegetation, posing serious risks to human health. The Brazilian Pantanal—the world’s largest continuous wetland—is currently experiencing severe wildfires. This situation may lead to a spike in hospital admissions for respiratory and cardiovascular conditions. To assess the health impacts of these fires on local populations, we applied a generalized linear model incorporating geographic variables, including air quality and climate data. This approach allowed us to evaluate the relationship between active fire outbreaks and hospitalizations among residents of the Pantanal. Our results reveal a troubling pattern: an increase of 10 active fires is associated with a significant rise in daily hospitalizations. Over a 10-year period, the risk of respiratory hospitalizations rose by 23.2%, while cardiovascular hospitalizations increased by 22.3% for every 10 additional fires. These findings underscore the urgent need for preventive action. By forecasting the potential daily rise in hospital admissions, our study offers valuable insights to help Brazilian authorities implement fire mitigation strategies and strengthen the preparedness of the local healthcare system.

**Supplementary Information:**

The online version contains supplementary material available at 10.1038/s41598-025-13257-z.

## Introduction

Brazil, one of the largest countries in the world, is home to vast and diverse vegetation. In 2022, approximately 495.8 million hectares—about 58.3% of the country’s land area—were covered by natural vegetation. This landscape includes several major biomes: the Amazon Rainforest, the Caatinga, the Atlantic Forest, the Cerrado, the Pampa, and the Pantanal^1,2^.

Preserving these forests plays a critical role in naturally mitigating carbon emissions. They help regulate the balance between carbon dioxide absorption and release into the atmosphere, a key factor in promoting global sustainability. In addition, forest conservation protects biodiversity and ensures the survival of countless plant and animal species native to these ecosystems^3–8^.

However, the expansion of large-scale economic activities has significantly threatened the conservation of Brazil’s remaining forests. As a result, the annual rate of land burned per square kilometer has risen sharply. Between 2003 and 2022, the total burned area reached nearly 6.6 million square kilometers, averaging around 327,000 square kilometers per year.

**Fig. 1:**
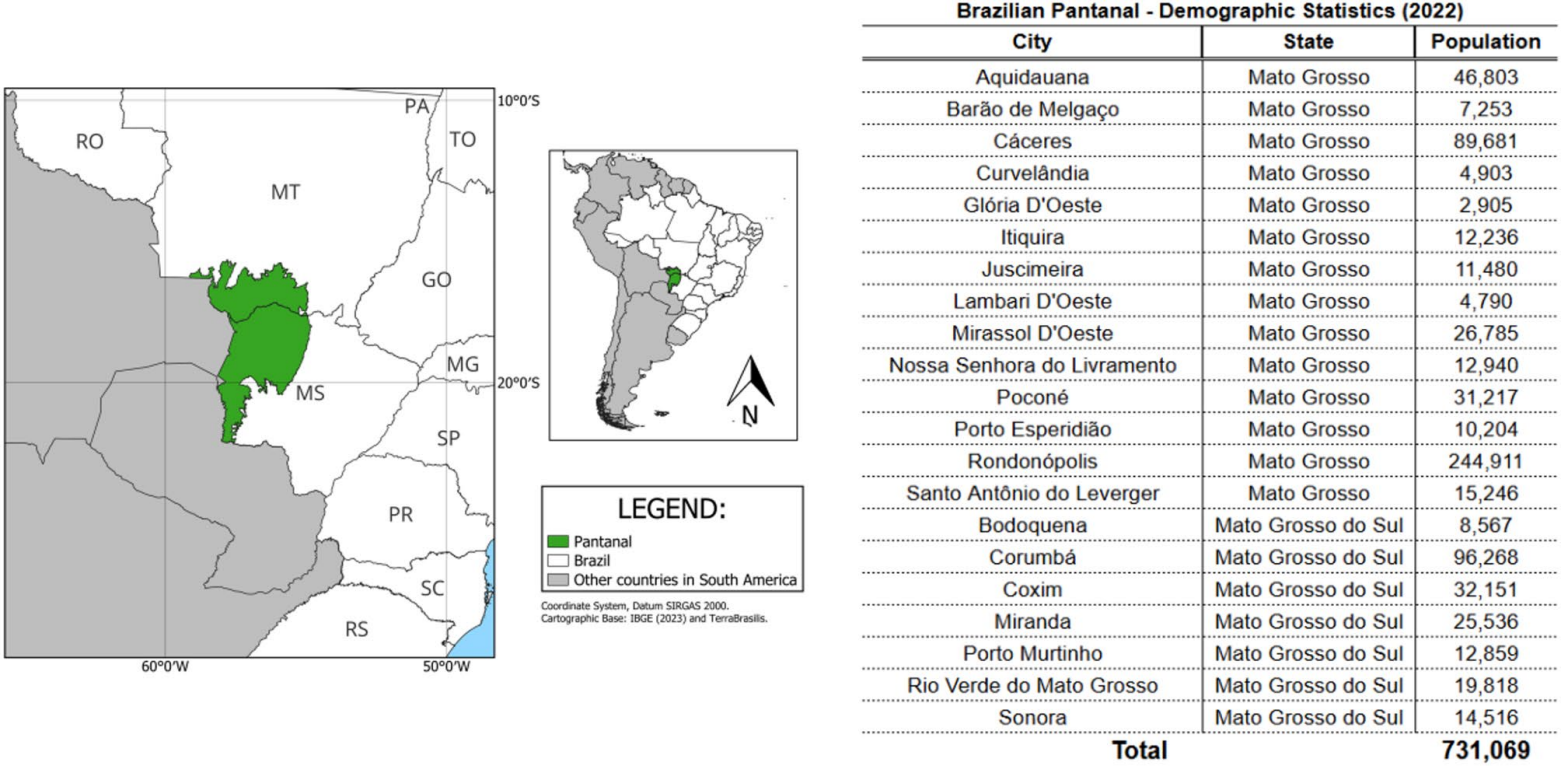
Geographic Boundaries and Municipal Population Data of the Brazilian Pantanal. Map showing the Pantanal biome area in Brazil. Data sources: IBGE SIDRA system (used under a CC BY 4.0 license). Map created by the authors using QGIS version 3.34.14 (https://qgis.org).

Among the biomes under considerable environmental pressure is the Pantanal, a unique wetland shared by Brazil, Bolivia, and Paraguay. In Brazil, the Pantanal covered approximately 150,000 square kilometers in 2022 and supported a population of about 731,000 people across 21 municipalities in the states of Mato Grosso and Mato Grosso do Sul, as shown in Fig. 1^9^.

Between 2019 and 2022, the average annual area burned per square kilometer in the Brazilian Pantanal increased by approximately 82% compared to the 2003–2018 period. Over the entire timeframe from 2003 to 2022, a total of 265,979 square kilometers burned—an average of 13,299 square kilometers per year^1,2,10^.

The Pantanal also has limited environmental protection, with only 4.6% of its territory designated as protected areas. Of this, just 2.9% falls under strict protection and 1.7% under sustainable use, placing the biome among the most environmentally vulnerable in Brazil. According to Law No. 9.985/2000, “strict protection” refers to the preservation of ecosystems in their natural state, allowing only indirect use of natural resources. In contrast, “sustainable use” permits responsible exploitation that ensures the long-term availability of renewable resources while maintaining biodiversity, ecological functions, and socioeconomic fairness^2,11^.

It is important to note that areas devastated by wildfires in the Pantanal are often converted into land for extensive cattle ranching and, to a lesser extent, for illegal agricultural expansion. In some cases, natural regeneration occurs. To illustrate the scale of land transformation, MapBiomas data show that between 1985 and 2022, agricultural land use in the region increased by 50%, despite the overall territory remaining the same. This suggests that much of this expansion has likely occurred in areas previously affected by fire^12–14^.

On the Brazilian side, the municipalities surrounding the Pantanal reported approximately 90 tons of plant extraction, 911,000 hectares of agricultural production, and 11.5 million head of livestock in 2022. Between 2003 and 2022, these figures grew by 25%, 94.77%, and 6.32%, respectively^10,15^.

**Fig. 2:**
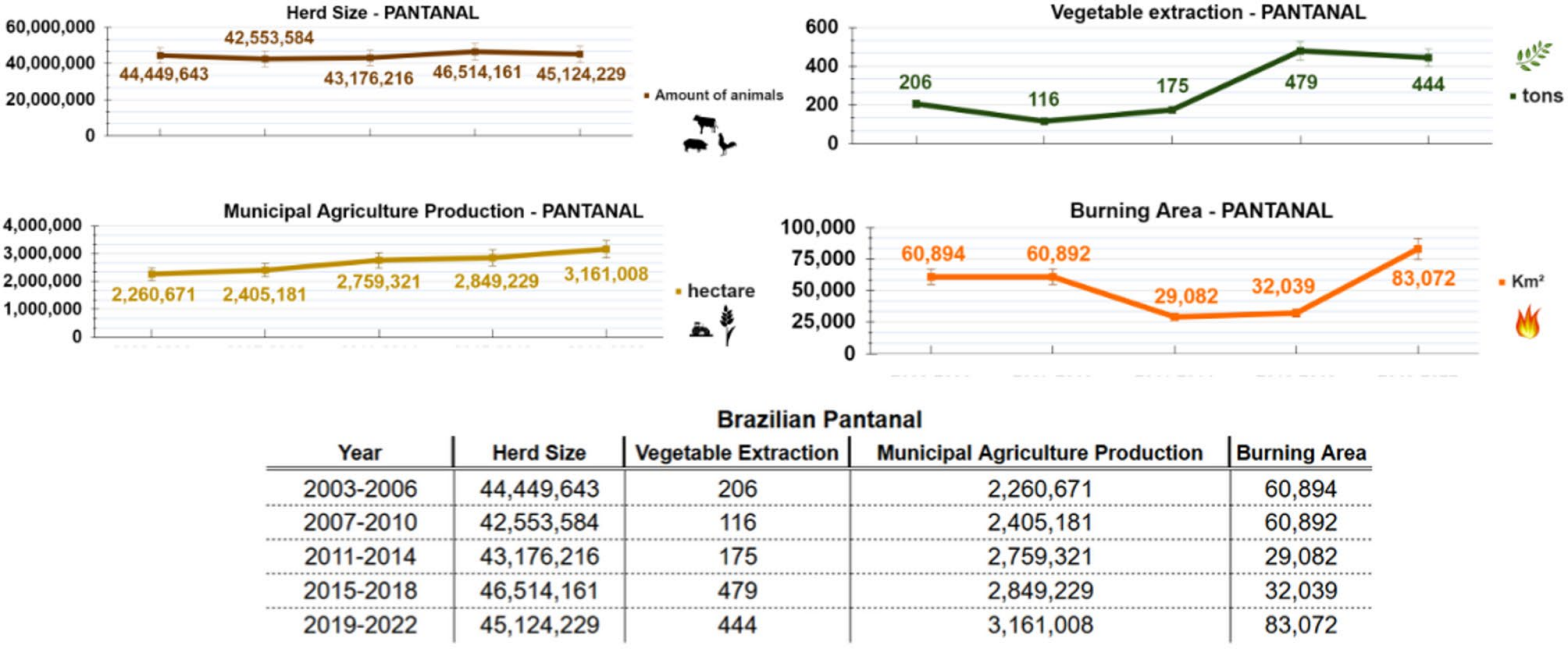
Trends in livestock, agricultural production, plant extraction, and burned area in the Brazilian Pantanal over the Past 20 Years. Data Source: IBGE Sidra System and INPE Burning System^16^.

Figure 2 presents data on municipal-level agricultural production, plant extraction, and livestock herd size in the Pantanal region, revealing consistent growth over a 20-year period. This trend suggests ongoing economic development in the area. Agricultural production includes data on planted and harvested areas, average crop yields, and the monetary value of production. Plant extraction encompasses annual totals of products such as food items, medicinal plants, waxes, fibers, and charcoal. The herd size graph reflects the number of livestock, including cattle, pigs, poultry, and other animals.

In contrast, the graph depicting burned areas shows considerable interannual variability. This fluctuation may be associated with the expansion of agricultural activity and pastureland, as suggested by existing literature.

Importantly, the period from 2019 to 2022 was marked by notable economic growth in the areas surrounding the Pantanal biome, coinciding with a relaxation of environmental protections. Prior to 2018, Brazil’s environmental legislation focused on stringent monitoring and enforcement. However, beginning in 2019, a series of governmental rollbacks weakened or dismantled key regulatory bodies, leading to reduced environmental oversight and a sharp increase in fire activity both within and around the biome (Fig. 2)^17–21^.

The consequences of this scenario extend beyond environmental degradation and climate change; they also pose serious risks to human health. Global studies have consistently shown that wildfire smoke has significant health impacts due to the release of harmful pollutants. The primary pollutants include fine and coarse particulate matter (PM_2.5_ and PM_10_), which are closely linked to respiratory and cardiovascular diseases^22–30^.

In addition to PM_2.5_ and PM_10_, wildfire smoke contains a complex mixture of pollutants. These include organic and black carbon, trace metals (such as potassium, magnesium, and zinc), soil residues, and ash. The gaseous components include carbon monoxide (CO), carbon dioxide (CO_2_), methane (CH_4_), nitrogen oxides (NO_x_), sulfur dioxide (SO_2_), and ozone (O_3_). Volatile organic compounds (VOCs)—including formaldehyde, benzene, and acrolein—are also present, along with secondary organic aerosols (SOAs) formed through atmospheric reactions^31–33^.

While the composition of emissions from burning vegetation is generally similar across regions, research indicates that the concentration of emitted pollutants can vary based on the type of vegetation burned. This suggests that local environmental characteristics—including plant species and landscape features—can influence the health impact of exposure to wildfire smoke^34,35^.

The Pantanal’s vegetation is highly diverse due to the biome’s dynamic hydrological patterns, particularly seasonal flooding. It includes aquatic, emergent, submerged, and floating plant species, as well as drier zones with open grasslands and closed forests. The region has a tropical climate characterized by a rainy summer and a dry winter.

In contrast, California—another fire-prone region—has a markedly different vegetation profile. Its landscape includes chaparral (dense, shrubby vegetation) and coastal savannah with stunted trees in nutrient-poor, acidic soils. California’s climate is drier and more prone to lightning storms and strong winds^2,36–39^.

Similarly, Australia features fire-adapted eucalyptus forests and grasslands that thrive in a hot, dry climate with intense seasonal droughts. Greece and other Mediterranean countries face frequent wildfires due to a combination of dense pine and maquis shrubland vegetation, prolonged summer droughts, and strong winds. In Canada, particularly in provinces like British Columbia and Alberta, boreal forests dominated by conifers are highly susceptible to large-scale wildfires exacerbated by dry lightning and increasingly warm summers^40–43^.

Despite these regional differences in vegetation and climate, numerous studies confirm that inhalation of wildfire smoke—especially at high concentrations—has serious health consequences for local populations. Research across different geographic settings has documented increased incidence of conditions such as asthma, pneumonia, and bronchitis, as well as elevated rates of cardiovascular-related deaths. These effects are more pronounced in areas with higher concentrations of air pollutants. Moreover, children and older adults appear to be especially vulnerable, with health risks varying by season^23–26,44,45^.

**Fig. 3:**
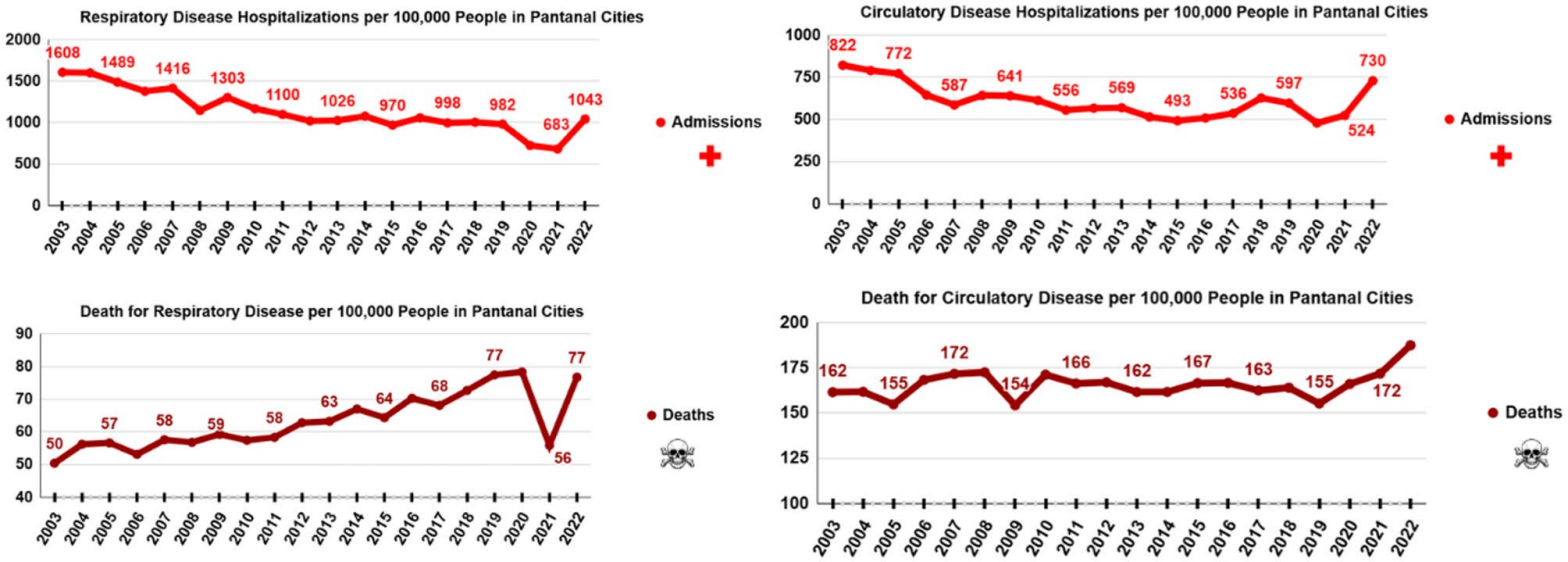
Long-term patterns in respiratory and cardiovascular morbidity and mortality per 100,000 residents in Pantanal municipalities. Source: Data from the DataSUS^46^.

Epidemiological data from the Pantanal, shown in Fig. 3, reveal a recent decline in hospitalization rates coupled with a notable increase in deaths attributed to respiratory and cardiovascular conditions. This trend suggests a possible rise in mortality linked to the inhalation of wildfire-related pollutants—a pattern also observed in global studies examining the health effects of exposure to wildfire smoke^23–26^.

Inhalation of pollutants present in wildfire emissions can trigger both local and systemic inflammatory responses that penetrate deeply into the human body. As a result, this poses health risks to all residents of the biome and surrounding areas, particularly those with pre-existing chronic respiratory conditions and vulnerable groups such as children and the elderly^23,45,47^.

The Pantanal biome experiences a drier climate from July onward, coinciding with the southern hemisphere winter (June to August). This seasonal dryness, combined with an increased frequency of forest fires, contributes to a rise in disease incidence. Moreover, the biome’s flat terrain, encircled by mountains, creates a greenhouse effect where smoke accumulates, further intensifying the adverse health impacts of these fires^36,48–50^.

This study aimed to assess the additive effect of increasing forest fire activity on hospitalizations for respiratory and cardiovascular diseases. To do so, we employed a generalized linear model (GLM) using data from the Brazilian Unified Health System alongside NASA geospatial fire data. This approach allowed us to analyze the health impacts of fires on populations living in and around the biome.

The findings of this research are highly relevant to the region’s inhabitants, as they can guide public health management strategies to prepare for potential surges in hospital demand and ensure adequate healthcare provision.

### Datasets

The datasets used in this study were compiled from multiple sources. Health-related data were obtained from Brazil’s Unified Health System (SUS), which encompasses the entire national healthcare infrastructure. Population data and ZIP code information were sourced from the Brazilian Institute of Geography and Statistics (IBGE), the official agency responsible for socioeconomic statistics. Data on atmospheric particles and fire outbreaks were retrieved from the National Aeronautics and Space Administration (NASA).

These datasets cover the period from 2010 to 2019 and include daily records from 21 municipalities within the Pantanal biome. Hospital admission data were accessed through DataSUS, specifically the Hospital Information System (SIH), a subsystem of SUS. This dataset contains detailed information such as admission and discharge dates, ICD-10 diagnoses, and patient demographics (age, sex, race, and ZIP code), enabling the geolocation of cases. According to the 2019 National Health Survey conducted by IBGE, this dataset represents 71.6% of healthcare coverage in Mato Grosso do Sul and 80.2% in Mato Grosso^10,46^.

Hospital admissions for respiratory diseases were identified using ICD-10 Chapter X codes (J00–J99), which include diagnoses such as pneumonia, bronchitis, and asthma. Similarly, circulatory system diseases were classified based on Chapter IX codes (I00–I99), encompassing conditions like pulmonary embolism, cerebral infarction, and heart failure^10,46^.

Since hospitalizations for respiratory diseases often rise when temperatures and humidity levels drop, a seasonal variable was introduced to account for these effects. Seasons were defined as follows: summer (December to February), fall (March to May), winter (June to August), and spring (September to November).

Geospatial data—including ZIP code coordinates, municipal boundaries, population estimates by year, and biome shapes—were obtained from IBGE, drawing from the Demographic Census and the National Register of Addresses for Statistical Purposes^10^.

Data on active fire outbreaks and air quality covariates (PM_2.5_, PM_10_, NO_2_, SO_2_), as well as climate variables (temperature and relative humidity), were sourced from NASA products. For each ZIP code, a spatial buffer of approximately 4,071.5 km² was created to extract and interpolate environmental data from at least the nine nearest observation points. These estimates were then assigned to the corresponding ZIP codes.

Active fire data were obtained from the MODIS 6 Hotspot Active Fire Detections collection (MCD14DL-NRT, 1 km² grid), distributed via the Fire Information for Resource Management System (FIRMS). Particulate matter concentrations (PM_2.5_ and PM_10_), sulfur dioxide (SO_2_) surface mass concentrations, average air temperature, and relative humidity were derived from the MERRA-2 Collection (0.25-degree grid), provided by the Goddard Earth Sciences Data and Information Services Center (GES DISC). Nitrogen dioxide (NO2) surface mass concentrations were retrieved from the OMI/Aura NO_2_ Total and Tropospheric Column L3 V3 dataset (0.25-degree grid), also distributed by GES DISC^51–55^.

To better capture smoke exposure, we incorporated both daily pollutant concentration levels and cumulative exposure metrics. For each ZIP code, daily average concentrations of PM_2.5_, PM_10_, SO_2_, and NO_2_ were included as continuous variables in the model. Additionally, to assess the effects of prolonged exposure, moving averages over 3, 5, and 7 days were calculated for sensitivity analyses. This approach enabled evaluation of both immediate pollutant peaks and the health impacts of sustained air pollution. These pollutant metrics, combined with daily fire counts from MODIS, served as proxies for population-level smoke exposure. While satellite data do not directly measure smoke plumes, the integration of active fire detections with pollutant concentrations provides a reasonable estimate of exposure intensity and duration at the municipal level.

The final dataset was aggregated daily by patient ZIP code, resulting in a time series of cross-sectional data for 2,902 ZIP codes over 3,652 days.

## Methods

This study examined the association between active forest fires and daily hospital admissions for respiratory and circulatory system diseases using Poisson regression models with distributed lag nonlinear models (DLNM) to capture delayed exposure-response effects^56–60^.

Daily hospital admissions for respiratory and circulatory diseases were analyzed separately by ZIP code. The daily count of active fire outbreaks per ZIP code was quantified as the main exposure variable. To reduce potential confounding, the models included air quality covariates—specifically concentrations of PM_2.5_, PM_10_, SO_2_, and NO_2_ (measured in µg/m^3^)—as well as climate variables such as average temperature (°C) and relative humidity (%). Seasonal variation was controlled by incorporating a seasonal factor (using summer as the reference) and a smoothing function to adjust for long-term temporal trends. Spatial variability was accounted for by including the longitude and latitude of ZIP code centroids.

Separate Poisson regression models were used to estimate the relative risk of hospital admissions associated with daily active fire outbreaks for both respiratory and circulatory diseases. Two time frames were analyzed—2010 to 2019 and 2015 to 2019—and a simplified equation was applied to quantify these relationships.

$${\text{Log}}\left[ {{\text{E}}\left( {{{\text{y}}_{\text{i}}}} \right)} \right]={\beta _0}\,+\,{\beta _{\text{1}}}{{\text{X}}_{\text{i}}}+\sum {\gamma _{\text{j}}}{{\text{Z}}_{{\text{ij}}}}$$

The model estimates the daily probability rate of hospital admissions, E(y_i_), for each ZIP code (i). The equation includes the following components:

- X_i_: Represents the smoothed lagged effect function of active fire outbreaks.
- Z_ij_: Encompasses a set of covariates, seasonal factors, and fixed effects components indexed by (j).

The parameters estimated are: β_0_ (intercept), β_1_ (active fire outbreaks), and γ_j_ (covariates, seasonal, and fixed effects).

A linear “cross-basis” function, S (Fire_i_, df), was applied to smooth the effect of active fire outbreaks. This function accounts for the daily count of active fires in ZIP code (i) and their lagged effects over a period of up to 10 days, modeled using a fourth-order polynomial. Air pollutant covariates (PM_2.5_, PM_10_, SO_2_, NO_2_) were modeled using the same smoothing approach.

For daily mean temperature, a lagged effect was modeled using two strata: lag 0 and lag 1–3 days, assuming constant effects within each stratum, measured per 1 °C increase. Relative humidity was included as a linear term without lagged effects or transformation. Seasonal variation was represented by dummy variables for fall, winter, and spring, with summer serving as the reference category.

Long-term temporal trends were controlled using a natural spline with degrees of freedom set to 5 or 10, depending on the length of the dataset (5 or 10 years). Finally, the longitude and latitude of ZIP code centroids (Lon_i_ and Lat_i_) were included as additional covariates in Z_ij_.

The predicted effect of active fire outbreaks on the relative risk (RR) of daily hospital admissions was calculated by estimating the increase in RR associated with an increment of 10 active fire outbreaks, accumulated over a 10-day lag period. The excess relative risk (ERR) was then derived by subtracting 1 from the RR. Predictions and their 95% confidence intervals were estimated separately for respiratory and circulatory disease groups, and for two time intervals: 2010–2019 and 2015–2019.

**Fig. 4:**
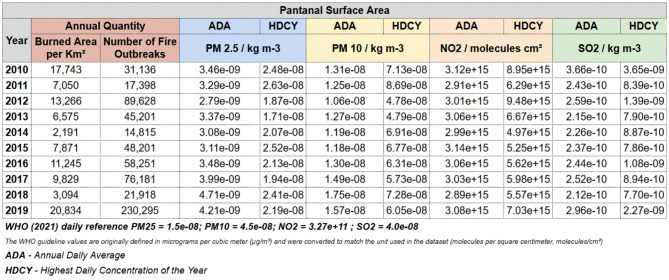
Fire outbreaks, air pollutants, and air quality trends in the Pantanal biome (2010–2019)^61^.

The selection of time windows in this study is closely linked to the intensity of fire outbreaks recorded in the Pantanal biome during the study period, as well as to the population’s exposure to airborne particles during these events, as illustrated in Fig. 4. From 2010 to 2019, a total of 633,024 fire outbreaks were documented. Notably, the five-year period from 2015 to 2019 accounted for 434,846 of these outbreaks—the highest concentration recorded in any equivalent timeframe within the historical series. Of particular significance is the year 2019, which experienced a 414.55% increase in fire outbreaks compared to the average from 2010 to 2018, indicating a substantial intensification of anthropogenic pressures and/or environmental conditions favorable to fire occurrence in the region^16^.

**Fig. 5:**
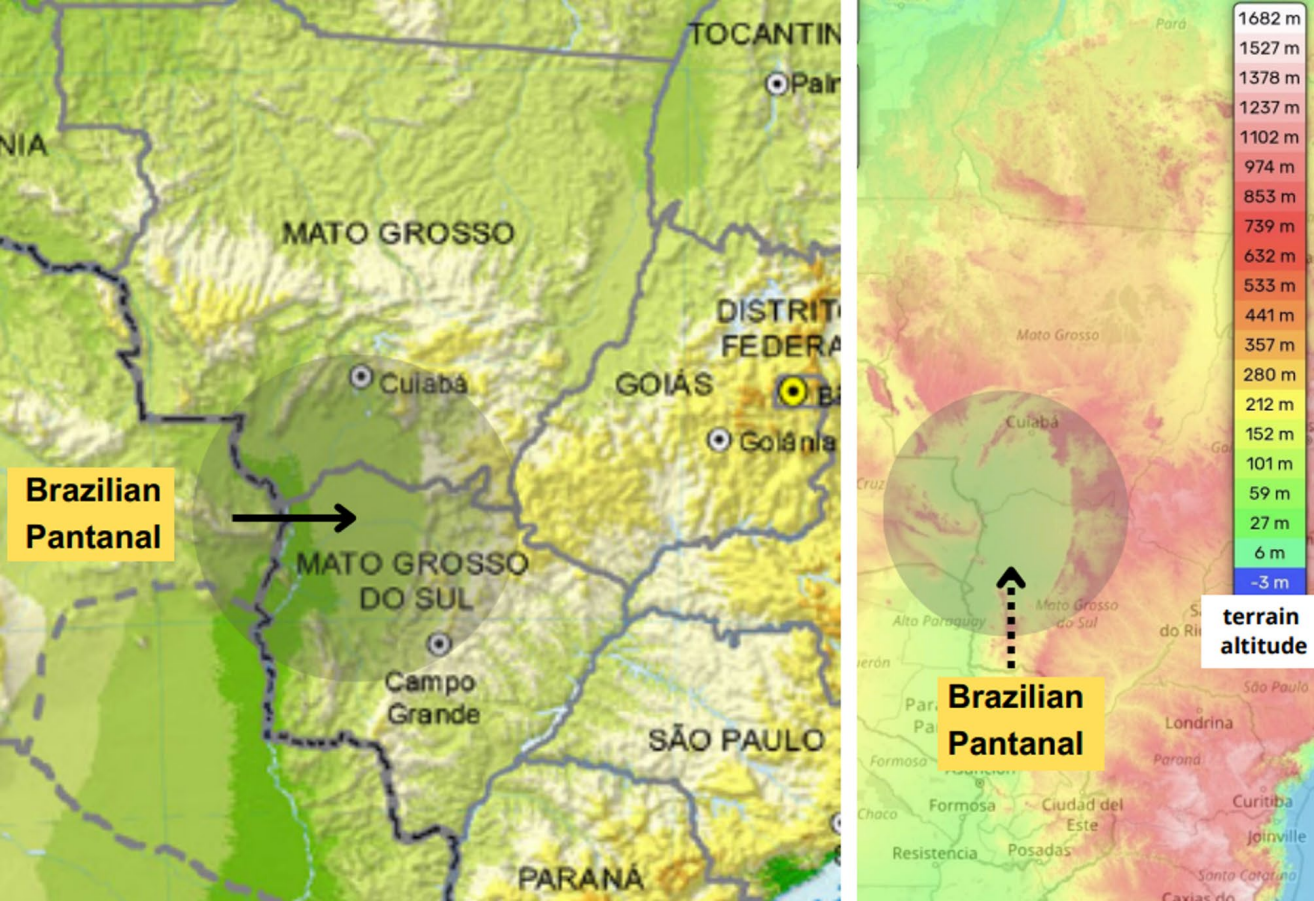
Terrain and topography of the Brazilian Pantanal. Map showing the mountainous region of the Pantanal. Adapted from IBGE (used under a CC BY 4.0 license) and OpenStreetMap data (OpenStreetMap contributors), used under the Open Database License (ODbL) – https://www.openstreetmap.org/copyright. Map modified by the authors through graphic editing.

Statistically significant correlations were observed between active fire outbreaks and concentrations of air pollutants—PM_2.5_, PM_10_, SO_2_, and NO_2_ (measured in µg/m^3^)—as well as dust levels. Additionally, these air pollutant concentrations, which were included as air quality covariates in the model, along with climate variables (average temperature in °C and relative humidity in %), showed significant associations with hospital admissions. The wind variable was not included in this study due to the unique geography of the Pantanal. The region’s flat terrain, encircled by mountains, tends to trap smoke from fires within the area, as depicted in Fig. 5.

The unique topography of the Pantanal, illustrated in Fig. 5, plays a significant role in shaping wind patterns and atmospheric dispersion. Previous studies examining the effects of topography and wind on the dispersion of CO_2_ leakage have shown that this pollutant can persist in the atmosphere even under strong wind conditions. Moreover, the Pantanal’s terrain can create a “trapping” effect that confines wind circulation within the region, leading to the buildup of hot, polluted air^62–64^.

Although this study did not directly investigate CO_2_ dispersion, the findings have important implications for other pollutants. Given that CO_2_—a lighter gas—can be trapped despite its greater susceptibility to wind transport, it follows logically that heavier gases, such as those analyzed in this study, would be similarly affected by this trapping phenomenon^63,64^.

The statistical model employed here has been previously used and validated in studies conducted in Turkey between 2013 and 2017, which utilized comparable variables and analytical methods^44,65^. All statistical analyses were performed using R within the RStudio environment. Additionally, to visually illustrate the annual variation of fire outbreaks in the Pantanal, maps were created based on NASA and IBGE data. These maps, generated from the analyzed data, are presented in the Results section.

## Results

**Fig. 6:**
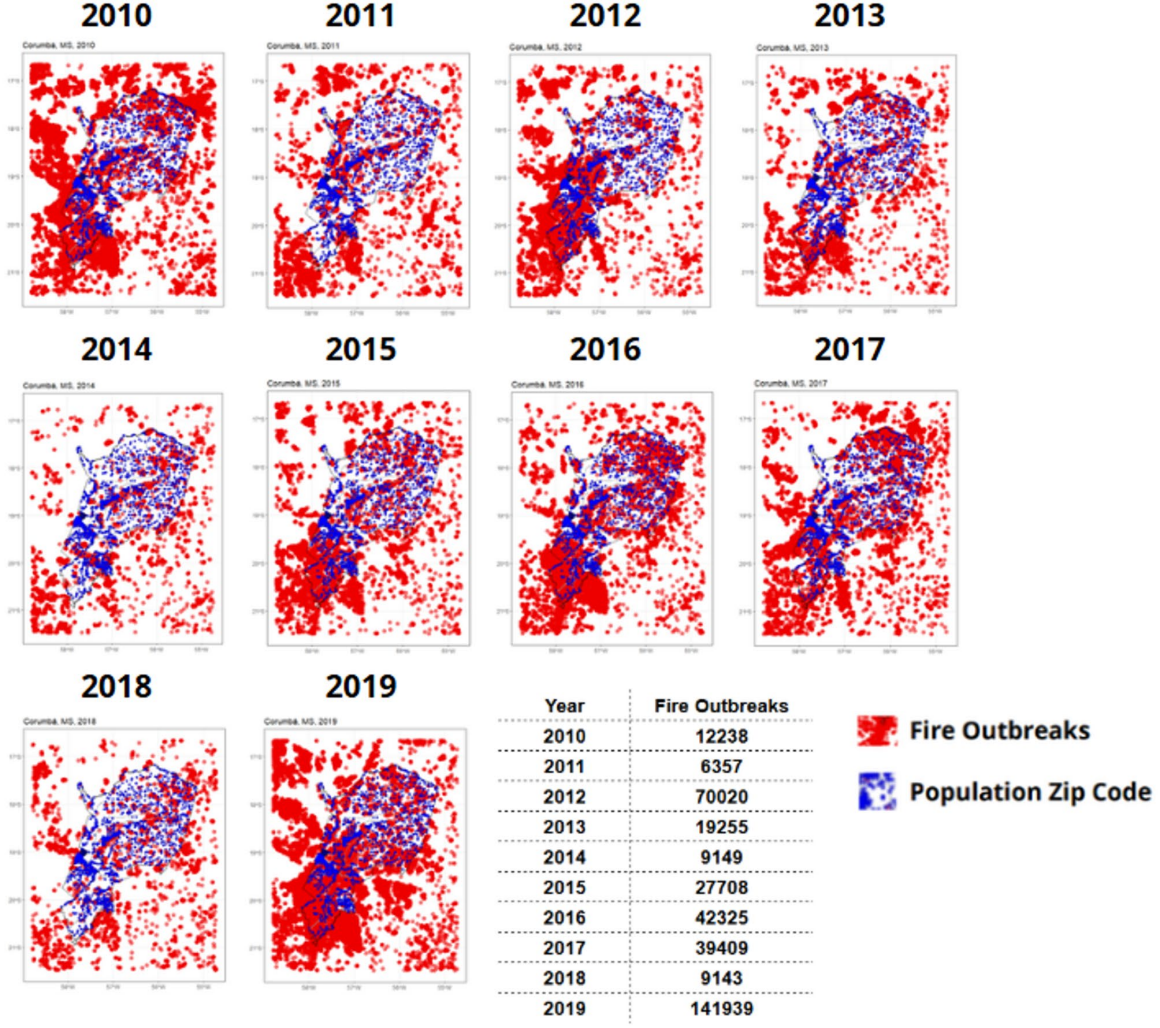
Temporal distribution of fire outbreaks in Corumbá, 2010–2019. Data source: Data from the IBGE and NASA data^10,54^.

To better interpret the results of the statistical analysis, it is essential to examine the pattern of fire outbreaks in the Pantanal biome over the decade from 2010 to 2019. Figure 6 presents this pattern for Corumbá, the primary municipality within the biome, which contains the largest share of its total area.

The Pantanal spans 21 municipalities. Corumbá alone accounts for 44.74% of the biome’s area, making it the largest municipality in the region. Poconé and Cáceres follow, covering 10.11% and 10.21% of the area, respectively, while the remaining 18 municipalities together comprise 34.94%. Figure 6 specifically depicts the annual fire outbreaks recorded in Corumbá^66^.

An analysis of forest fire activity in the municipality of Corumbá, as shown in Fig. 6, reveals a general upward trend in fire outbreaks over the years, with a notable peak occurring between 2015 and 2019. This pattern is also observed across all other municipalities within the Pantanal biome, as illustrated in the Supplementary Material (pages 21 to 41, Figures S1 to S21). Additional figures in the Supplementary Material (Figures S22 and S23) further detail the temporal and spatial dynamics of fire activity in Corumbá, using aggregated spatial grids to enhance visualization of yearly variations.

The variability in fire activity, depicted in Fig. 6 and Figures S1 to S21, led to significant fluctuations in air quality indicators over the 3,652-day analysis period. Notably, PM_10_ concentrations exceeded 13.31 µg/m^3^ on 25% of the days—approaching the World Health Organization (WHO) recommended limit. Furthermore, relative humidity fell below 68.83% on 50% of the days and dropped below 52.57% on 25% of the days—both values falling short of the WHO’s recommended minimum of 70%^67^.

**Fig. 7:**
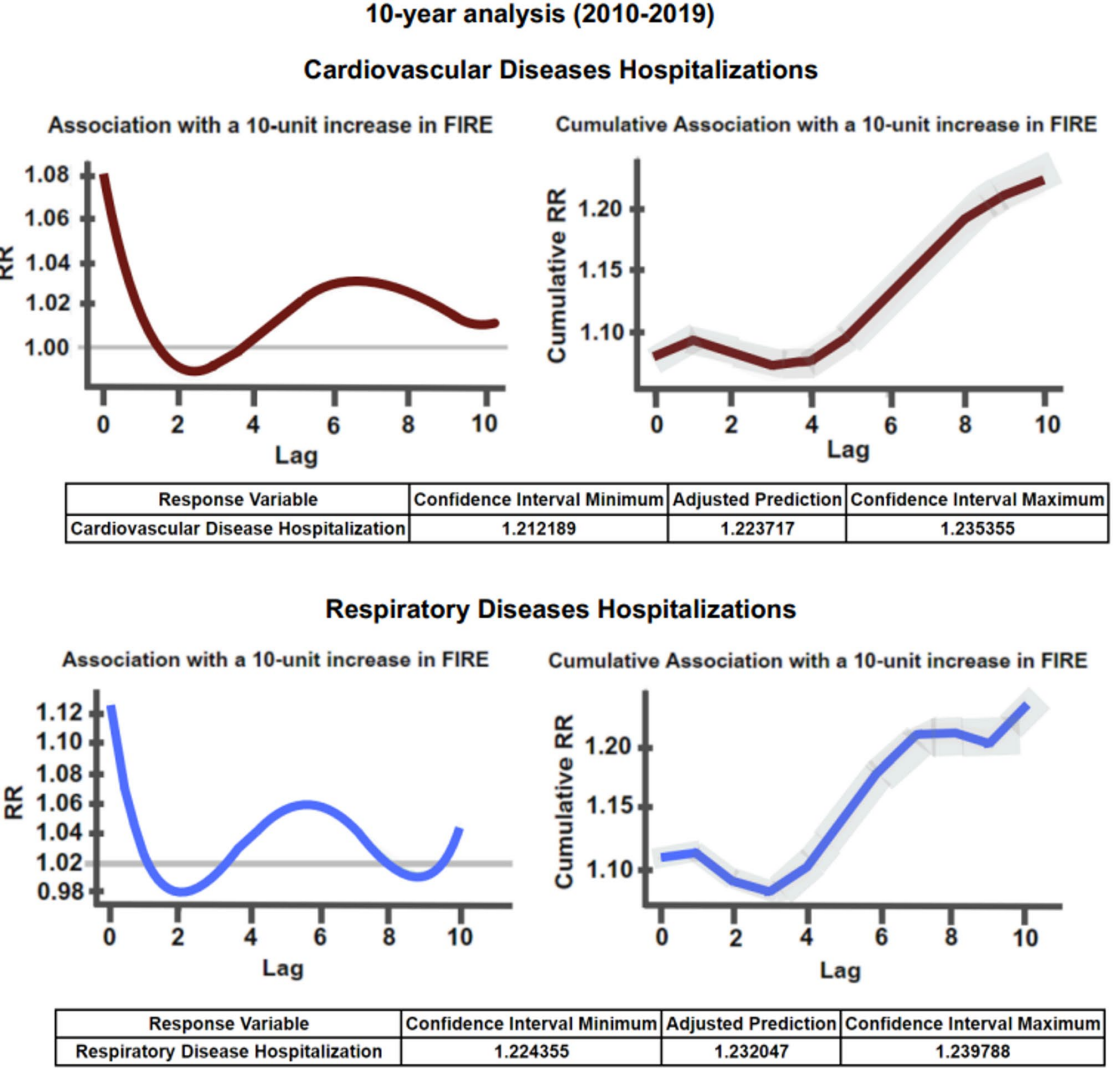
Relative risk of hospital admissions for respiratory and cardiovascular diseases associated with fire hotspots (10-Day Lag).

Additional evidence suggesting a potential link between forest fires and hospitalizations is found in the daily maximum hospitalization data. On certain days, up to 169 hospitalizations were recorded for respiratory diseases and 114 for cardiovascular diseases. These descriptive statistics underscore the importance of evaluating the relative risk that forest fires may pose in increasing hospital admissions for respiratory and cardiovascular conditions, as presented in Fig. 7.

As illustrated in Fig. 7, the 10-year data analysis (2010–2019) reveals an adjusted relative risk of 1.232 for hospitalizations due to respiratory diseases. This corresponds to a 23.20% increase in the risk of respiratory-related hospital admissions and an estimated 22.37% increase in the risk of cardiovascular-related hospitalizations among residents of the 21 municipalities that make up the Pantanal biome.

These findings are consistent with those of a previous nationwide study in Brazil on the health impacts of forest fires. That study estimated a 23% increase (95% CI: 12–33%) in the risk of respiratory hospitalizations and a 21% increase (95% CI: 8–35%) in cardiovascular hospitalizations associated with wildfire exposure between 2008 and 2018—closely aligning with the results observed in our analysis^68^.

A focused analysis of the 2015–2019 period, which corresponds to a time of heightened fire activity in the biome (as shown in Fig. 7), reveals even more pronounced impacts: the relative risk of cardiovascular hospitalizations increased by 29.30%, while the risk for respiratory diseases rose by 33.84%. When seasonal variations are excluded and only respiratory diseases are analyzed, the effect is slightly stronger, with the risk rising to 34.11%—reaching a peak upper limit of 35.34%.

To contextualize these risks in absolute terms, we compared the average number of hospitalizations on wildfire-affected days to baseline levels on days without fire activity, applying a 10-day lag. On average, non-fire days recorded 1.53 hospitalizations for circulatory diseases and 2.26 for respiratory diseases in the municipality of Corumbá. In contrast, during wildfire-affected periods, these numbers surged to 28.36 for circulatory diseases and 40.25 for respiratory diseases—representing increases of approximately 1,754% and 1,680%, respectively. This stark contrast underscores the substantial burden that wildfire exposure may impose on local healthcare systems.

Taken together, the findings of this study—when considered alongside the demographic characteristics of the Pantanal biome and previous research^22,44,45^—provide strong evidence that increasing forest fire activity has a detrimental impact on the health of populations living in and around the biome.

## Discussion

The main findings of this study reveal a significant increase in hospitalization rates for respiratory and cardiovascular diseases as a result of forest fires. These outcomes underscore the urgent need for both society and public authorities to strengthen the healthcare system’s capacity to respond to large-scale environmental crises, such as those experienced in the Pantanal biome in Brazil during the final months of 2024.

The study also highlights the importance of addressing the harmful effects of the expansionist logic that dominates the agricultural economy surrounding the Pantanal. The relentless pursuit of profit in this sector puts the entire local population at risk and substantially contributes to climate change—a pressing issue not only for Brazil but for the global community as well.

Importantly, the study’s findings are consistent with those of other key research on the health impacts of forest fires, particularly a study conducted in California, which examined the effects of large-scale wildfires in 2003 on hospitalization rates for respiratory and cardiovascular illnesses^22^.

Both this and the California study conclude that forest fires lead to increased hospitalizations for respiratory conditions. However, there is a notable difference in the scale of impact observed, which may be attributed to two main factors:

1. Geographic characteristics: California’s mountainous landscape facilitates smoke dispersion, whereas the Pantanal—a flat region encircled by mountains—tends to trap smoke, creating a “greenhouse effect” that exacerbates exposure.
2. Vegetation type: The composition of forest vegetation in California differs significantly from that of the Pantanal, potentially contributing to variations in health outcomes.

Similar dynamics have been reported in other fire-prone regions, such as Australia, where eucalyptus-dominated forests combined with prolonged droughts have led to frequent and intense wildfires, and Canada, where coniferous boreal forests are highly vulnerable to fire events triggered by dry lightning and heatwaves. In Mediterranean countries like Greece, densely vegetated pine and shrubland landscapes, along with strong summer winds and dry climates, have resulted in repeated wildfire episodes affecting both human health and infrastructure^40–43^.

These international comparisons reinforce the global relevance of understanding how local geography, vegetation, and policy contexts shape the health outcomes of wildfire exposure.

Additionally, studies supporting this analysis show that children aged 0 to 4 and elderly individuals aged 65 and over are the most vulnerable to illnesses caused by exposure to smoke from forest fires^22,44,45^. Within the 21 municipalities of the Pantanal, these age groups represent a substantial portion of the population: children under 5 account for 7.43% (54,328 individuals), while those aged 65 and over make up 9.33% (68,236 individuals). This demographic profile further underscores the heightened public health risks posed by the spread of wildfires in the region.

While the primary focus of this study is the health impact of inhaling pollutants released by burning organic forest material, it is crucial to situate these findings within the broader context of economic transformation in the Pantanal biome. The region is facing mounting pressure from expanding agricultural frontiers, driven largely by soybean cultivation, the intensification of cattle ranching, the rise of mining operations, increasing tourism, and the development of large-scale infrastructure projects such as roads and waterways. These activities have led to the widespread conversion of natural landscapes into zones of economic exploitation, making the Pantanal a focal point for capital-driven development.

Moreover, the weakening of environmental regulations—accelerated since 2018—alongside government policies that favor agribusiness, has further eroded mechanisms for environmental protection. This has placed the Pantanal—a biome historically underprotected—on a collision course with development models that disregard its ecological vulnerability and the urgent need for conservation. The consequences of this dynamic go far beyond environmental degradation: they directly affect local populations, threaten biodiversity, and elevate the health risks associated with smoke pollution from frequent and widespread fires.

### Study limitations

This study employed the most relevant and accessible short-term data sources available. A key methodological feature was the integration of patient postal code data from DataSUS with centroid data from the Brazilian Institute of Geography and Statistics (IBGE). This approach allowed for a reasonably accurate spatial correspondence with NASA’s climate and air quality datasets.

Working with sparse and large-scale data presents inherent challenges. NASA’s datasets are modeled at the global level, with active fire events defined accordingly. These global definitions may lead to underestimations or overestimations of active fire outbreaks within the specific context of the Pantanal biome.

Addressing this limitation would require the delineation of localized boundaries and/or in-situ validation, both of which were beyond the scope of this study.

The zip code-level data aggregated from DataSUS serves as the closest available proxy to individual-level health data. Although this introduces a degree of imprecision, it represents a substantial improvement over traditional studies that rely on municipality-level data aggregated monthly. Nonetheless, variation in the size of zip code areas may introduce a scale bias. In particular, larger zip codes could register greater effects simply due to encompassing larger areas potentially affected by fire activity.

The analytical model includes both active fire outbreaks and concentrations of particulate matter (PM_2.5_ and PM_10_), allowing for the isolation of the effects of particulate pollutants even on days without fires. Further refinement could involve modeling the relationship between active fires and particulate matter using regression analysis, especially for populations concentrated in central urban areas.

Properly defining the smoothing functions and time lag effects for each parameter would require an extensive sensitivity analysis using various time lags and degrees of freedom for the polynomial terms. While such an exercise would enhance the robustness of the results, it exceeds the computational resources currently available.

Despite these limitations, this study makes an important scientific contribution by approximating a quasi-controlled study and individualizing exposure to forest fire smoke. Nevertheless, a more rigorous controlled study involving a smaller patient cohort and low-level toxin exposure measurements could more precisely determine the health effects of emissions from burning biological material.

Another valuable direction for future research would be to integrate this analysis with a study on wind dynamics and smoke transport in the region, accounting for the unique topographical features of the Pantanal. To date, studies specifically examining these atmospheric and geographical interactions are lacking. It is hypothesized that the region’s topography contributes to a “greenhouse effect,” trapping smoke and exacerbating exposure. A dedicated investigation into this phenomenon could offer deeper insights into how smoke dispersion patterns may increase health risks for populations located beyond the immediate vicinity of the biome.

## Conclusion

This study aimed to demonstrate the additive impact of forest fires on hospital utilization and found evidence supporting this relationship. These findings highlight the need for public health authorities in the region to be prepared, enabling them to anticipate and manage potential surges in demand for healthcare services.

It is important to recognize that forest fires can stem from various causes, including arson and the region’s climatic characteristics. Regardless of their origin, addressing wildfires requires effective policy interventions by government authorities. When fires result from arson, it is the state’s responsibility to combat and prevent such criminal activity. Conversely, when fires are driven by climatic conditions, the state must ensure that the local population is adequately informed about the associated risks.

Beyond the health consequences, wildfires also have significant economic repercussions. In the Pantanal, for example, smoke from fires can severely disrupt agricultural activities, illustrating the wide-ranging effects of these events. This underscores the urgent need for integrated public policies that simultaneously address environmental degradation and promote sustainable economic development.

No matter the cause, the consequences are profound: surrounding communities, along with the region’s rich biodiversity, including its unique flora and fauna, suffer greatly. For this reason, it is imperative that local, state, and federal governments invest in strengthening environmental monitoring and enforcement agencies, enact and uphold protective legislation, and allocate sufficient resources to prepare the regional health system to respond effectively to such events.

Political action in the Pantanal must prioritize prevention to mitigate the risk of extreme climatic events. Notably, in 2024, the area affected by fires in the Pantanal biome increased by 529% compared to the average of previous years, leading to devastating outcomes such as biodiversity loss, habitat destruction, and serious health threats to local populations^69,70^.

The frequency of such extreme events appears to be rising. Following the 2024 fires in the Pantanal, a major wildfire in California, USA, in early 2025 received widespread international coverage, resulting in substantial property damage and loss of life.

Many scientists argue that these events are directly linked to the effects of climate change, itself a consequence of the imbalance between the exploitation and conservation of natural resources. Researchers warn of an increasing frequency of such disasters, with growing numbers of victims worldwide.

This context reinforces the vital role of forest protection in counteracting the harmful effects of an expansionist economic model. Recognizing the intrinsic value of forests has never been more urgent. From both a Brazilian and global perspective, forests must be regarded as one of the planet’s most invaluable assets. These ecosystems hold immense scientific potential yet to be discovered, making their preservation a shared responsibility for all.

## Supplementary Information

Below is the link to the electronic supplementary material.

Supplementary Material 1

## Data Availability

All data are available in the manuscript or the supplementary materials.If you need to check the raw data, you can access it publicly at the following URLs: https://datasus.saude.gov.br/transferencia-de-arquivos/ (for health data). https://giovanni.gsfc.nasa.gov/giovanni/ (for climate data). https://www.ibge.gov.br/estatisticas/sociais/populacao/38734-cadastro-nacional-de-enderecos-para-fins-estatisticos.html?=&t=downloads (for zip codes).
