# Supplementary material for: Additive effect of wildfires on hospital admission in the Pantanal wetland, Brazil
Source: Sci Rep. 2025 Jul 29;15:27572. doi: 10.1038/s41598-025-13257-z (PMC12307731; doi:10.1038/s41598-025-13257-z)

scientific reports

Supplementary Materials for

Additive Effect of Wildfires on Hospital Admission in the Pantanal Wetland, Brazil

Corresponding author: andre.calixto.goncalves@gmail.com

André Calixto Gonçalves; Marcelo Marques de Magalhães; Gustavo Andrey de Almeida Lopes Fernandes; Ivan Filipe Fernandes; Kaylane de Almeida Faria; Gabriel Alexandre dos Santos; Rodolfo Valentim; Gabriela Moraes do Nascimento; Francisco Aparecido Rodrigues; Ricardo Ceneviva; Maria Clara Mendes Stama; Daniel Tetsuo G. Mori; Carolina Nascimento Capellini; Maria Eduarda Feres Garcia; Thiago Bruschi; Gabriel Poveda Gonçalves; Laís Costa Brito; Djeansy Djarny Etchiamiadzy Toussaint ; Rejane Calixto Gonçalves; Olinda do Carmo Luiz.

Descriptive Statistics

Table S1. - Table Captions

Attribute	Description	Factors and data
**date_in**	Admission date	2010-01-01 to 2019-12-31 (YYYY-MM-DD)
**zipcode**	Zip code	2,902 zip codes of 21 municipalities of Pantanal Biome
**code_muni**	IBGE 7 digits municipality code	21 municipalities of Pantanal Biome
**sex**	Sex of the patient	Factor w/ 2 levels:<br>1 "Female"<br>2 "Male"
**race**	Race of the patient	Factor w/ 6 levels:<br>1 = "White"<br>2 = "Black or Afican American<br>3 = "Asian"<br>4 = "Indigenous<br>5 = "Other"<br>6 = "Unknown
**age5c**	Age of the patient	Factor w/ 5 levels (years):<br>1 "00-02"<br>2 "03-14"<br>3 "15-39"<br>4 "40-59"<br>5 "60+"
**rdh_n_no**	Admissions for non-respiratory diseases	Daily sum of admissions per 100,000 inhabitants
**rdh_n_yes**	Admissions for respiratory diseases	Daily sum of admissions per 100,000 inhabitants
**rdh_days_yes**	Days of hospitalization for respiratory diseases	Sum of days of hospitalization
**cdh_n_no**	Admissions for non-cardiovascular diseases	Daily sum of admissions per 100,000 inhabitants
**cdh_n_yes**	Admissions for cardiovascular diseases	Daily sum of admissions per 100,000 inhabitants
**cdh_days_no**	Days of hospitalization for non-cardiovascular diseases	Sum of days of hospitalization
**cdh_days_yes**	Days of hospitalization for cardiovascular diseases	Sum of days of hospitalization
**pm10**	Dust surface mass concentration PM10	Daily mean concentration (mcg.m-3) (MERRA-2 tavg1_2d_aer_Nx V5.12.4)
**pm25**	Dust surface mass concentration PM2.5	Daily mean concentration (mcg.m-3) (MERRA-2 tavg1_2d_aer_Nx V5.12.4)
**so2**	SO2 surface mass concentration	Daily mean concentration (mcg.m-3) (MERRA-2 tavg1_2d_aer_Nx V5.12.4)
**no2tt**	Total NO2 surface mass concentration	Daily mean concentration (mcg.m-3) calculated from the vertical NO2 column density (OMI/Aura L3 V3)
**no2tp**	Tropospheric NO2 surface mass concentration	Daily mean concentration (mcg.m-3) calculated from the tropospheric NO2 column density (OMI/Aura L3 V3)
**rh2m**	Relative humidity at 2 meters	Daily mean relative humidity at 2 meters (%) (NASA/POWER CERES/MERRA2 Native Resolution Daily Data)
**t2m**	Temperature at 2 meters	Daily mean temperature at 2 meters (Celsius) (NASA/POWER CERES/MERRA2 Native Resolution Daily Data)
**fire**	Active fire hotspots	Daily number of hotspots (FIRMS, MODIS, MCD14DL-NRT V006)
**lon**	Zip code centroid longitude	Longitude (degrees)
**lat**	Zip code centroid latitude	Latitude (degrees)

footnote: $PM_{2.5}$ and PM_{10} data were derived from the MERRA-2 product, specifically using the variables related to dust surface mass concentration. As such, they represent only the fraction of airborne mineral dust and do not include other sources of particulate matter, such as biomass burning, vehicular, or industrial emissions. Therefore, these variables reflect exposure to dust rather than total ambient particulate matter.

Descriptive Statistics

Table S2. Hospitalizations and air quality indicators

Hospitalizations and air quality indicators									
**Variable**	**n**	**Mean**	**SD**	**SE**	**p0**	**p25**	**p50**	**p75**	**p100**
Admissions for non-respiratory diseases	1123944.01	307.85	85.31	1.41	37.83	243.38	310.16	370.21	605.55
Admissions for respiratory diseases	146959.23	40.25	21.72	0.36	0.00	24.28	36.65	52.58	169.19
Admissions for non-cardiovascular diseases	1167353.17	319.74	88.01	1.46	37.83	252.35	320.44	383.18	636.50
Admissions for cardiovascular diseases	103550.07	28.36	16.77	0.28	0.00	16.30	25.19	37.28	113.75
Active fire hotspots	3652	0.53	1.57	0.03	0.00	0.00	0.03	0.38	26.51
Dust surface mass concentration PM10	3652	10.35	9.89	0.16	0.06	3.80	7.40	13.31	99.65
Dust surface mass concentration PM2.5	3652	2.82	2.60	0.04	0.02	1.09	2.07	3.63	29.36
SO2 surface mass concentration	3652	0.24	0.23	0.00	0.02	0.13	0.19	0.27	3.93
Total NO2 surface mass concentration	3652	0.28	0.09	0.00	0.08	0.24	0.27	0.31	1.41
Tropospheric NO2 surface mass concentration	3652	0.09	0.08	0.00	0.01	0.05	0.07	0.10	1.20
Temperature at 2 meters	3652	26.41	2.71	0.04	14.57	24.96	26.47	27.97	35.15
Relative humidity at 2 meters	3652	64.20	16.74	0.28	18.90	52.57	68.83	77.56	92.31

Table S3. Hospitalizations by age group

Hospitalizations by group (age)										
**Age of the patient**	**Variable**	**n**	**Mean**	**SD**	**SE**	**p0**	**p25**	**p50**	**p75**	**p100**
00-02	Admissions for non-respiratory diseases	54383.07	14.94	10.67	0.18	0.00	7.02	12.65	20.28	76.26
00-02	Admissions for respiratory diseases	34185.80	9.39	8.98	0.15	0.00	3.02	6.98	13.23	73.25
00-02	Admissions for non-cardiovascular diseases	87883.25	24.14	14.23	0.24	0.44	13.65	21.77	32.05	101.69
00-02	Admissions for cardiovascular diseases	685.62	0.19	1.19	0.02	0.00	0.00	0.00	0.00	19.98
03-14	Admissions for non-respiratory diseases	84203.70	23.11	14.63	0.24	0.00	12.34	20.79	30.60	123.64
03-14	Admissions for respiratory diseases	20877.33	5.73	7.32	0.12	0.00	0.47	3.15	8.63	74.63
03-14	Admissions for non-cardiovascular diseases	104098.50	28.57	16.76	0.28	0.43	16.06	25.82	37.97	124.11
03-14	Admissions for cardiovascular diseases	982.53	0.27	1.35	0.02	0.00	0.00	0.00	0.00	20.55
15-39	Admissions for non-respiratory diseases	495670.57	135.76	41.83	0.69	17.38	106.40	132.45	163.13	339.30
15-39	Admissions for respiratory diseases	18663.08	5.11	6.34	0.10	0.00	0.48	3.10	7.50	64.99
15-39	Admissions for non-cardiovascular diseases	503986.20	138.04	42.51	0.70	17.38	108.13	135.01	165.61	338.70
15-39	Admissions for cardiovascular diseases	10347.45	2.83	4.87	0.08	0.00	0.00	0.96	3.66	62.63
40-59	Admissions for non-respiratory diseases	240395.52	65.84	30.04	0.50	1.06	43.37	62.51	85.04	183.97
40-59	Admissions for respiratory diseases	20893.49	5.72	6.50	0.11	0.00	0.95	3.79	8.42	55.36
40-59	Admissions for non-cardiovascular diseases	230656.19	63.18	29.24	0.48	1.06	40.95	59.62	82.17	201.11
40-59	Admissions for cardiovascular diseases	30632.82	8.39	8.56	0.14	0.00	2.25	5.95	11.45	70.11
60+	Admissions for non-respiratory diseases	249291.16	68.28	29.61	0.49	4.61	46.37	65.39	86.87	199.41
60+	Admissions for respiratory diseases	52339.53	14.34	11.04	0.18	0.00	6.26	11.72	19.58	88.88
60+	Admissions for non-cardiovascular diseases	240729.04	65.94	28.80	0.48	3.62	44.93	63.55	83.22	195.41
60+	Admissions for cardiovascular diseases	60901.66	16.68	12.27	0.20	0.00	7.72	13.95	22.62	94.92

Table S4. Hospitalizations by gender

Hospitalizations by group (sex)										
**Sex**	**Variable**	**n**	**Mean**	**SD**	**SE**	**p0**	**p25**	**p50**	**p75**	**p100**
Female	Admissions for non-respiratory diseases	652948.01	178.84	55.05	0.91	15.27	137.71	178.25	217.38	443.86
Female	Admissions for respiratory diseases	65156.30	17.85	12.65	0.21	0.00	8.59	15.43	24.37	99.26
Female	Admissions for non-cardiovascular diseases	670750.64	183.72	56.19	0.93	15.27	141.71	182.55	221.46	440.60
Female	Admissions for cardiovascular diseases	47353.67	12.97	11.12	0.18	0.00	5.10	10.15	17.69	89.14
Male	Admissions for non-respiratory diseases	470996.01	129.00	43.94	0.73	5.72	97.05	127.04	156.95	300.12
Male	Admissions for respiratory diseases	81802.93	22.41	14.51	0.24	0.00	11.87	19.62	30.02	102.61
Male	Admissions for non-cardiovascular diseases	496602.53	136.02	45.30	0.75	5.10	102.05	133.47	165.86	301.58
Male	Admissions for cardiovascular diseases	56196.41	15.39	11.54	0.19	0.00	6.98	12.89	20.78	87.15

Descriptive Statistics

Table S5. Hospitalizations by race

Hospitalizations by group (race)										
**Race**	**Variable**	**n**	**Mean**	**SD**	**SE**	**p0**	**p25**	**p50**	**p75**	**p100**
Indigenous	Admissions for non-respiratory diseases	26484.91	8.17	6.12	0.11	0.00	3.75	6.42	11.16	46.92
Indigenous	Admissions for respiratory diseases	3837.76	1.18	2.33	0.04	0.00	0.00	0.00	2.11	23.71
Indigenous	Admissions for non-cardiovascular diseases	29128.88	8.98	6.32	0.11	0.00	4.12	7.70	12.12	46.92
Indigenous	Admissions for cardiovascular diseases	1193.78	0.37	1.33	0.02	0.00	0.00	0.00	0.00	14.53
Black	Admissions for non-respiratory diseases	27452.55	8.09	7.83	0.13	0.00	2.23	5.68	11.37	57.94
Black	Admissions for respiratory diseases	3141.49	0.93	2.47	0.04	0.00	0.00	0.00	0.00	33.08
Black	Admissions for non-cardiovascular diseases	28006.98	8.25	7.92	0.14	0.00	2.53	5.80	11.37	57.94
Black	Admissions for cardiovascular diseases	2587.06	0.76	2.25	0.04	0.00	0.00	0.00	0.00	35.38
Other	Admissions for non-respiratory diseases	760986.91	208.43	68.07	1.13	27.35	156.97	205.88	254.93	496.41
Other	Admissions for respiratory diseases	101528.78	27.81	16.24	0.27	0.00	15.54	25.41	37.14	116.12
Other	Admissions for non-cardiovascular diseases	793675.66	217.39	69.46	1.15	27.35	164.66	215.51	266.23	491.47
Other	Admissions for cardiovascular diseases	68840.04	18.86	12.95	0.21	0.00	9.55	16.53	25.02	101.00
White	Admissions for non-respiratory diseases	154101.31	42.22	22.50	0.37	0.43	25.71	38.43	55.61	163.33
White	Admissions for respiratory diseases	23263.05	6.37	8.36	0.14	0.00	0.00	3.69	9.11	92.72
White	Admissions for non-cardiovascular diseases	163098.69	44.68	23.56	0.39	1.51	27.52	41.41	58.05	212.84
White	Admissions for cardiovascular diseases	14265.67	3.91	6.39	0.11	0.00	0.00	1.10	5.02	72.89
Asian	Admissions for non-respiratory diseases	11256.71	6.42	6.04	0.14	0.00	2.18	4.42	8.85	87.77
Asian	Admissions for respiratory diseases	2717.86	1.55	2.89	0.07	0.00	0.00	0.00	3.61	33.74
Asian	Admissions for non-cardiovascular diseases	12703.46	7.24	6.52	0.16	0.00	3.06	5.33	10.86	87.77
Asian	Admissions for cardiovascular diseases	1271.11	0.72	2.03	0.05	0.00	0.00	0.00	0.00	32.84
Unknown	Admissions for non-respiratory diseases	143661.63	39.70	34.96	0.58	0.00	15.31	28.24	54.62	236.66
Unknown	Admissions for respiratory diseases	12470.30	3.45	6.41	0.11	0.00	0.00	0.50	3.93	62.25
Unknown	Admissions for non-cardiovascular diseases	140739.50	38.89	33.98	0.56	0.00	14.89	28.01	53.54	232.76
Unknown	Admissions for cardiovascular diseases	15392.42	4.25	7.61	0.13	0.00	0.00	1.07	5.35	74.64

Statistical analysis

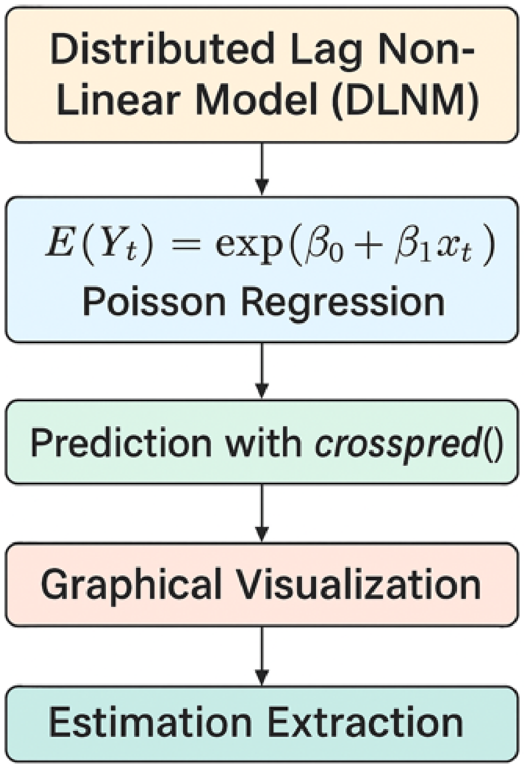

$$\log(E[Y|T)) = \alpha + \sum_k f_k(x_{k,t}, lags) + \beta_1 rh2m$$ [illegible]

$$\beta_2 \,\text{season}_t \; + s(t\,df) \; + \beta_3 \,lon \; + \beta_4 \,lat$$

Where:

- Y_t: number of hospital admissions on day t
- $\mathbb{E}[Y_t]$: expected value of Y_t
- $\log$: natural logarithm (link function for the Poisson distribution)
- α: intercept
- $f_k(x_{k,t}, \text{lags})$: cross-basis function representing the non-linear and lagged effect of predictor x_k (e.g., fire, PM10, PM2.5, SO_2, NO_2, temperature)
- rh2m_t: relative humidity at time t
- season_t: categorical variable for season
- $s(t; \text{df})$: smooth spline function of time with degrees of freedom (to adjust for long-term and seasonal trends)
- lon, lat: longitude and latitude (to adjust for spatial variation)

Statistical analysis

Distributed Lag Non-linear Models

Impact of emissions from forest fires on hospitalizations for cardiovascular diseases

With Control of Seasonality Bias - Cardiovascular Diseases (5 years analisys)

Table S6.

Coefficients:

##	Estimate	Std. Error	z value	Pr(>|z|)	
## (Intercept)	-4.569e+01	2.338e-01	-195.375	< 2e-16	***
## cb1.firev1.l1	1.112e-02	1.020e-04	108.974	< 2e-16	***
## cb1.firev1.l2	-1.599e-01	4.801e-03	-33.300	< 2e-16	***
## cb1.firev1.l3	6.102e-01	2.512e-02	24.294	< 2e-16	***
## cb1.firev1.l4	-8.204e-01	4.005e-02	-20.485	< 2e-16	***
## cb1.firev1.l5	3.612e-01	1.977e-02	18.270	< 2e-16	***
## cb1.pm10v1.l1	2.434e-01	9.778e-03	24.894	< 2e-16	***
## cb1.pm10v1.l2	-4.848e+00	1.862e-01	-26.041	< 2e-16	***
## cb1.pm10v1.l3	2.092e+01	8.067e-01	25.927	< 2e-16	***
## cb1.pm10v1.l4	-3.106e+01	1.250e+00	-24.855	< 2e-16	***
## cb1.pm10v1.l5	1.478e+01	6.298e-01	23.462	< 2e-16	***
## cb1.pm25v1.l1	-7.946e-01	3.912e-02	-20.310	< 2e-16	***
## cb1.pm25v1.l2	1.406e+01	7.391e-01	19.023	< 2e-16	***
## cb1.pm25v1.l3	-5.843e+01	3.191e+00	-18.310	< 2e-16	***
## cb1.pm25v1.l4	8.526e+01	4.934e+00	17.281	< 2e-16	***

Coefficients:

##	Estimate	Std. Error	z value	Pr(>|z|)	
## cb1.pm25v1.l5	-4.010e+01	2.484e+00	-16.147	< 2e-16	***
## cb1.so2v1.l1	-1.126e+00	3.124e-02	-36.044	< 2e-16	***
## cb1.so2v1.l2	1.118e+01	6.160e-01	18.153	< 2e-16	***
## cb1.so2v1.l3	-2.921e+01	2.883e+00	-10.134	< 2e-16	***
## cb1.so2v1.l4	3.057e+01	4.654e+00	6.569	5.08e-11	***
## cb1.so2v1.l5	-1.149e+01	2.395e+00	-4.799	1.60e-06	***
## cb1.no2v1.l1	-3.487e-01	8.126e-02	-4.291	1.78e-05	***
## cb1.no2v1.l2	-1.804e+00	1.356e+00	-1.331	0.183153	
## cb1.no2v1.l3	1.572e+01	5.943e+00	2.645	0.008160	**
## cb1.no2v1.l4	-2.711e+01	9.152e+00	-2.962	0.003053	**
## cb1.no2v1.l5	1.357e+01	4.543e+00	2.988	0.002812	**
## cb1.t2mv1.l1	-1.328e+01	2.608e-01	-50.936	< 2e-16	***
## cb1.t2mv1.l2	1.771e+01	3.528e-01	50.197	< 2e-16	***
## cb1.t2mv2.l1	-9.989e+00	2.638e-01	-37.867	< 2e-16	***
## cb1.t2mv2.l2	1.338e+01	3.566e-01	37.512	< 2e-16	***
## cb1.t2mv3.l1	-4.069e+00	2.093e-01	-19.438	< 2e-16	***
## cb1.t2mv3.l2	5.283e+00	2.805e-01	18.836	< 2e-16	***
## cb1.t2mv4.l1	-2.111e+01	5.532e-01	-38.149	< 2e-16	***

Coefficients:

##	Estimate	Std. Error	z value	Pr(>|z|)	
## cb1.t2mv4.l2	2.781e+01	7.459e-01	37.290	< 2e-16	***
## cb1.t2mv5.l1	1.114e-01	4.115e-01	0.271	0.786567	
## cb1.t2mv5.l2	-6.106e-01	5.465e-01	-1.117	0.263873	
## rh2m	-1.718e-03	4.706e-04	-3.650	0.000262	***
## seasonAutumn	4.730e-02	1.339e-02	3.533	0.000412	***
## seasonSpring	2.120e-01	1.521e-02	13.934	< 2e-16	***
## seasonWinter	9.211e-02	1.963e-02	4.692	2.70e-06	***
## ns(time, 7)1	2.485e-01	3.060e-02	8.123	4.54e-16	***
## ns(time, 7)2	-2.647e-01	3.990e-02	-6.635	3.26e-11	***
## ns(time, 7)3	2.237e-01	3.462e-02	6.462	1.04e-10	***
## ns(time, 7)4	-3.755e-02	3.736e-02	-1.005	0.314840	
## ns(time, 7)5	3.281e-01	2.999e-02	10.942	< 2e-16	***
## ns(time, 7)6	-3.236e-01	6.582e-02	-4.916	8.82e-07	***
## ns(time, 7)7	-3.570e-01	3.210e-02	-11.121	< 2e-16	***
## lon	-8.894e-01	3.739e-03	-237.849	< 2e-16	***
## lat	4.924e-01	3.850e-03	127.907	< 2e-16	***

Signif. codes: 0 '***' 0.001 '**' 0.01 '*' 0.05 '.' 0.1 ' ' 1

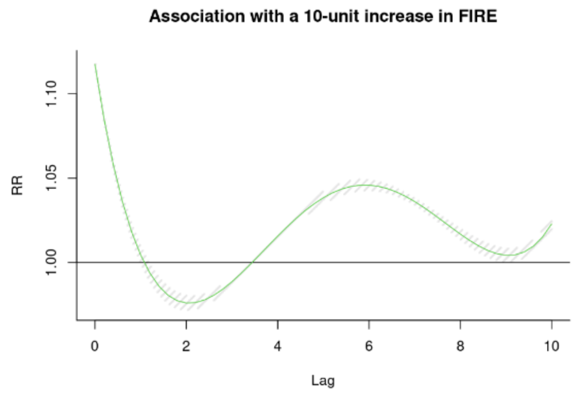

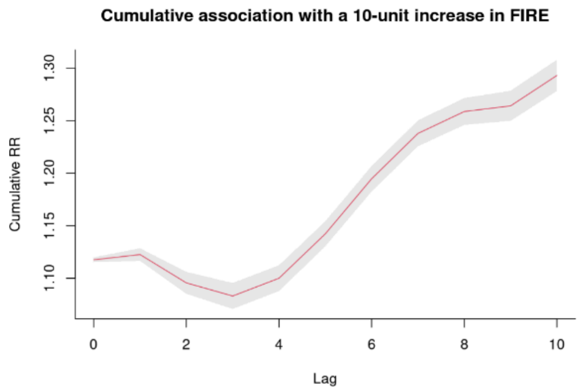

Statistical analysis

Table S7.

lag 0	lag 0.2	lag 0.4	lag 0.6	lag 0.8	lag 1	lag 1.2	lag 1.4
1.1153375	1.0827163	1.0549794	1.0322182	1.0139517	0.9996331	0.9887626	0.9808979

lag 1.6	lag 1.8	lag 2	lag 2.2	lag 2.4	lag 2.6	lag 2.8	lag 3
0.9756478	0.9726631	0.9716288	0.9722570	0.9742806	0.9774474	0.9815144	0.9862433

lag 3.2	lag 3.4	lag 3.6	lag 3.8	lag 4	lag 4.2	lag 4.4	lag 4.6
0.9914020	0.9967826	1.0022287	1.0076353	1.0129199	1.0180012	1.0227959	1.0272224

lag 4.8	lag 5	lag 5.2	lag 5.4	lag 5.6	lag 5.8	lag 6	lag 6.2
1.0312047	1.0346756	1.0375777	1.0398656	1.0415053	1.0424755	1.0427665	1.0423792

lag 6.4	lag 6.6	lag 6.8	lag 7	lag 7.2	lag 7.4	lag 7.6	lag 7.8
1.0413225	1.0396093	1.0372523	1.0342684	1.0307082	1.0266889	1.0223826	1.0179789

lag 8	lag 8.2	lag 8.4	lag 8.6	lag 8.8	lag 9	lag 9.2	lag 9.4
1.0136672	1.0096374	1.0060829	1.0032028	1.0012026	1.0002912	1.0006739	1.0025357

lag 9.6	lag 9.8	lag 10	lag 0	lag 0.2	lag 0.4	lag 0.6	lag 0.8
1.0060116	1.0111748	1.0181168	1.1198060	1.0872473	1.0612025	1.0400430	1.0229526

lag 1	lag 1.2	lag 1.4	lag 1.6	lag 1.8	lag 2	lag 2.2	lag 2.4
1.0093729	0.9988526	0.9910069	0.9854999	0.9820347	0.9803458	0.9801944	0.9813648

lag 2.6	lag 2.8	lag 3	lag 3.2	lag 3.4	lag 3.6	lag 3.8	lag 4
0.9836633	0.9869182	0.9909802	0.9957180	1.0009983	1.0066577	1.0125028	1.0183388

lag 4.2	lag 4.4	lag 4.6	lag 4.8	lag 5	lag 5.2	lag 5.4	lag 5.6
1.0239922	1.0293146	1.0341806	1.0384852	1.0421424	1.0450845	1.0472625	1.0486463

lag 5.8	lag 6	lag 6.2	lag 6.4	lag 6.6	lag 6.8	lag 7	lag 7.2
1.0492254	1.0490093	1.0480293	1.0463404	1.0440252	1.0411969	1.0379941	1.0345505

lag 7.4	lag 7.6	lag 7.8	lag 8	lag 8.2	lag 8.4	lag 8.6	lag 8.8
1.0309600	1.0272877	1.0236064	1.0200129	1.0166276	1.0135906	1.0110607	1.0092165

lag 9	lag 9.2	lag 9.4	lag 9.6	lag 9.8	lag 10
1.0082605	1.0084308	1.0100228	1.0134239	1.0191359	1.0277044

Statistical analysis

Distributed Lag Non-linear Models

Impact of emissions from forest fires on hospitalizations for cardiovascular diseases

With Control of Seasonality Bias - Cardiovascular Diseases (5 years analisys)

Table S8.

pred1.fire$allRRfit["10"]	1.2930480
cbind(pred1.fire$allRRlow, pred1.fire$allRRhigh)["10",]	1.278370 1.307895

Table S9.

pred1.fire$cumRRlow["10",]

lag 0	lag 1	lag 2	lag 3	lag 4	lag 5	lag 6	lag 7	lag 8	lag 9	lag 10
1.1153370	1.1167500	1.0853720	1.0709840	1.0879840	1.1306500	1.1828410	1.2257410	1.2461480	1.2499700	1.2783700

pred1.fire$cumRRhigh["10",]

lag 0	lag 1	lag 2	lag 3	lag 4	lag 5	lag 6	lag 7	lag 8	lag 9	lag 10
1.1198060	1.1284580	1.1059680	1.0954360	1.1122820	1.1540930	1.2067260	1.2501570	1.2714370	1.2783910	1.3078950

Statistical analysis

Distributed Lag Non-linear Models

Impact of emissions from forest fires on hospitalizations for cardiovascular diseases

With Control of Seasonality Bias - Cardiovascular Diseases (10 years analisys)

Table S10.

Coefficients:

##	Estimate	Std. Error	z value	Pr(>|z|)	
## (Intercept)	-4.805e+01	1.671e-01	-287.635	< 2e-16	***
## cb1.firev1.l1	7.728e-03	1.397e-04	55.303	< 2e-16	***
## cb1.firev1.l2	-9.135e-02	3.934e-03	-23.217	< 2e-16	***
## cb1.firev1.l3	3.041e-01	1.895e-02	16.051	< 2e-16	***
## cb1.firev1.l4	-3.605e-01	2.939e-02	-12.263	< 2e-16	***
## cb1.firev1.l5	1.410e-01	1.443e-02	9.769	< 2e-16	***
## cb1.pm10v1.l1	2.047e-01	7.162e-03	28.583	< 2e-16	***
## cb1.pm10v1.l2	-4.469e+00	1.349e-01	-33.120	< 2e-16	***
## cb1.pm10v1.l3	1.898e+01	5.982e-01	31.730	< 2e-16	***
## cb1.pm10v1.l4	-2.719e+01	9.367e-01	-29.029	< 2e-16	***
## cb1.pm10v1.l5	1.245e+01	4.735e-01	26.286	< 2e-16	***
## cb1.pm25v1.l1	-6.250e-01	2.859e-02	-21.859	< 2e-16	***
## cb1.pm25v1.l2	1.250e+01	5.383e-01	23.224	< 2e-16	***
## cb1.pm25v1.l3	-5.134e+01	2.386e+00	-21.515	< 2e-16	***
## cb1.pm25v1.l4	7.150e+01	3.734e+00	19.149	< 2e-16	***
## cb1.pm25v1.l5	-3.182e+01	1.886e+00	-16.875	< 2e-16	***
## cb1.so2v1.l1	-3.855e-01	2.611e-02	-14.765	< 2e-16	***
## cb1.so2v1.l2	-6.481e+00	4.936e-01	-13.131	< 2e-16	***
## cb1.so2v1.l3	5.246e+01	2.144e+00	24.464	< 2e-16	***
## cb1.so2v1.l4	-9.558e+01	3.328e+00	-28.717	< 2e-16	***
## cb1.so2v1.l5	5.018e+01	1.679e+00	29.880	< 2e-16	***
## cb1.no2v1.l1	-8.923e-01	5.822e-02	-15.325	< 2e-16	***
## cb1.no2v1.l2	9.199e+00	9.476e-01	9.708	< 2e-16	***
## cb1.no2v1.l3	-3.575e+01	3.996e+00	-8.946	< 2e-16	***
## cb1.no2v1.l4	5.403e+01	6.010e+00	8.989	< 2e-16	***
## cb1.no2v1.l5	-2.679e+01	2.944e+00	-9.099	< 2e-16	***
## cb1.t2mv1.l1	-1.225e+01	2.984e-01	-41.056	< 2e-16	***
## cb1.t2mv1.l2	1.639e+01	4.034e-01	40.630	< 2e-16	***
## cb1.t2mv2.l1	-9.617e+00	3.000e-01	-32.051	< 2e-16	***
## cb1.t2mv2.l2	1.291e+01	4.057e-01	31.817	< 2e-16	***
## cb1.t2mv3.l1	-1.624e+00	2.041e-01	-7.957	1.76e-15	***
## cb1.t2mv3.l2	2.078e+00	2.743e-01	7.576	3.55e-14	***
## cb1.t2mv4.l1	-2.250e+01	6.075e-01	-37.044	< 2e-16	***
## cb1.t2mv4.l2	2.982e+01	8.209e-01	36.327	< 2e-16	***
## cb1.t2mv5.l1	-3.799e+00	3.473e-01	-10.939	< 2e-16	***
## cb1.t2mv5.l2	4.602e+00	4.626e-01	9.948	< 2e-16	***
## rh2m	-8.347e-04	3.077e-04	-2.713	0.006676	**
## seasonAutumn	3.894e-02	9.405e-03	4.140	3.47e-05	***
## seasonSpring	1.906e-01	1.045e-02	18.238	< 2e-16	***
## seasonWinter	4.382e-02	1.289e-02	3.400	0.000673	***
## Signif. codes: 0 '***' 0.001 '**' 0.01 '*' 0.05 '.' 0.1 ' ' 1					
## ns(time, 7)1	-1.223e-01	2.044e-02	-5.981	2.21e-09	***
## ns(time, 7)2	-2.501e-01	2.726e-02	-9.177	< 2e-16	***
## ns(time, 7)3	-4.685e-03	2.389e-02	-0.196	0.844553	
## ns(time, 7)4	-2.326e-01	2.516e-02	-9.243	< 2e-16	***
## ns(time, 7)5	1.458e-01	2.060e-02	7.080	1.44e-12	***
## ns(time, 7)6	-1.400e-01	4.146e-02	-3.377	0.000732	***
## ns(time, 7)7	-3.039e-01	1.996e-02	-15.226	< 2e-16	***
## lon	-9.480e-01	2.484e-03	-381.725	< 2e-16	***
## lat	5.493e-01	2.603e-03	211.014	< 2e-16	***

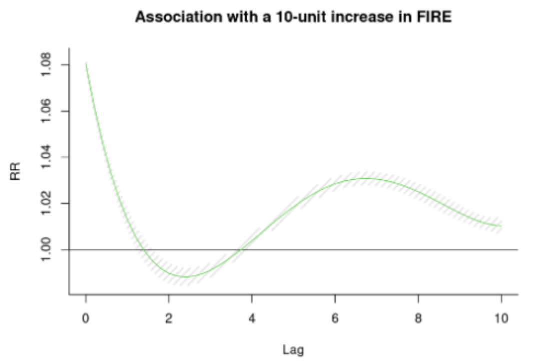

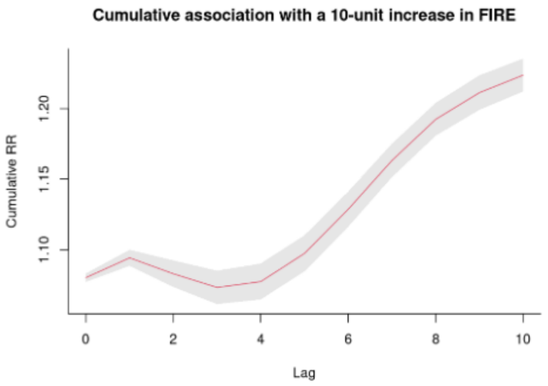

Statistical analysis

Distributed Lag Non-linear Models

Impact of emissions from forest fires on hospitalizations for cardiovascular diseases

With Control of Seasonality Bias - Cardiovascular Diseases (10 years analisys)

Table S11.

lag 0	lag 0.2	lag 0.4	lag 0.6	lag 0.8	lag 1	lag 1.2	lag 1.4
1.0773912	1.0594175	1.0435109	1.0298171	1.0183079	1.0088307	1.0011985	0.9952240

lag 1.6	lag 1.8	lag 2	lag 2.2	lag 2.4	lag 2.6	lag 2.8	lag 3
0.9907292	0.9875480	0.9855258	0.9845177	0.9843883	0.9850097	0.9862612	0.9880285

lag 3.2	lag 3.4	lag 3.6	lag 3.8	lag 4	lag 4.2	lag 4.4	lag 4.6
0.9902051	0.9926929	0.9954050	0.9982669	1.0012159	1.0041988	1.0071682	1.0100807

lag 4.8	lag 5	lag 5.2	lag 5.4	lag 5.6	lag 5.8	lag 6	lag 6.2
1.0128948	1.0155711	1.0180716	1.0203609	1.0224054	1.0241746	1.0256404	1.0267784

lag 6.4	lag 6.6	lag 6.8	lag 7	lag 7.2	lag 7.4	lag 7.6	lag 7.8
1.0275683	1.0279956	1.0280536	1.0277460	1.0270878	1.0261063	1.0248395	1.0233344

lag 8	lag 8.2	lag 8.4	lag 8.6	lag 8.8	lag 9	lag 9.2	lag 9.4
1.0216453	1.0198320	1.0179588	1.0160928	1.0143020	1.0126502	1.0111872	1.0099266

lag 9.6	lag 9.8	lag 10	lag 0	lag 0.2	lag 0.4	lag 0.6	lag 0.8
1.0088125	1.0077244	1.0066070	1.0833092	1.0646867	1.0493508	1.0365939	1.0259459

lag 1	lag 1.2	lag 1.4	lag 1.6	lag 1.8	lag 2	lag 2.2	lag 2.4
1.0171152	1.0098897	1.0040966	0.9995863	0.9962255	0.9938937	0.9924811	0.9918867

lag 2.6	lag 2.8	lag 3	lag 3.2	lag 3.4	lag 3.6	lag 3.8	lag 4
0.9920178	0.9927884	0.9941186	0.9959326	0.9981561	1.0007142	1.0035291	1.0065205

lag 4.2	lag 4.4	lag 4.6	lag 4.8	lag 5	lag 5.2	lag 5.4	lag 5.6
1.0096081	1.0127148	1.0157692	1.0187077	1.0214746	1.0240225	1.0263126	1.0283145

lag 5.8	lag 6	lag 6.2	lag 6.4	lag 6.6	lag 6.8	lag 7	lag 7.2
1.0300061	1.0313739	1.0324125	1.0331239	1.0335160	1.0336008	1.0333920	1.0329031

lag 7.4	lag 7.6	lag 7.8	lag 8	lag 8.2	lag 8.4	lag 8.6	lag 8.8
1.0321474	1.0311391	1.0298943	1.0284333	1.0267816	1.0249708	1.0230403	1.0210389

lag 9	lag 9.2	lag 9.4	lag 9.6	lag 9.8	lag 10
1.0190297	1.0171004	1.0153847	1.0140969	1.0135264	1.0139093

Statistical analysis

Distributed Lag Non-linear Models

Impact of emissions from forest fires on hospitalizations for cardiovascular diseases

With Control of Seasonality Bias - Cardiovascular Diseases (10 years analisys)

Table S12.

pred1.fire$allRRfit["10"]	1.223717
cbind(pred1.fire$allRRlow, pred1.fire$allRRhigh)["10",]	1.212189 1.235355

Table S13.

pred1.fire$cumRRlow["10",]

lag 0	lag 1	lag 2	lag 3	lag 4	lag 5	lag 6	lag 7	lag 8	lag 9	lag 10
1.0773910	1.0887110	1.0737490	1.0616980	1.0649520	1.0848530	1.1164750	1.1515640	1.1808870	1.1991930	1.2121890

pred1.fire$cumRRhigh["10",]

lag 0	lag 1	lag 2	lag 3	lag 4	lag 5	lag 6	lag 7	lag 8	lag 9	lag 10
1.0833090	1.1000230	1.0924950	1.0852480	1.0903110	1.1103180	1.1412490	1.1751470	1.2040600	1.2235280	1.2353550

Statistical analysis

Distributed Lag Non-linear Models

Impact of emissions from forest fires on hospitalizations for respiratory diseases

With Control of Seasonality Bias - Respiratory Diseases (5 years analisys)

Table S14.

Coefficients:

##	Estimate	Std. Error	z value	Pr(>|z|)	
## (Intercept)	-3.429e+01	2.203e-01	-155.658	< 2e-16	***
## cb1.firev1.l1	1.194e-02	7.782e-05	153.364	< 2e-16	***
## cb1.firev1.l2	-2.157e-01	2.423e-03	-89.012	< 2e-16	***
## cb1.firev1.l3	8.947e-01	1.186e-02	75.415	< 2e-16	***
## cb1.firev1.l4	-1.277e+00	1.826e-02	-69.967	< 2e-16	***
## cb1.firev1.l5	5.936e-01	8.832e-03	67.212	< 2e-16	***
## cb1.pm10v1.l1	8.260e-02	8.907e-03	9.273	< 2e-16	***
## cb1.pm10v1.l2	-2.710e+00	1.593e-01	-17.017	< 2e-16	***
## cb1.pm10v1.l3	1.535e+01	6.677e-01	22.988	< 2e-16	***
## cb1.pm10v1.l4	-2.694e+01	1.015e+00	-26.537	< 2e-16	***
## cb1.pm10v1.l5	1.438e+01	5.067e-01	28.370	< 2e-16	***
## cb1.pm25v1.l1	-1.774e-01	3.545e-02	-5.005	5.59e-07	***
## cb1.pm25v1.l2	5.574e+00	6.306e-01	8.839	< 2e-16	***
## cb1.pm25v1.l3	-3.531e+01	2.641e+00	-13.372	< 2e-16	***
## cb1.pm25v1.l4	6.617e+01	4.019e+00	16.463	< 2e-16	***
## cb1.pm25v1.l5	-3.672e+01	2.009e+00	-18.276	< 2e-16	***
## cb1.so2v1.l3	-1.504e+02	1.375e+00	-109.442	< 2e-16	***
## cb1.so2v1.l4	2.191e+02	2.178e+00	100.603	< 2e-16	***
## cb1.so2v1.l5	-1.052e+02	1.113e+00	-94.493	< 2e-16	***
## cb1.no2v1.l1	-4.516e-02	6.015e-02	-0.751	0.452726	
## cb1.no2v1.l2	6.503e+00	8.728e-01	7.451	9.23e-14	***
## cb1.no2v1.l3	-4.355e+01	3.605e+00	-12.081	< 2e-16	***
## cb1.no2v1.l4	8.372e+01	5.329e+00	15.710	< 2e-16	***
## cb1.no2v1.l5	-4.795e+01	2.559e+00	-18.737	< 2e-16	***
## cb1.t2mv1.l1	-9.661e+00	2.371e-01	-40.743	< 2e-16	***
## cb1.t2mv1.l2	1.297e+01	3.198e-01	40.540	< 2e-16	***
## cb1.t2mv2.l1	-5.859e+00	2.403e-01	-24.385	< 2e-16	***
## cb1.t2mv2.l2	7.954e+00	3.239e-01	24.558	< 2e-16	***
## cb1.t2mv3.l1	-1.145e+00	1.817e-01	-6.303	2.92e-10	***
## cb1.t2mv3.l2	1.418e+00	2.430e-01	5.836	5.35e-09	***
## cb1.t2mv4.l1	-1.400e+01	4.899e-01	-28.587	< 2e-16	***
## cb1.t2mv5.l2	-1.980e+00	3.431e-01	-5.772	7.85e-09	***
## rh2m	-5.589e-03	3.918e-04	-14.262	< 2e-16	***
## seasonAutumn	4.823e-01	1.189e-02	40.573	< 2e-16	***
## seasonSpring	3.519e-01	1.400e-02	25.137	< 2e-16	***
## seasonWinter	2.507e-01	1.706e-02	14.692	< 2e-16	***
## ns(time, 7)1	2.405e-01	2.615e-02	9.195	< 2e-16	***
## ns(time, 7)2	1.492e-01	3.503e-02	4.259	2.05e-05	***
## ns(time, 7)3	7.991e-02	3.166e-02	2.524	0.011607	*
## ns(time, 7)4	3.210e-02	3.361e-02	0.955	0.339575	
## ns(time, 7)5	2.743e-01	2.673e-02	10.261	< 2e-16	***
## ns(time, 7)6	-1.929e-01	6.099e-02	-3.164	0.001558	**
## ns(time, 7)7	-5.059e-01	3.117e-02	-16.231	< 2e-16	***
## lon	-5.583e-01	4.295e-03	-129.999	< 2e-16	***
## lat	8.061e-02	4.525e-03	17.814	< 2e-16	***

Signif. codes: 0 '***' 0.001 '**' 0.01 '*' 0.05 '.' 0.1 ' ' 1

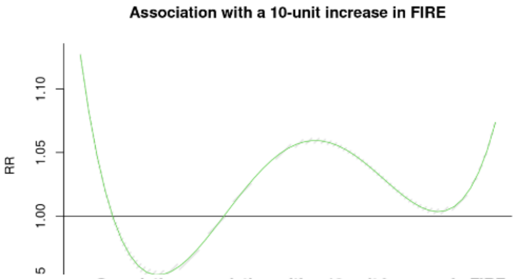

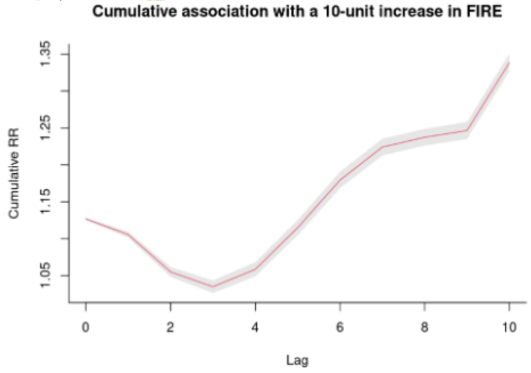

Statistical analysis

Distributed Lag Non-linear Models

Impact of emissions from forest fires on hospitalizations for respiratory diseases

With Control of Seasonality Bias - Respiratory Diseases (5 years analisys)

Table S15.

lag 0	lag 0.2	lag 0.4	lag 0.6	lag 0.8	lag 1	lag 1.2	lag 1.4
1.1250484	1.0812441	1.0456830	1.0173208	0.9951676	0.9783495	0.9661185	0.9578348

lag 1.6	lag 1.8	lag 2	lag 2.2	lag 2.4	lag 2.6	lag 2.8	lag 3
0.9529469	0.9509722	0.9514827	0.9540929	0.9584505	0.9642295	0.9711252	0.9788509

lag 3.2	lag 3.4	lag 3.6	lag 3.8	lag 4	lag 4.2	lag 4.4	lag 4.6
0.9871362	0.9957270	1.0043867	1.0128985	1.0210662	1.0287155	1.0356927	1.0418655

lag 4.8	lag 5	lag 5.2	lag 5.4	lag 5.6	lag 5.8	lag 6	lag 6.2
1.0471227	1.0513755	1.0545583	1.0566304	1.0575762	1.0574061	1.0561558	1.0538862

lag 6.4	lag 6.6	lag 6.8	lag 7	lag 7.2	lag 7.4	lag 7.6	lag 7.8
1.0506823	1.0466521	1.0419256	1.0366562	1.0310221	1.0252292	1.0195102	1.0141215

lag 8	lag 8.2	lag 8.4	lag 8.6	lag 8.8	lag 9	lag 9.2	lag 9.4
1.0093391	1.0054572	1.0027884	1.0016664	1.0024492	1.0055236	1.0113095	1.0202627

lag 9.6	lag 9.8	lag 10	lag 0	lag 0.2	lag 0.4	lag 0.6	lag 0.8
1.0328771	1.0496960	1.0713578	1.1284856	1.0846724	1.0497192	1.0220192	1.0004013

lag 1	lag 1.2	lag 1.4	lag 1.6	lag 1.8	lag 2	lag 2.2	lag 2.4
0.9839577	0.9719481	0.9637517	0.9588371	0.9567418	0.9570568	0.9594151	0.9634827

lag 2.6	lag 2.8	lag 3	lag 3.2	lag 3.4	lag 3.6	lag 3.8	lag 4
0.9689526	0.9755396	0.9829768	0.9910132	0.9994113	1.0079453	1.0164002	1.0245720

lag 4.2	lag 4.4	lag 4.6	lag 4.8	lag 5	lag 5.2	lag 5.4	lag 5.6
1.0322701	1.0393210	1.0455717	1.0508930	1.0551819	1.0583628	1.0603891	1.0612438

lag 5.8	lag 6	lag 6.2	lag 6.4	lag 6.6	lag 6.8	lag 7	lag 7.2
1.0609398	1.0595199	1.0570566	1.0536516	1.0494344	1.0445607	1.0392085	1.0335731

lag 7.4	lag 7.6	lag 7.8	lag 8	lag 8.2	lag 8.4	lag 8.6	lag 8.8
1.0278624	1.0222949	1.0171020	1.0125320	1.0088526	1.0063540	1.0053522	1.0061934

lag 9	lag 9.2	lag 9.4	lag 9.6	lag 9.8	lag 10
1.0092621	1.0149929	1.0238883	1.0365451	1.0536786	1.0761238

Statistical analysis

Distributed Lag Non-linear Models

Impact of emissions from forest fires on hospitalizations for respiratory diseases

With Control of Seasonality Bias - Respiratory Diseases (5 years analisys)

Table S16.

pred1.fire$allRRfit["10"]	1.338491
cbind(pred1.fire$allRRlow, pred1.fire$allRRhigh)["10",]	1.32653 1.35056

Table S17.

pred1.fire$cumRRlow["10",]

lag 0	lag 1	lag 2	lag 3	lag 4	lag 5	lag 6	lag 7	lag 8	lag 9	lag 10
1.1250480	1.1015280	1.0483220	1.0264000	1.0489060	1.1044800	1.1683200	1.2127720	1.2261440	1.2350870	1.3265300

pred1.fire$cumRRhigh["10",]

lag 0	lag 1	lag 2	lag 3	lag 4	lag 5	lag 6	lag 7	lag 8	lag 9	lag 10
1.1284860	1.1095380	1.0616510	1.0433250	1.0680610	1.1252780	1.1904000	1.2354160	1.2488110	1.2581630	1.3505600

Statistical analysis

Distributed Lag Non-linear Models

Impact of emissions from forest fires on hospitalizations for respiratory diseases

With Control of Seasonality Bias - Respiratory Diseases (10 years analisys)

Table S18.

Coefficients:

<table>
<tr><th>## Estimate</th><th>Std. Error</th><th></th><th>z value</th><th>Pr(>|z|)</th></tr>
<tr><td>## (Intercept)</td><td>-3.902e+01</td><td>1.489e-01</td><td>-262.057</td><td>< 2e-16 ***</td></tr>
<tr><td>## cb1.firev1.l1</td><td>1.025e-02</td><td>4.933e-05</td><td>207.710</td><td>< 2e-16 ***</td></tr>
<tr><td>## cb1.firev1.l2</td><td>-1.511e-01</td><td>2.175e-03</td><td>-69.456</td><td>< 2e-16 ***</td></tr>
<tr><td>## cb1.firev1.l3</td><td>5.979e-01</td><td>1.101e-02</td><td>54.318</td><td>< 2e-16 ***</td></tr>
<tr><td>## cb1.firev1.l4</td><td>-8.358e-01</td><td>1.755e-02</td><td>-47.624</td><td>< 2e-16 ***</td></tr>
<tr><td>## cb1.firev1.l5</td><td>3.811e-01</td><td>8.746e-03</td><td>43.573</td><td>< 2e-16 ***</td></tr>
<tr><td>## cb1.pm10v1.l1</td><td>6.013e-02</td><td>7.191e-03</td><td>8.361</td><td>< 2e-16 ***</td></tr>
<tr><td>## cb1.pm10v1.l2</td><td>-2.837e+00</td><td>1.289e-01</td><td>-22.004</td><td>< 2e-16 ***</td></tr>
<tr><td>## cb1.pm10v1.l3</td><td>1.534e+01</td><td>5.523e-01</td><td>27.781</td><td>< 2e-16 ***</td></tr>
<tr><td>## cb1.pm10v1.l4</td><td>-2.534e+01</td><td>8.554e-01</td><td>-29.619</td><td>< 2e-16 ***</td></tr>
<tr><td>## cb1.pm10v1.l5</td><td>1.285e+01</td><td>4.323e-01</td><td>29.725</td><td>< 2e-16 ***</td></tr>
<tr><td>## cb1.pm25v1.l1</td><td>-7.629e-02</td><td>2.869e-02</td><td>-2.659</td><td>0.007835 **</td></tr>
<tr><td>## cb1.pm25v1.l2</td><td>6.097e+00</td><td>5.150e-01</td><td>11.839</td><td>< 2e-16 ***</td></tr>
<tr><td>## cb1.pm25v1.l3</td><td>-3.668e+01</td><td>2.218e+00</td><td>-16.540</td><td>< 2e-16 ***</td></tr>
<tr><td>## cb1.pm25v1.l4</td><td>6.355e+01</td><td>3.449e+00</td><td>18.424</td><td>< 2e-16 ***</td></tr>
</table>

Coefficients:

<table>
<tr><th>## Estimate</th><th>Std. Error</th><th></th><th>z value</th><th>Pr(>|z|)</th></tr>
<tr><td>## cb1.pm25v1.l5</td><td>-3.310e+01</td><td>1.747e+00</td><td>-18.942</td><td>< 2e-16 ***</td></tr>
<tr><td>## cb1.so2v1.l1</td><td>-6.477e-01</td><td>1.799e-02</td><td>-36.010</td><td>< 2e-16 ***</td></tr>
<tr><td>## cb1.so2v1.l2</td><td>-1.608e-01</td><td>3.585e-01</td><td>-0.448</td><td>0.653900</td></tr>
<tr><td>## cb1.so2v1.l3</td><td>2.563e+01</td><td>1.622e+00</td><td>15.803</td><td>< 2e-16 ***</td></tr>
<tr><td>## cb1.so2v1.l4</td><td>-5.494e+01</td><td>2.562e+00</td><td>-21.447</td><td>< 2e-16 ***</td></tr>
<tr><td>## cb1.so2v1.l5</td><td>2.981e+01</td><td>1.297e+00</td><td>22.972</td><td>< 2e-16 ***</td></tr>
<tr><td>## cb1.no2v1.l1</td><td>-3.290e-01</td><td>3.683e-02</td><td>-8.933</td><td>< 2e-16 ***</td></tr>
<tr><td>## cb1.no2v1.l2</td><td>2.452e+00</td><td>6.824e-01</td><td>3.593</td><td>0.000327 ***</td></tr>
<tr><td>## cb1.no2v1.l3</td><td>-1.558e+01</td><td>3.057e+00</td><td>-5.096</td><td>3.48e-07 ***</td></tr>
<tr><td>## cb1.no2v1.l4</td><td>3.055e+01</td><td>4.744e+00</td><td>6.439</td><td>1.20e-10 ***</td></tr>
<tr><td>## cb1.no2v1.l5</td><td>-1.710e+01</td><td>2.368e+00</td><td>-7.221</td><td>5.16e-13 ***</td></tr>
<tr><td>## cb1.t2mv1.l1</td><td>-1.040e+01</td><td>2.134e-01</td><td>-48.734</td><td>< 2e-16 ***</td></tr>
<tr><td>## cb1.t2mv1.l2</td><td>1.400e+01</td><td>2.892e-01</td><td>48.407</td><td>< 2e-16 ***</td></tr>
<tr><td>## cb1.t2mv2.l1</td><td>-7.728e+00</td><td>2.143e-01</td><td>-36.068</td><td>< 2e-16 ***</td></tr>
<tr><td>## cb1.t2mv2.l2</td><td>1.045e+01</td><td>2.905e-01</td><td>35.966</td><td>< 2e-16 ***</td></tr>
<tr><td>## cb1.t2mv3.l1</td><td>9.426e-01</td><td>1.573e-01</td><td>5.992</td><td>2.08e-09 ***</td></tr>
<tr><td>## cb1.t2mv3.l2</td><td>-1.291e+00</td><td>2.112e-01</td><td>-6.113</td><td>9.78e-10 ***</td></tr>
<tr><td>## cb1.t2mv4.l1</td><td>-2.298e+01</td><td>4.350e-01</td><td>-52.832</td><td>< 2e-16 ***</td></tr>
</table>

Coefficients:

<table>
<tr><th>## Estimate</th><th>Std. Error</th><th></th><th>z value</th><th>Pr(>|z|)</th></tr>
<tr><td>## cb1.t2mv4.l2</td><td>3.054e+01</td><td>5.890e-01</td><td>51.845</td><td>< 2e-16 ***</td></tr>
<tr><td>## cb1.t2mv5.l1</td><td>-1.050e+01</td><td>2.726e-01</td><td>-38.506</td><td>< 2e-16 ***</td></tr>
<tr><td>## cb1.t2mv5.l2</td><td>1.338e+01</td><td>3.615e-01</td><td>36.999</td><td>< 2e-16 ***</td></tr>
<tr><td>## rh2m</td><td>-1.348e-03</td><td>2.542e-04</td><td>-5.304</td><td>1.13e-07 ***</td></tr>
<tr><td>## seasonAutumn</td><td>4.652e-01</td><td>8.075e-03</td><td>57.615</td><td>< 2e-16 ***</td></tr>
<tr><td>## seasonSpring</td><td>3.525e-01</td><td>9.403e-03</td><td>37.487</td><td>< 2e-16 ***</td></tr>
<tr><td>## seasonWinter</td><td>2.962e-01</td><td>1.100e-02</td><td>26.935</td><td>< 2e-16 ***</td></tr>
<tr><td>## ns(time, 7)1</td><td>-4.409e-01</td><td>1.665e-02</td><td>-26.473</td><td>< 2e-16 ***</td></tr>
<tr><td>## ns(time, 7)2</td><td>-4.337e-01</td><td>2.222e-02</td><td>-19.521</td><td>< 2e-16 ***</td></tr>
<tr><td>## ns(time, 7)3</td><td>-3.825e-01</td><td>1.954e-02</td><td>-19.573</td><td>< 2e-16 ***</td></tr>
<tr><td>## ns(time, 7)4</td><td>-2.904e-01</td><td>2.046e-02</td><td>-14.195</td><td>< 2e-16 ***</td></tr>
<tr><td>## ns(time, 7)5</td><td>-3.045e-01</td><td>1.768e-02</td><td>-17.220</td><td>< 2e-16 ***</td></tr>
<tr><td>## ns(time, 7)6</td><td>-6.690e-01</td><td>3.301e-02</td><td>-20.269</td><td>< 2e-16 ***</td></tr>
<tr><td>## ns(time, 7)7</td><td>-3.413e-01</td><td>1.714e-02</td><td>-19.914</td><td>< 2e-16 ***</td></tr>
<tr><td>## lon</td><td>-6.901e-01</td><td>2.699e-03</td><td>-255.728</td><td>< 2e-16 ***</td></tr>
<tr><td>## lat</td><td>2.239e-01</td><td>2.865e-03</td><td>78.160</td><td>< 2e-16 ***</td></tr>
<tr><td>## ---</td><td></td><td></td><td></td><td></td></tr>
<tr><td colspan="5">## Signif. codes: 0 '***' 0.001 '**' 0.01 '*' 0.05 '.' 0.1 ' ' 1</td></tr>
</table>

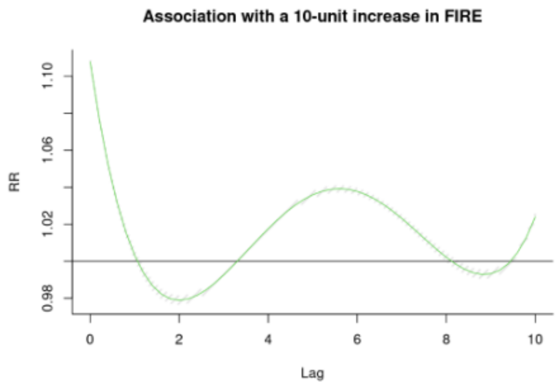

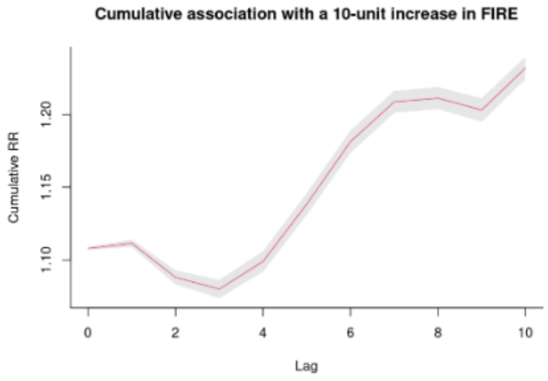

Statistical analysis

Distributed Lag Non-linear Models

Impact of emissions from forest fires on hospitalizations for respiratory diseases

With Control of Seasonality Bias - Respiratory Diseases (10 years analisys)

Table S19.

lag 0	lag 0.2	lag 0.4	lag 0.6	lag 0.8	lag 1	lag 1.2	lag 1.4
1.1068189	1.0764157	1.0510393	1.0303592	1.0138497	1.0009868	0.9913009	0.9843778

lag 1.6	lag 1.8	lag 2	lag 2.2	lag 2.4	lag 2.6	lag 2.8	lag 3
0.9798498	0.9773878	0.9766943	0.9774976	0.9795471	0.9826100	0.9864690	0.9909211

lag 3.2	lag 3.4	lag 3.6	lag 3.8	lag 4	lag 4.2	lag 4.4	lag 4.6
0.9957777	1.0008654	1.0060271	1.0111220	1.0160248	1.0206236	1.0248192	1.0285248

lag 4.8	lag 5	lag 5.2	lag 5.4	lag 5.6	lag 5.8	lag 6	lag 6.2
1.0316662	1.0341819	1.0360246	1.0371607	1.0375714	1.0372520	1.0362125	1.0344773

lag 6.4	lag 6.6	lag 6.8	lag 7	lag 7.2	lag 7.4	lag 7.6	lag 7.8
1.0320858	1.0290941	1.0255768	1.0216291	1.0173661	1.0129211	1.0084421	1.0040902

lag 8	lag 8.2	lag 8.4	lag 8.6	lag 8.8	lag 9	lag 9.2	lag 9.4
1.0000385	0.9964713	0.9935849	0.9915883	0.9907035	0.9911670	0.9932281	0.9971436

lag 9.6	lag 9.8	lag 10	lag 0	lag 0.2	lag 0.4	lag 0.6	lag 0.8
1.0031568	1.0114590	1.0222339	1.1089611	1.0784172	1.0537454	1.0338042	1.0178725

lag 1	lag 1.2	lag 1.4	lag 1.6	lag 1.8	lag 2	lag 2.2	lag 2.4
1.0054083	0.9959592	0.9891347	0.9845913	0.9820233	0.9811551	0.9817368	0.9835390

lag 2.6	lag 2.8	lag 3	lag 3.2	lag 3.4	lag 3.6	lag 3.8	lag 4
0.9863507	0.9899758	0.9942313	0.9989446	1.0039517	1.0090951	1.0142244	1.0191979

lag 4.2	lag 4.4	lag 4.6	lag 4.8	lag 5	lag 5.2	lag 5.4	lag 5.6
1.0238847	1.0281678	1.0319452	1.0351315	1.0376584	1.0394754	1.0405503	1.0408697

lag 5.8	lag 6	lag 6.2	lag 6.4	lag 6.6	lag 6.8	lag 7	lag 7.2
1.0404391	1.0392832	1.0374455	1.0349869	1.0319838	1.0285242	1.0247054	1.0206327

lag 7.4	lag 7.6	lag 7.8	lag 8	lag 8.2	lag 8.4	lag 8.6	lag 8.8
1.0164202	1.0121928	1.0080871	1.0042526	1.0008515	0.9980593	0.9960661	0.9950777

lag 9	lag 9.2	lag 9.4	lag 9.6	lag 9.8	lag 10
0.9953195	0.9970423	1.0005357	1.0061583	1.0143880	1.0257932

Statistical analysis

Distributed Lag Non-linear Models

Impact of emissions from forest fires on hospitalizations for respiratory diseases

With Control of Seasonality Bias - Respiratory Diseases (10 years analisys)

Table S20.

pred1.fire$allRRfit["10"]	1.232047
cbind(pred1.fire$allRRlow, pred1.fire$allRRhigh)["10",]	1.224355 1.239788

Table S21.

pred1.fire$cumRRlow["10",]

lag 0	lag 1	lag 2	lag 3	lag 4	lag 5	lag 6	lag 7	lag 8	lag 9	lag 10
1.1068190	1.1088430	1.0831950	1.0736910	1.0919650	1.1309920	1.1737500	1.2012460	1.2038570	1.1951770	1.2243550

pred1.fire$cumRRhigh["10",]

lag 0	lag 1	lag 2	lag 3	lag 4	lag 5	lag 6	lag 7	lag 8	lag 9	lag 10
1.1089610	1.1140210	1.0928310	1.0861940	1.1059630	1.1458850	1.1890710	1.2163080	1.2188790	1.2111910	1.2397880

Statistical analysis

Distributed Lag Non-linear Models

Impact of emissions from forest fires on hospitalizations for respiratory diseases

Without Control of Seasonality Bias - Respiratory Diseases (5 years analisys)

Table S22.

Coefficients:				
**Estimate**	**Std.**	**Error**	**z value**	**Pr(>|z|)**
(Intercept)	-3.430e+01	2.187e-01	-156.831	< 2e-16 ***
cb1.firev1.l1	1.189e-02	7.788e-05	152.638	< 2e-16 ***
cb1.firev1.l2	-2.156e-01	2.492e-03	-86.490	< 2e-16 ***
cb1.firev1.l3	8.952e-01	1.218e-02	73.523	< 2e-16 ***
cb1.firev1.l4	-1.278e+00	1.869e-02	-68.384	< 2e-16 ***
cb1.firev1.l5	5.940e-01	9.028e-03	65.794	< 2e-16 ***
cb1.pm10v1.l1	7.960e-02	8.790e-03	9.056	< 2e-16 ***
cb1.pm10v1.l2	-2.586e+00	1.576e-01	-16.410	< 2e-16 ***
cb1.pm10v1.l3	1.466e+01	6.629e-01	22.117	< 2e-16 ***
cb1.pm10v1.l4	-2.578e+01	1.010e+00	-25.516	< 2e-16 ***
cb1.pm10v1.l5	1.378e+01	5.051e-01	27.278	< 2e-16 ***
cb1.pm25v1.l1	-1.739e-01	3.498e-02	-4.972	6.63e-07 ***
cb1.pm25v1.l2	5.244e+00	6.238e-01	8.407	< 2e-16 ***
cb1.pm25v1.l3	-3.329e+01	2.622e+00	-12.694	< 2e-16 ***
cb1.pm25v1.l4	6.260e+01	4.002e+00	15.644	< 2e-16 ***
cb1.pm25v1.l5	-3.484e+01	2.004e+00	-17.387	< 2e-16 ***
cb1.so2v1.l1	-2.020e+00	1.976e-02	-102.211	< 2e-16 ***
cb1.so2v1.l2	3.693e+01	3.107e-01	118.842	< 2e-16 ***
cb1.so2v1.l3	-1.488e+02	1.387e+00	-107.309	< 2e-16 ***
cb1.so2v1.l4	2.166e+02	2.203e+00	98.318	< 2e-16 ***
cb1.so2v1.l5	-1.039e+02	1.128e+00	-92.156	< 2e-16 ***
cb1.no2v1.l1	-5.552e-03	5.886e-02	-0.094	0.924851
cb1.no2v1.l2	5.870e+00	8.614e-01	6.815	9.44e-12 ***
cb1.no2v1.l3	-4.093e+01	3.578e+00	-11.437	< 2e-16 ***
cb1.no2v1.l4	7.975e+01	5.305e+00	15.033	< 2e-16 ***
cb1.no2v1.l5	-4.597e+01	2.551e+00	-18.017	< 2e-16 ***
cb1.t2mv1.l1	-9.571e+00	2.355e-01	-40.639	< 2e-16 ***
cb1.t2mv1.l2	1.288e+01	3.177e-01	40.531	< 2e-16 ***
cb1.t2mv2.l1	-5.752e+00	2.384e-01	-24.125	< 2e-16 ***
cb1.t2mv2.l2	7.841e+00	3.215e-01	24.386	< 2e-16 ***
cb1.t2mv3.l1	-1.128e+00	1.809e-01	-6.235	4.51e-10 ***
cb1.t2mv3.l2	1.410e+00	2.422e-01	5.823	5.78e-09 ***
cb1.t2mv4.l1	-1.385e+01	4.864e-01	-28.467	< 2e-16 ***
cb1.t2mv4.l2	1.839e+01	6.539e-01	28.127	< 2e-16 ***
cb1.t2mv5.l1	8.488e-01	2.650e-01	3.203	0.001361 **
cb1.t2mv5.l2	-1.938e+00	3.452e-01	-5.615	1.97e-08 ***

Table S23

Coefficients:				
**Estimate**	**Std.**	**Error**	**z value**	**Pr(>|z|)**
rh2m	-5.769e-03	3.003e-04	-19.214	< 2e-16 ***
ns(time, 7)1	2.591e-01	2.593e-02	9.993	< 2e-16 ***
ns(time, 7)2	1.796e-01	3.346e-02	5.367	8.01e-08 ***
ns(time, 7)3	1.155e-01	3.058e-02	3.777	0.000159 ***
ns(time, 7)4	3.894e-02	3.218e-02	1.210	0.226337
ns(time, 7)5	3.395e-01	2.658e-02	12.774	< 2e-16 ***
ns(time, 7)6	-1.262e-01	5.681e-02	-2.222	0.026271 *
ns(time, 7)7	-5.533e-01	3.033e-02	-18.243	< 2e-16 ***
lon	-5.612e-01	4.287e-03	-130.910	< 2e-16 ***
lat	8.018e-02	4.531e-03	17.695	< 2e-16 ***

Signif. codes: 0 '***' 0.001 '**' 0.01 '*' 0.05 '.' 0.1 ' ' 1				

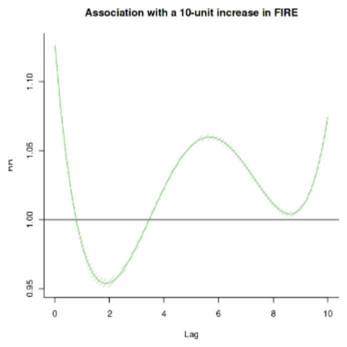

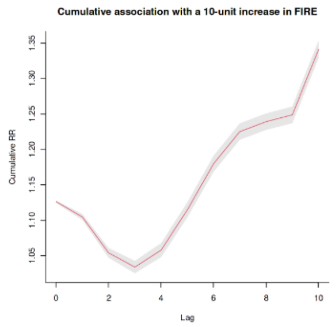

Table S24.

**lag 0**	**lag 0.2**	**lag 0.4**	**lag 0.6**	**lag 0.8**	**lag 1**	**lag 1.2**	**lag 1.4**
1.1245157	1.0807398	1.0451989	1.0168612	0.9947371	0.9779516	0.9657558	0.9575100
**lag 1.6**	**lag 1.8**	**lag 2**	**lag 2.2**	**lag 2.4**	**lag 2.6**	**lag 2.8**	**lag 3**
0.9526620	0.9507293	0.9512837	0.9539396	0.9583445	0.9641722	0.9711179	0.9788943
**lag 3.2**	**lag 3.4**	**lag 3.6**	**lag 3.8**	**lag 4**	**lag 4.2**	**lag 4.4**	**lag 4.6**
0.9872305	0.9958715	1.0045799	1.0131375	1.0213476	1.0290353	1.0360470	1.0422506
**lag 4.8**	**lag 5**	**lag 5.2**	**lag 5.4**	**lag 5.6**	**lag 5.8**	**lag 6**	**lag 6.2**
1.0475350	1.0518117	1.0550154	1.0571054	1.0580661	1.0579080	1.0566670	1.0544039
**lag 6.4**	**lag 6.6**	**lag 6.8**	**lag 7**	**lag 7.2**	**lag 7.4**	**lag 7.6**	**lag 7.8**
1.0512038	1.0471749	1.0424473	1.0371742	1.0315337	1.0257314	1.0199999	1.0145957
**lag 8**	**lag 8.2**	**lag 8.4**	**lag 8.6**	**lag 8.8**	**lag 9**	**lag 9.2**	**lag 9.4**
1.0097954	1.0058935	1.0032033	1.0020587	1.0028182	1.0058687	1.0116298	1.0205565
**lag 9.6**	**lag 9.8**	**lag 10**	**lag 0**	**lag 0.2**	**lag 0.4**	**lag 0.6**	**lag 0.8**
1.0331413	1.0499259	1.0715482	1.1279541	1.0842066	1.0493236	1.0216861	1.0001240
**lag 1**	**lag 1.2**	**lag 1.4**	**lag 1.6**	**lag 1.8**	**lag 2**	**lag 2.2**	**lag 2.4**
0.9837311	0.9717683	0.9636155	0.9587421	0.9566861	0.9570388	0.9594333	0.9635360
**lag 2.6**	**lag 2.8**	**lag 3**	**lag 3.2**	**lag 3.4**	**lag 3.6**	**lag 3.8**	**lag 4**
0.9690398	0.9756595	0.9831285	0.9911959	0.9996248	1.0081898	1.0166763	1.0248806
**lag 4.2**	**lag 4.4**	**lag 4.6**	**lag 4.8**	**lag 5**	**lag 5.2**	**lag 5.4**	**lag 5.6**
1.0326120	1.0396962	1.0459796	1.0513322	1.0556501	1.0588572	1.0609063	1.0617802
**lag 5.8**	**lag 6**	**lag 6.2**	**lag 6.4**	**lag 6.6**	**lag 6.8**	**lag 7**	**lag 7.2**
1.0614913	1.0600824	1.0576259	1.0542235	1.0500047	1.0451255	1.0397641	1.0341167
**lag 7.4**	**lag 7.6**	**lag 7.8**	**lag 8**	**lag 8.2**	**lag 8.4**	**lag 8.6**	**lag 8.8**
1.0283917	1.0228083	1.0175983	1.0130099	1.0093110	1.0067918	1.0057682	1.0065866
**lag 9**	**lag 9.2**	**lag 9.4**	**lag 9.6**	**lag 9.8**	**lag 10**		
1.0096319	1.0153391	1.0242118	1.0368484	1.0539661	1.0764004		

Statistical analysis

Distributed Lag Non-linear Models

Impact of emissions from forest fires on hospitalizations for respiratory diseases

Without Control of Seasonality Bias - Respiratory Diseases (5 years analisys)

Table S25.

pred1.fire$allRRfit["10"]	1.3411310
cbind(pred1.fire$allRRlow, pred1.fire$allRRhigh)["10",]	1.328906 1.353468

Table S26.

pred1.fire$cumRRlow["10",]

lag 0	lag 1	lag 2	lag 3	lag 4	lag 5	lag 6	lag 7	lag 8	lag 9	lag 10
1.1245157	1.1005720	1.0471892	1.0253322	1.0481095	1.1041473	1.1686031	1.2137115	1.2276447	1.2370097	1.3289065

pred1.fire$cumRRhigh["10",]

lag 0	lag 1	lag 2	lag 3	lag 4	lag 5	lag 6	lag 7	lag 8	lag 9	lag 10
1.1279541	1.1087464	1.0608773	1.0427297	1.0677672	1.1254189	1.1911102	1.2367731	1.2507770	1.2606190	1.3534683

Statistical analysis

Distributed Lag Non-linear Models

Impact of emissions from forest fires on hospitalizations for respiratory diseases

Without Control of Seasonality Bias - Respiratory Diseases (10 years analisys)

Table S27.

Coefficients:				
Estimate	Std.	Error	z value	Pr(>|z|)
(Intercept)	-3.828e+01	1.505e-01	-254.336	< 2e-16 ***
cb1.firev1.l1	7.756e-03	7.083e-05	109.502	< 2e-16 ***
cb1.firev1.l2	-1.140e-01	1.852e-03	-61.535	< 2e-16 ***
cb1.firev1.l3	5.123e-01	9.710e-03	52.763	< 2e-16 ***
cb1.firev1.l4	-8.003e-01	1.591e-02	-50.310	< 2e-16 ***
cb1.firev1.l5	3.972e-01	8.087e-03	49.110	< 2e-16 ***
cb1.pm10v1.l1	-7.290e-02	7.096e-03	-10.274	< 2e-16 ***
cb1.pm10v1.l2	8.803e-01	1.173e-01	7.506	6.08e-14 ***
cb1.pm10v1.l3	-1.463e+00	4.662e-01	-3.139	0.001694 **
cb1.pm10v1.l4	-5.220e-01	6.681e-01	-0.781	0.434664
cb1.pm10v1.l5	1.266e+00	3.153e-01	4.014	5.96e-05 ***
cb1.pm25v1.l1	3.182e-01	2.853e-02	11.152	< 2e-16 ***
cb1.pm25v1.l2	-5.309e+00	4.704e-01	-11.288	< 2e-16 ***
cb1.pm25v1.l3	1.609e+01	1.869e+00	8.611	< 2e-16 ***
cb1.pm25v1.l4	-1.577e+01	2.680e+00	-5.885	3.97e-09 ***
cb1.pm25v1.l5	4.490e+00	1.265e+00	3.549	0.000387 ***
cb1.so2v1.l1	-8.900e-01	1.449e-02	-61.414	< 2e-16 ***
cb1.so2v1.l2	1.105e+01	2.969e-01	37.204	< 2e-16 ***
cb1.so2v1.l3	-2.622e+01	1.343e+00	-19.514	< 2e-16 ***
cb1.so2v1.l4	1.447e+01	2.061e+00	7.020	2.22e-12 ***
cb1.so2v1.l5	2.462e+00	1.007e+00	2.443	0.014546 *
cb1.no2v1.l1	-2.018e-01	3.946e-02	-5.114	3.16e-07 ***
cb1.no2v1.l2	-3.559e+00	6.512e-01	-5.465	4.62e-08 ***
cb1.no2v1.l3	1.915e+01	2.778e+00	6.893	5.47e-12 ***
cb1.no2v1.l4	-2.835e+01	4.237e+00	-6.691	2.22e-11 ***
cb1.no2v1.l5	1.314e+01	2.095e+00	6.272	3.57e-10 ***
cb1.t2mv1.l1	-1.011e+01	2.157e-01	-46.869	< 2e-16 ***
cb1.t2mv1.l2	1.368e+01	2.923e-01	46.806	< 2e-16 ***
cb1.t2mv2.l1	-7.310e+00	2.159e-01	-33.850	< 2e-16 ***
cb1.t2mv2.l2	9.958e+00	2.928e-01	34.010	< 2e-16 ***
cb1.t2mv3.l1	1.004e+00	1.570e-01	6.396	1.60e-10 ***
cb1.t2mv3.l2	-1.384e+00	2.111e-01	-6.555	5.57e-11 ***
cb1.t2mv4.l1	-2.222e+01	4.379e-01	-50.739	< 2e-16 ***
cb1.t2mv4.l2	2.972e+01	5.930e-01	50.122	< 2e-16 ***
cb1.t2mv5.l1	-9.711e+00	2.661e-01	-36.499	< 2e-16 ***
cb1.t2mv5.l2	1.239e+01	3.526e-01	35.130	< 2e-16 ***

Table S28.

Coefficients:				
Estimate	Std.	Error	z value	Pr(>|z|)
rh2m	-1.689e-03	2.016e-04	-8.381	< 2e-16 ***
ns(time, 7)1	-4.487e-01	1.665e-02	-26.944	< 2e-16 ***
ns(time, 7)2	-4.039e-01	2.178e-02	-18.543	< 2e-16 ***
ns(time, 7)3	-3.461e-01	1.949e-02	-17.761	< 2e-16 ***
ns(time, 7)4	-2.836e-01	2.030e-02	-13.971	< 2e-16 ***
ns(time, 7)5	-2.423e-01	1.758e-02	-13.785	< 2e-16 ***
ns(time, 7)6	-6.443e-01	3.244e-02	-19.862	< 2e-16 ***
ns(time, 7)7	-4.226e-01	1.724e-02	-24.508	< 2e-16 ***
lon	-6.639e-01	2.814e-03	-235.932	< 2e-16 ***
lat	1.787e-01	3.061e-03	58.389	< 2e-16 ***

Signif. codes: 0 '***' 0.001 '**' 0.01 '*' 0.05 '.' 0.1 ' ' 1				

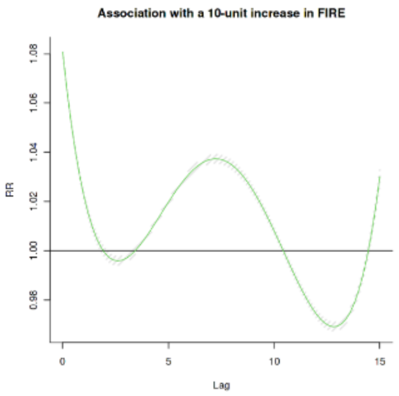

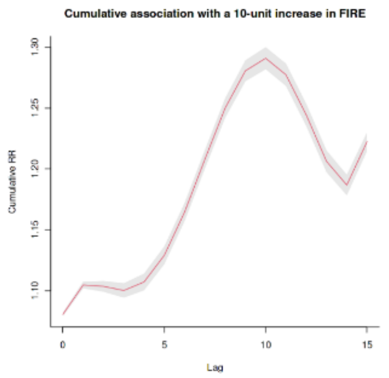

Table S29.

lag 0	lag 0.2	lag 0.4	lag 0.6	lag 0.8	lag 1	lag 1.2	lag 1.4
1.0791491	1.0639348	1.0505977	1.0390135	1.0290661	1.0206344	1.0135956	1.0078307

lag 1.6	lag 1.8	lag 2	lag 2.2	lag 2.4	lag 2.6	lag 2.8	lag 3
1.0032259	0.9996746	0.9970762	0.9953364	0.9943666	0.9940827	0.9944053	0.9952587

lag 3.2	lag 3.4	lag 3.6	lag 3.8	lag 4	lag 4.2	lag 4.4	lag 4.6
0.9965707	0.9982724	1.0002972	1.0025816	1.0050645	1.0076877	1.0103962	1.0131391

lag 4.8	lag 5	lag 5.2	lag 5.4	lag 5.6	lag 5.8	lag 6	lag 6.2
1.0158697	1.0185459	1.0211294	1.0235856	1.0258828	1.0279921	1.0298867	1.0315425

lag 6.4	lag 6.6	lag 6.8	lag 7	lag 7.2	lag 7.4	lag 7.6	lag 7.8
1.0329381	1.0340546	1.0348761	1.0353894	1.0355847	1.0354550	1.0349966	1.0342089

lag 8	lag 8.2	lag 8.4	lag 8.6	lag 8.8	lag 9	lag 9.2	lag 9.4
1.0330944	1.0316590	1.0299114	1.0278634	1.0255300	1.0229287	1.0200801	1.0170074

lag 9.6	lag 9.8	lag 10	lag 0	lag 0.2	lag 0.4	lag 0.6	lag 0.8
1.0137364	1.0102959	1.0067171	1.0821496	1.0666726	1.0533481	1.0419317	1.0322073

lag 1	lag 1.2	lag 1.4	lag 1.6	lag 1.8	lag 2	lag 2.2	lag 2.4
1.0239945	1.0171418	1.0115176	1.0070047	1.0034970	1.0008971	0.9991151	0.9980672

lag 2.6	lag 2.8	lag 3	lag 3.2	lag 3.4	lag 3.6	lag 3.8	lag 4
0.9976751	0.9978650	0.9985678	0.9997181	1.0012540	1.0031173	1.0052527	1.0076079

lag 4.2	lag 4.4	lag 4.6	lag 4.8	lag 5	lag 5.2	lag 5.4	lag 5.6
1.0101328	1.0127794	1.0155007	1.0182506	1.0209834	1.0236550	1.0262229	1.0286469

lag 5.8	lag 6	lag 6.2	lag 6.4	lag 6.6	lag 6.8	lag 7	lag 7.2
1.0308904	1.0329199	1.0347053	1.0362204	1.0374425	1.0383526	1.0389355	1.0391796

lag 7.4	lag 7.6	lag 7.8	lag 8	lag 8.2	lag 8.4	lag 8.6	lag 8.8
1.0390770	1.0386239	1.0378198	1.0366683	1.0351765	1.0333552	1.0312191	1.0287863

lag 9	lag 9.2	lag 9.4	lag 9.6	lag 9.8	lag 10
1.0260784	1.0231206	1.0199414	1.0165724	1.0130480	1.0094055

Statistical analysis

Distributed Lag Non-linear Models

Impact of emissions from forest fires on hospitalizations for respiratory diseases

Without Control of Seasonality Bias - Respiratory Diseases (10 years analisys)

Table S30.

pred1.fire$allRRfit["10"]	1.2224147
cbind(pred1.fire$allRRlow, pred1.fire$allRRhigh)["10",]	1.214738 1.230139

Table S31.

pred1.fire$cumRRlow["10",]

lag 0	lag 1	lag 2	lag 3	lag 4	lag 5	lag 6	lag 7	lag 8	lag 9	lag 10
1.0791491	1.1019734	1.0992001	1.0943345	1.1003602	1.1218015	1.1571049	1.2002051	1.2419231	1.2719879	1.2818750

pred1.fire$cumRRhigh["10",]

lag 0	lag 1	lag 2	lag 3	lag 4	lag 5	lag 6	lag 7	lag 8	lag 9	lag 10
1.0821496	1.1075555	1.1080966	1.1061597	1.1140855	1.1364143	1.1720227	1.2154738	1.2580161	1.2892104	1.2999728

Fig. S1 - Annual evolution of fire outbreaks in the city of Aquidauana - Mato Grosso do Sul.

2010

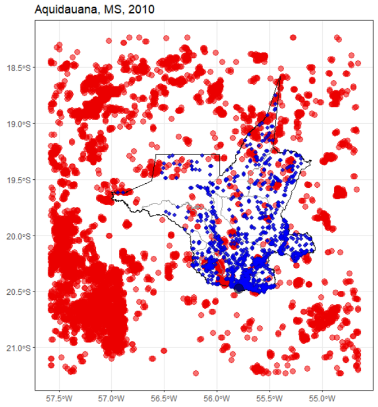

2011

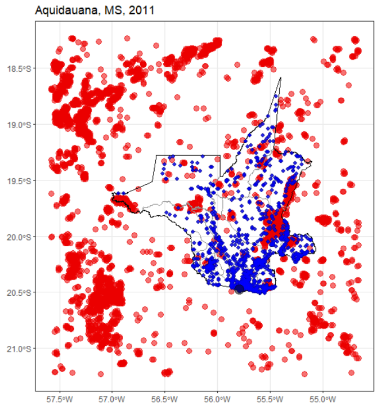

2012

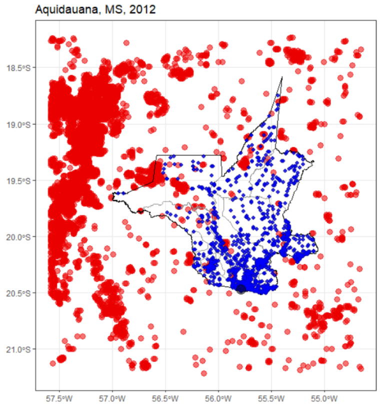

2013

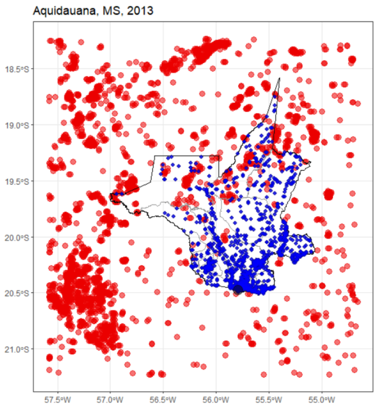

2014

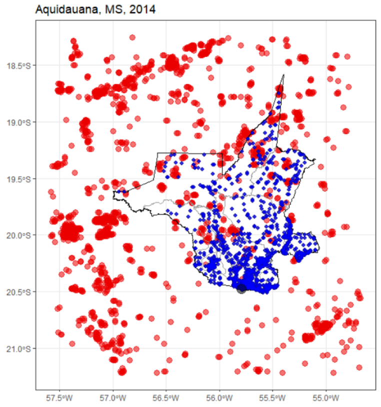

2015

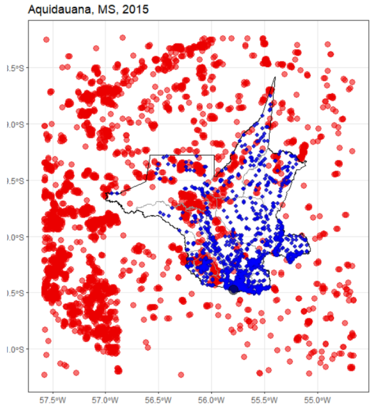

2016

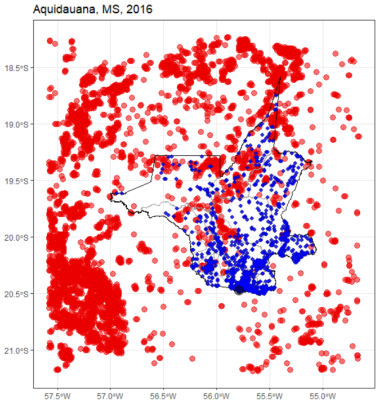

2017

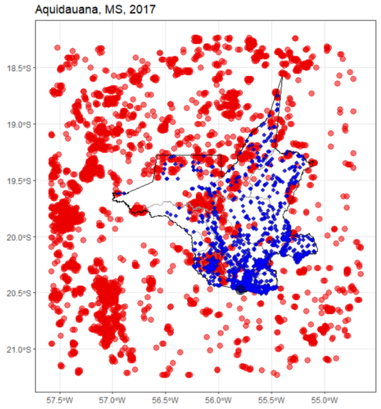

2018

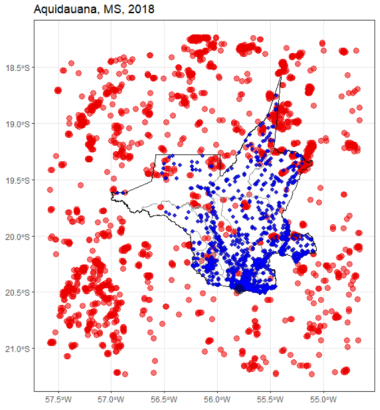

2019

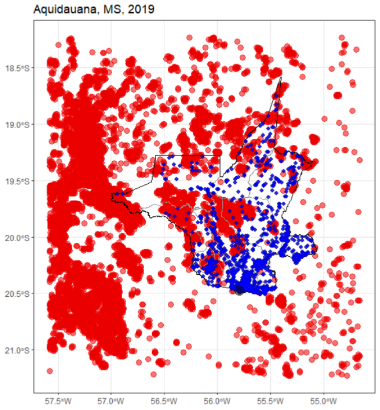

Year	Fire Outbreaks
2010	847
2011	1775
2012	1489
2013	1527
2014	1305
2015	3205
2016	2685
2017	5314
2018	2359
2019	28063

Wildfire Overview

Fig. S2 - Annual evolution of fire outbreaks in the city of Barão de Melgaço - Mato Grosso.

2010

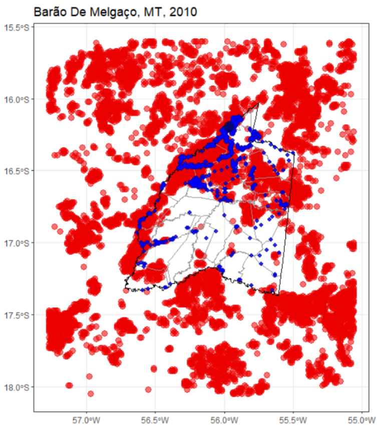

2011

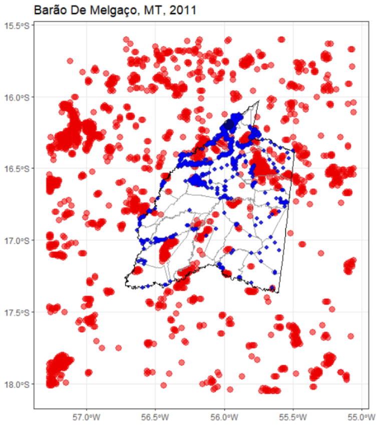

2012

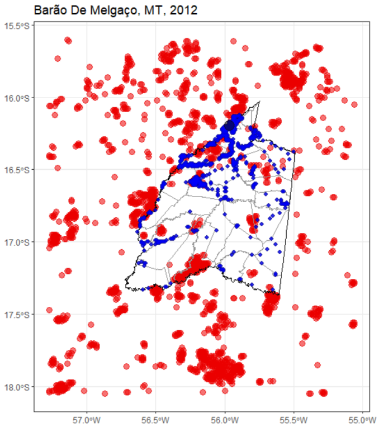

2013

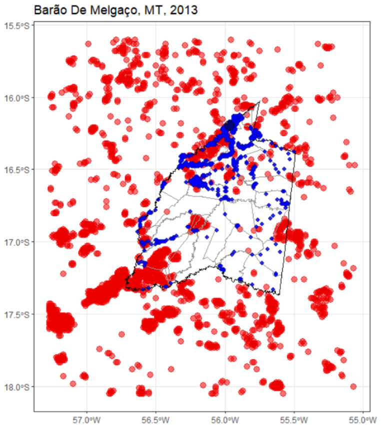

2014

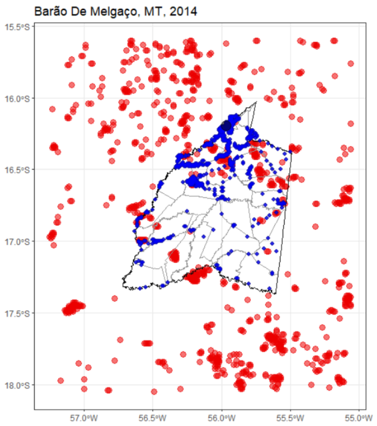

2015

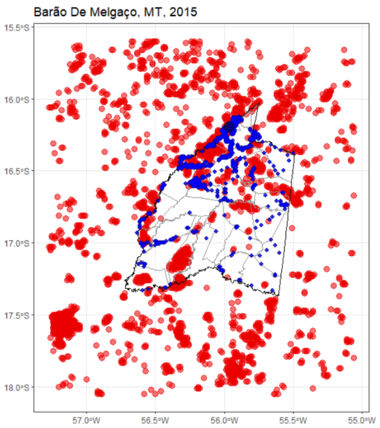

2016

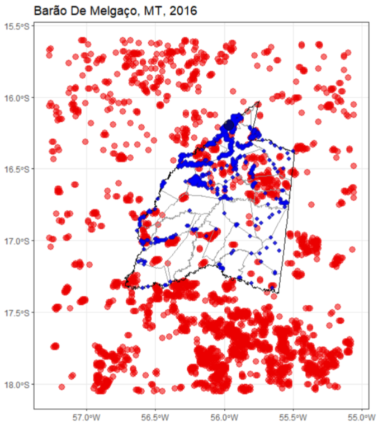

2017

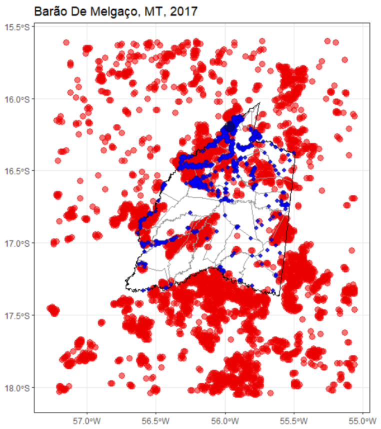

2018

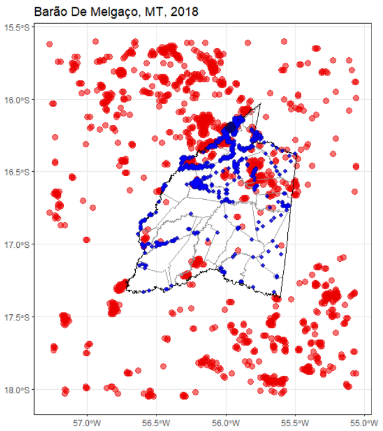

2019

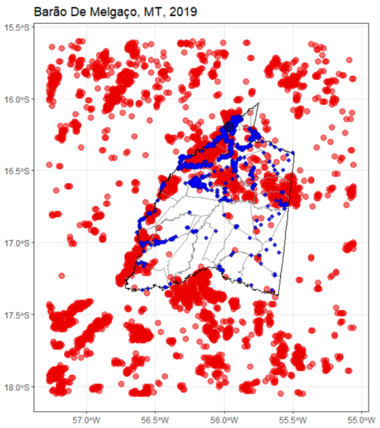

Year	Fire Outbreaks
2010	4942
2011	1041
2012	2193
2013	6088
2014	1309
2015	4173
2016	2749
2017	13696
2018	2342
2019	12604

Wildfire Overview

Fig. S3 - Annual evolution of fire outbreaks in the city of Bodoquena - Mato Grosso do Sul.

2010

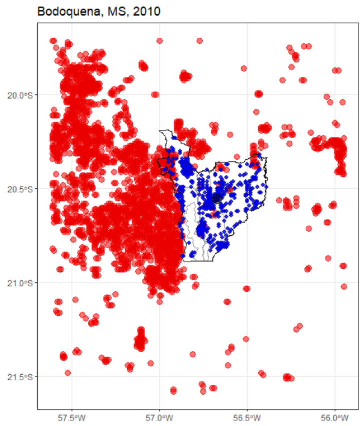

2011

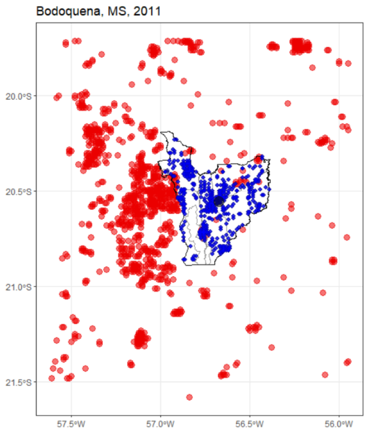

2012

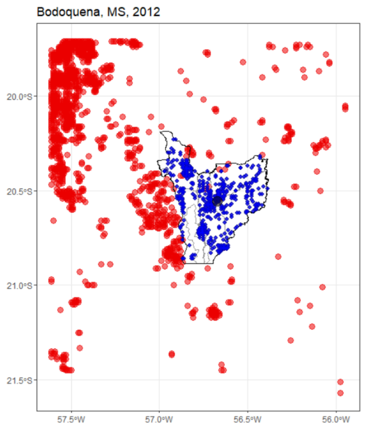

2013

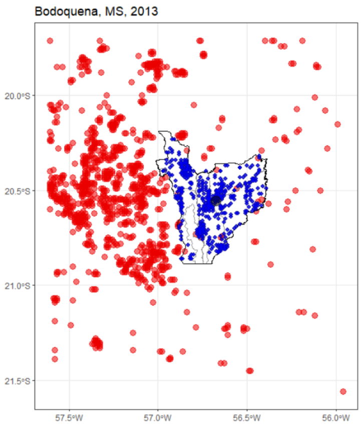

2014

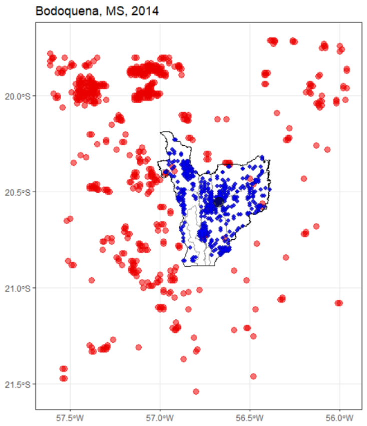

2015

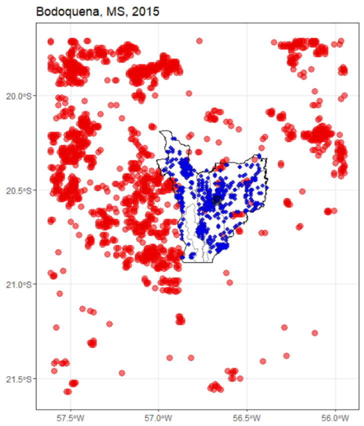

2016

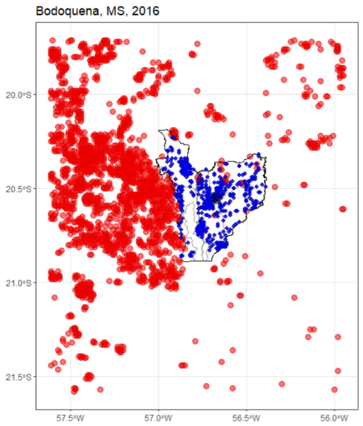

2017

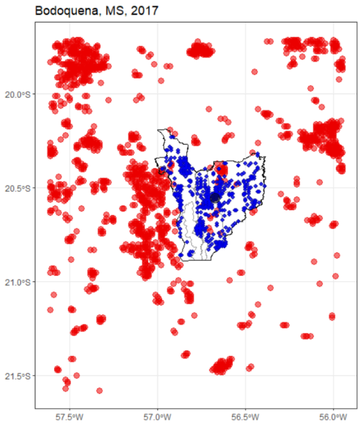

2018

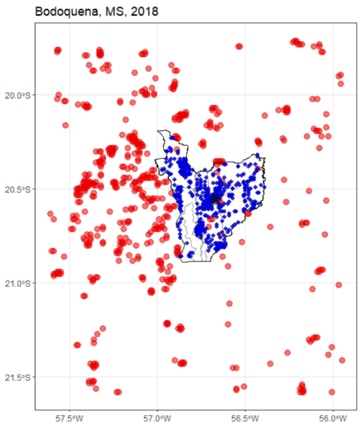

2019

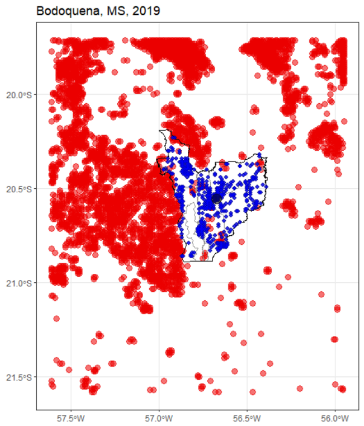

Year	Fire Outbreaks
2010	179
2011	65
2012	63
2013	89
2014	68
2015	179
2016	184
2017	224
2018	92
2019	569

Wildfire Overview

Fig. S4 - Annual evolution of fire outbreaks in the city of Cáceres - Mato Grosso.

2010

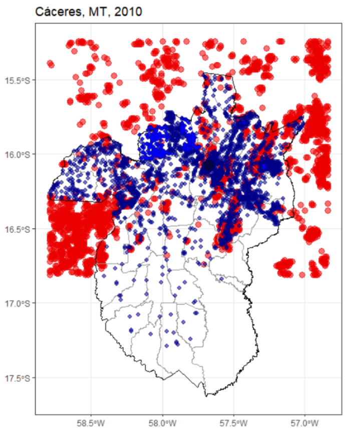

2011

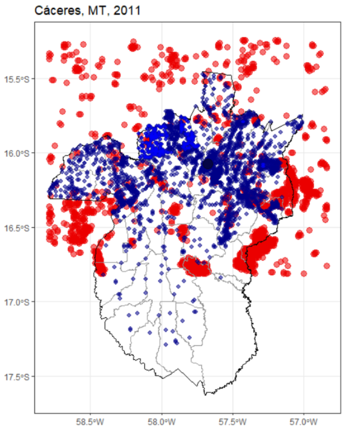

2012

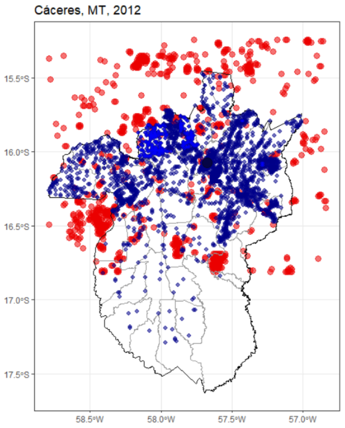

2013

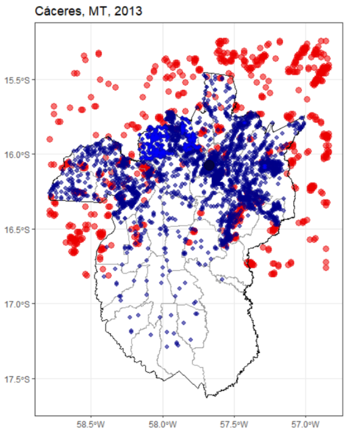

2014

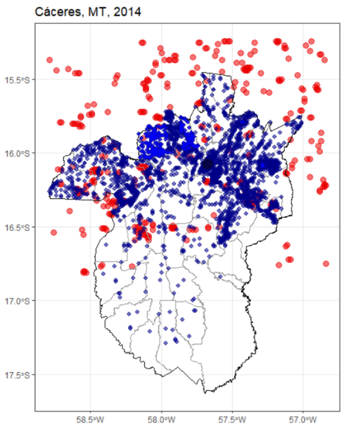

2015

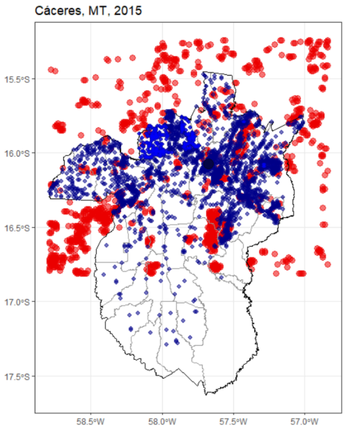

2016

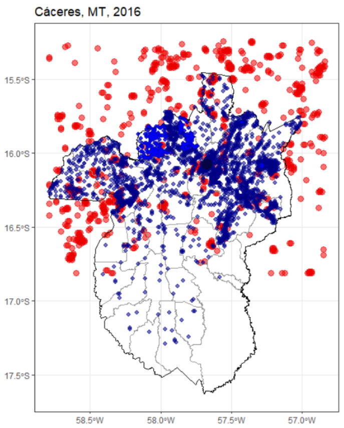

2017

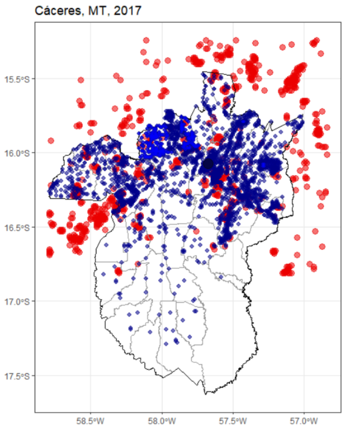

2018

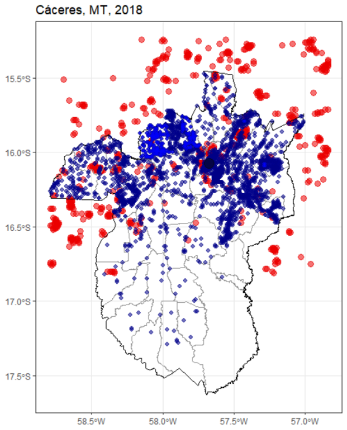

2019

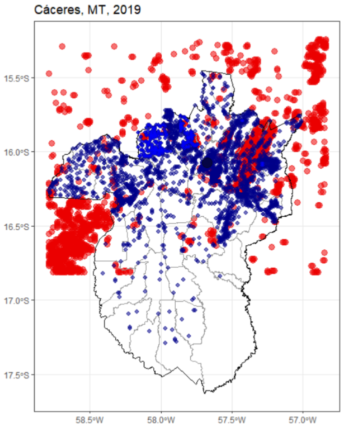

Year	Fire Outbreaks
2010	3343
2011	4972
2012	9434
2013	3684
2014	1325
2015	5734
2016	2806
2017	3796
2018	3156
2019	21704

Wildfire Overview

Fig. S5 - Annual evolution of fire outbreaks in the city of Corumbá - Mato Grosso do Sul.

2010

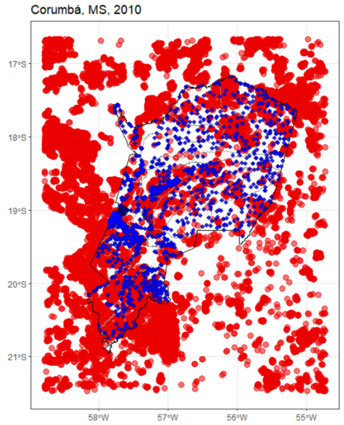

2011

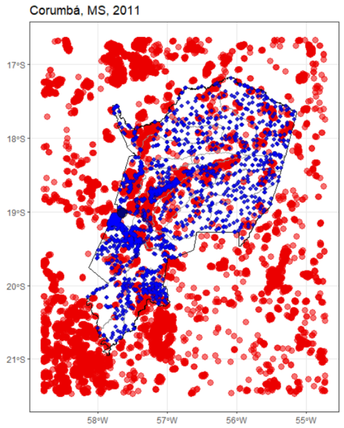

2012

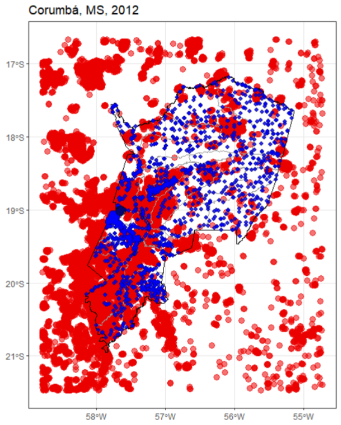

2013

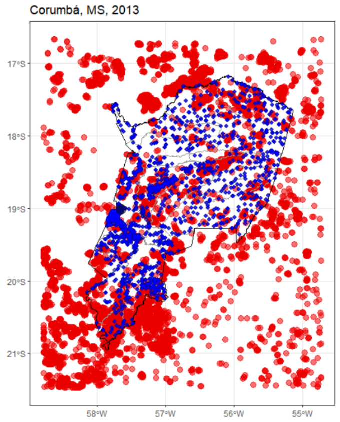

2014

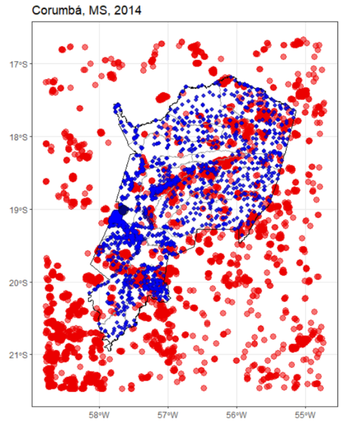

2015

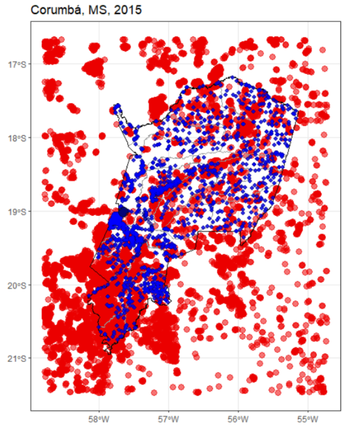

2016

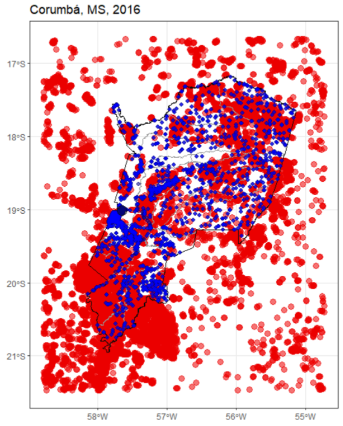

2017

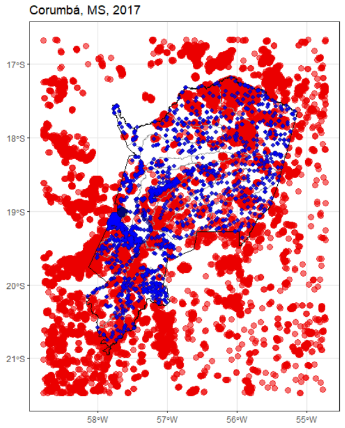

2018

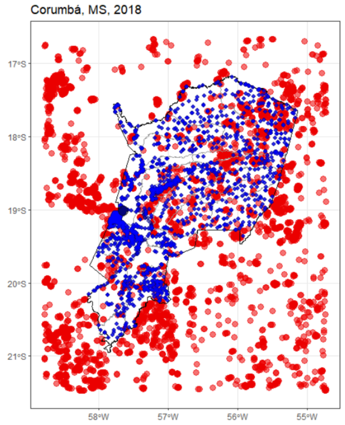

2019

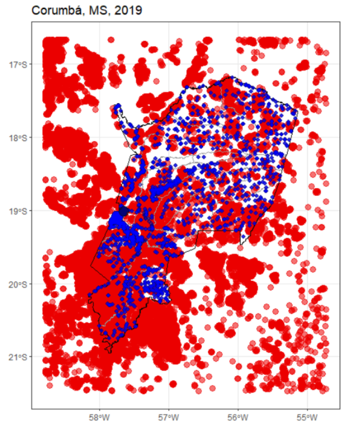

Year	Fire Outbreaks
2010	12238
2011	6357
2012	70020
2013	19255
2014	9149
2015	27708
2016	42325
2017	39409
2018	9143
2019	141939

Wildfire Overview

Fig. S6 - Annual evolution of fire outbreaks in the city of Coxim - Mato Grosso do Sul.

2010

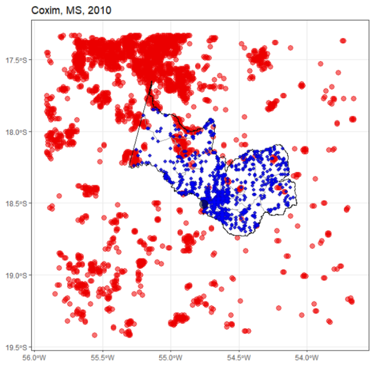

2011

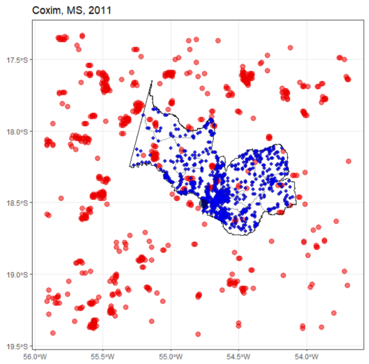

2012

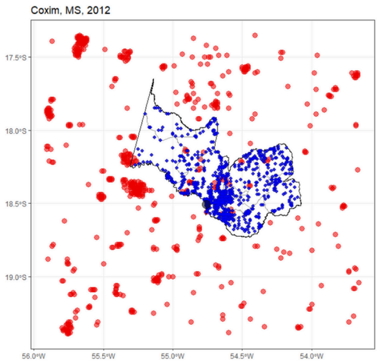

2013

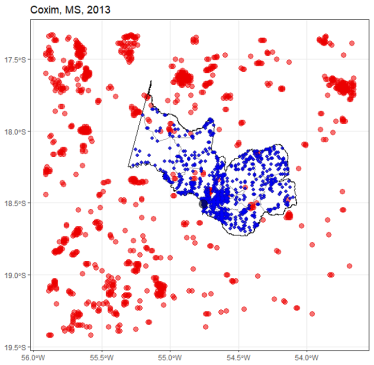

2014

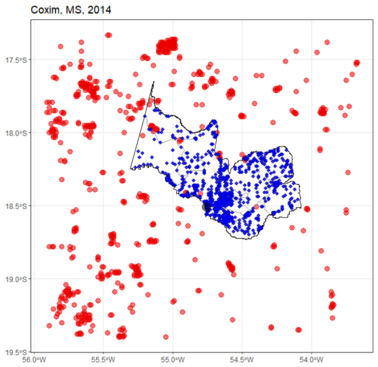

2015

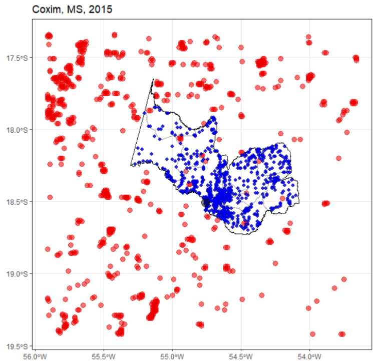

2016

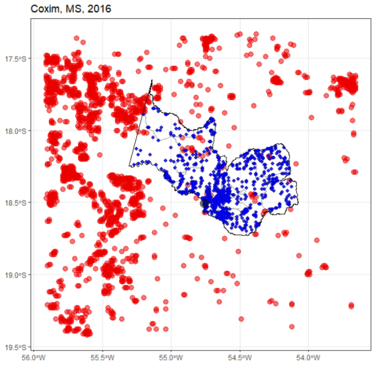

2017

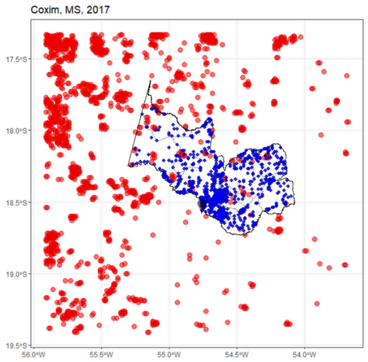

2018

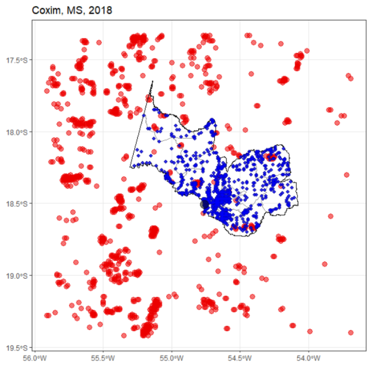

2019

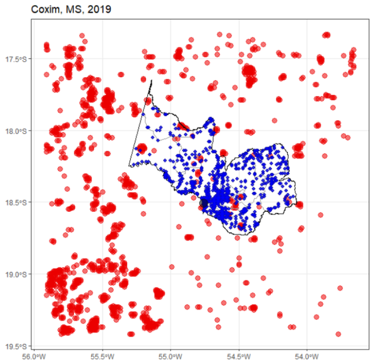

Year	Fire Outbreaks
2010	484
2011	193
2012	209
2013	304
2014	140
2015	180
2016	280
2017	569
2018	647
2019	894

Fig. S7 - Annual evolution of fire outbreaks in the city of Cuverlândia - Mato Grosso.

2010

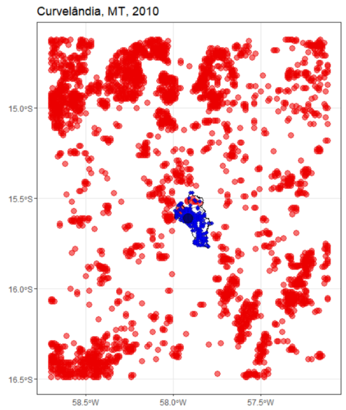

2011

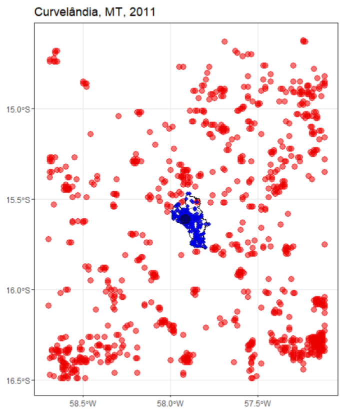

2012

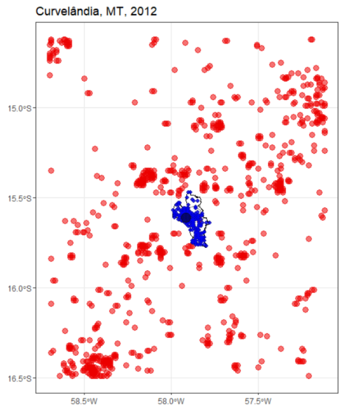

2013

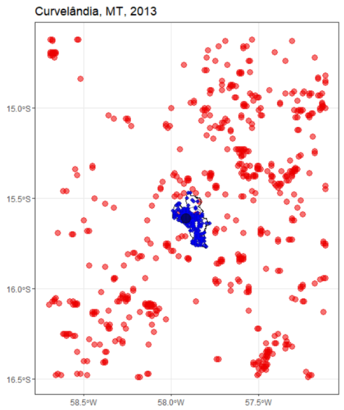

2014

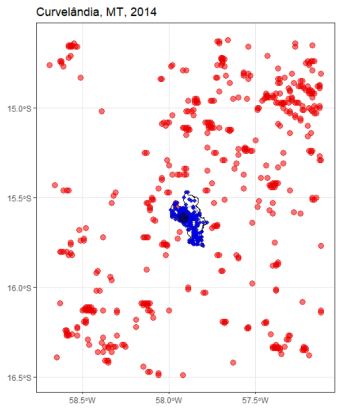

2015

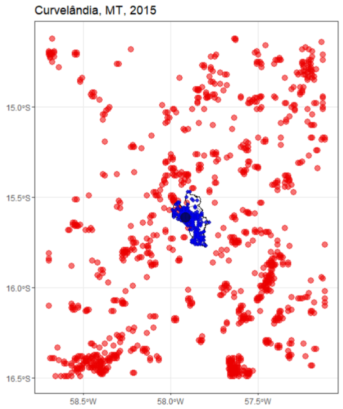

2016

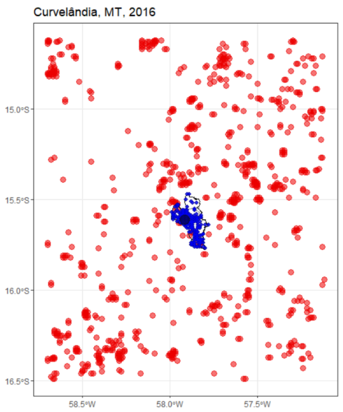

2017

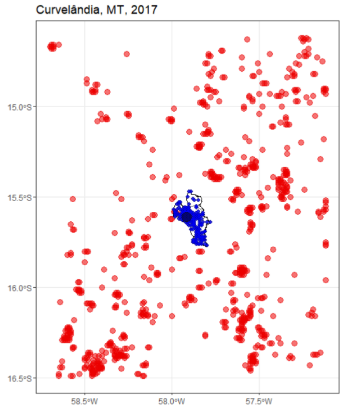

2018

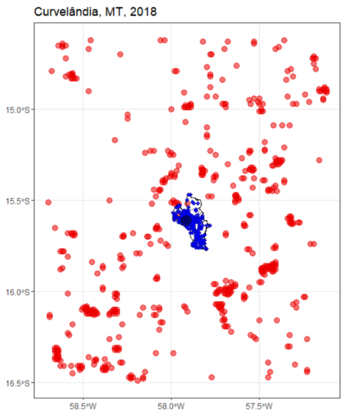

2019

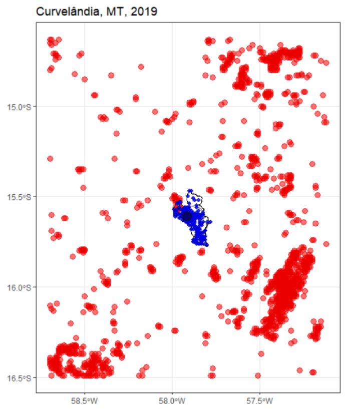

Year	Fire Outbreaks
2010	27
2011	10
2012	20
2013	22
2014	10
2015	24
2016	25
2017	29
2018	33
2019	90

Fig. S8 - Annual evolution of fire outbreaks in the city of Glória D'Oeste - Mato Grosso.

2010

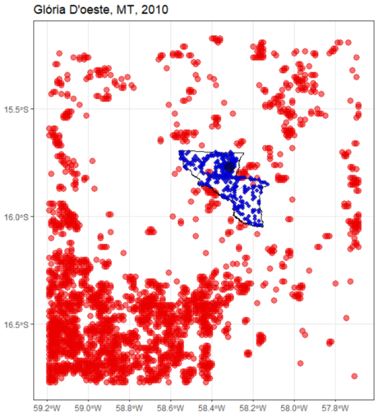

2011

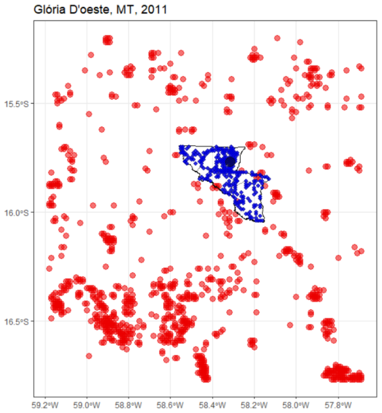

2012

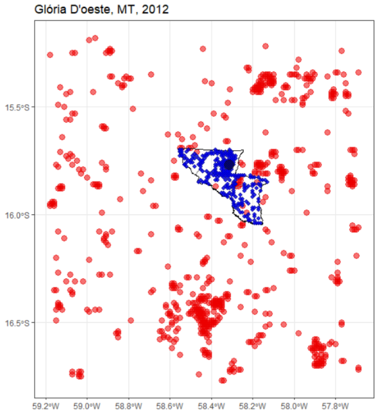

2013

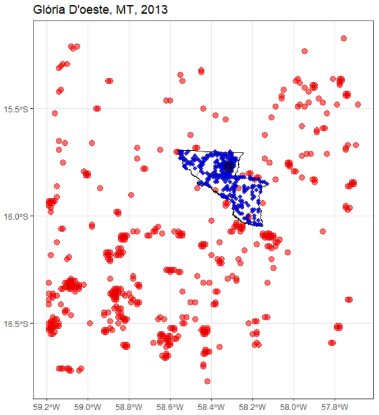

2014

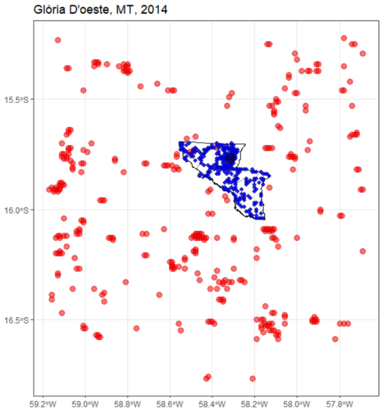

2015

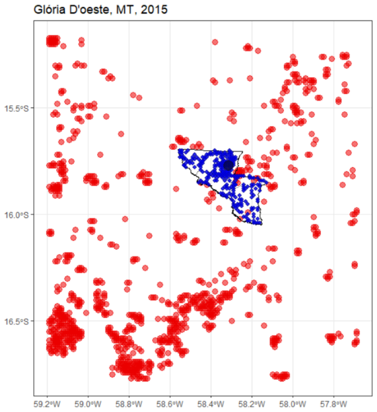

2016

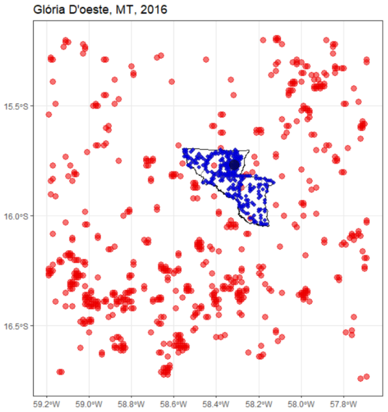

2017

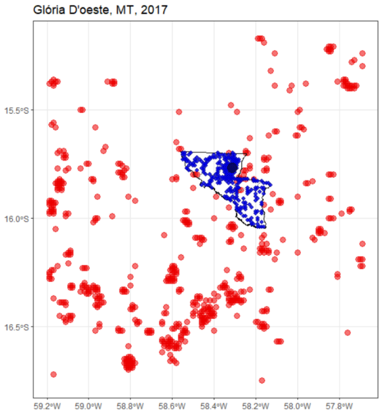

2018

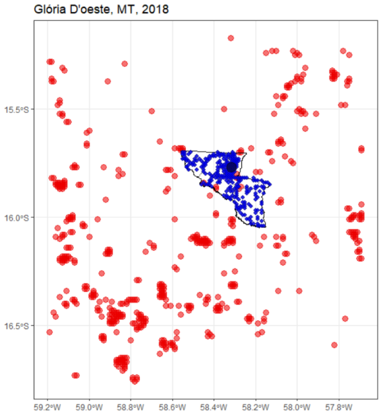

2019

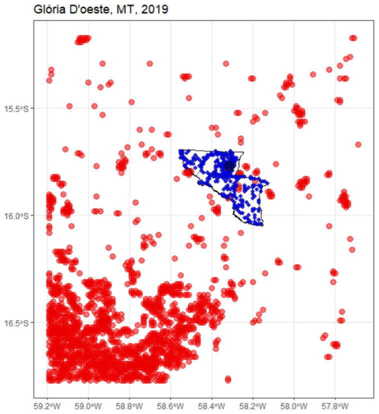

Year	Fire Outbreaks
2010	24
2011	22
2012	88
2013	54
2014	30
2015	46
2016	33
2017	53
2018	66
2019	105

Wildfire Overview

Fig. S9 - Annual evolution of fire outbreaks in the city of Itiquira - Mato Grosso.

2010

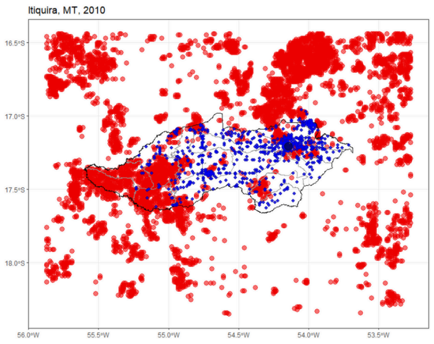

2011

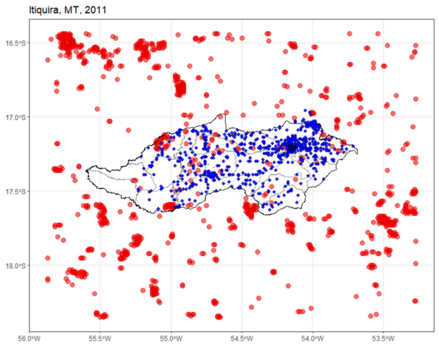

2012

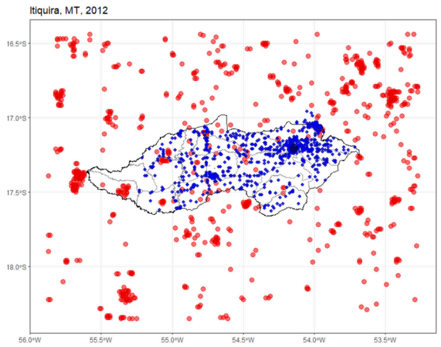

2013

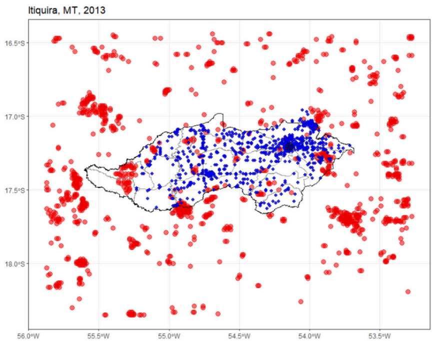

2014

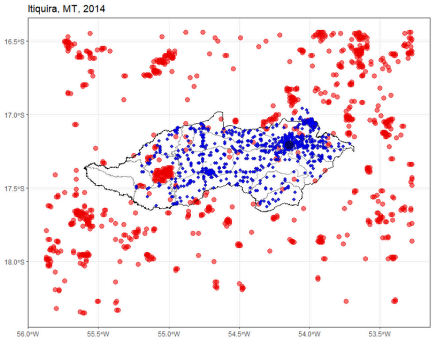

2015

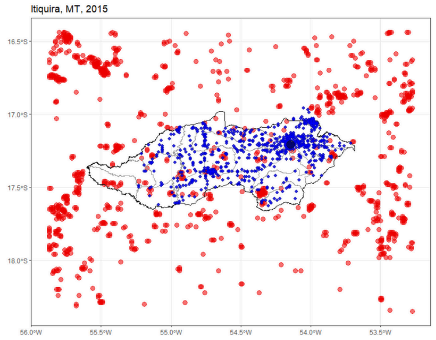

2016

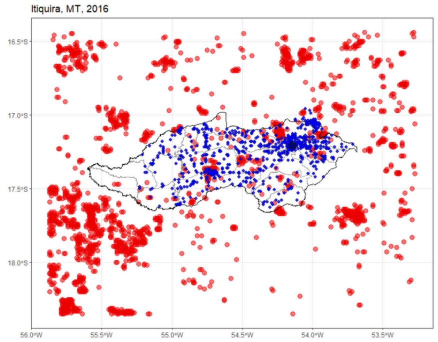

2017

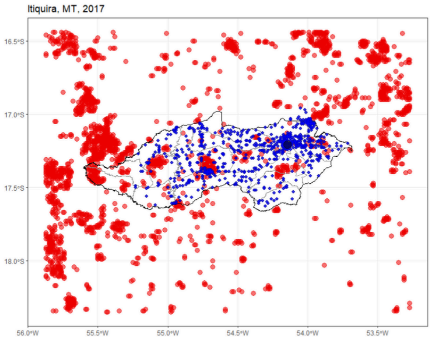

2018

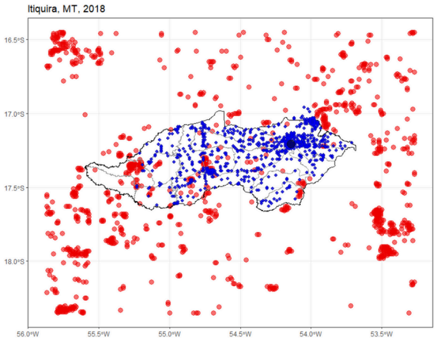

2019

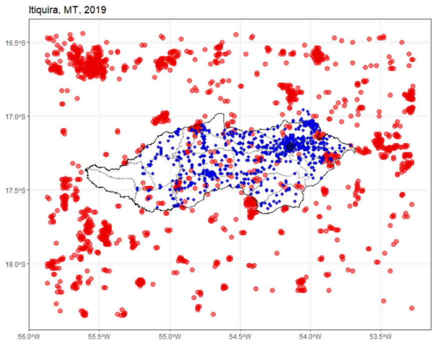

Year	Fire Outbreaks
2010	3195
2011	268
2012	486
2013	1365
2014	716
2015	772
2016	1189
2017	4810
2018	1355
2019	1032

Wildfire Overview

Fig. S10 - Annual evolution of fire outbreaks in the city of Juscimeira - Mato Grosso.

2010

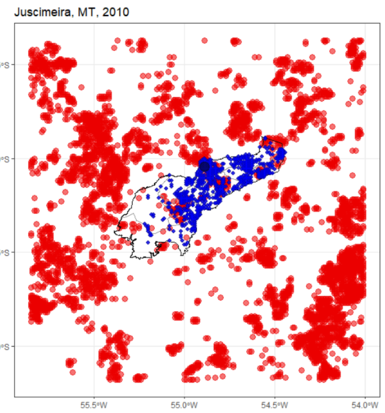

2011

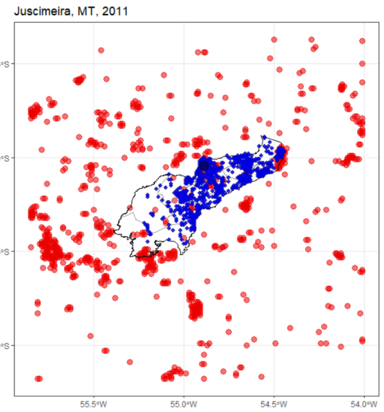

2012

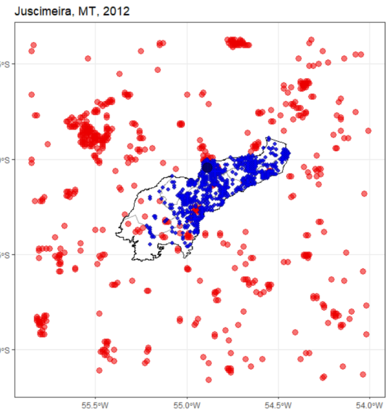

2013

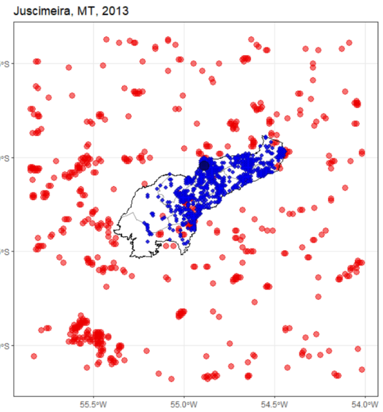

2014

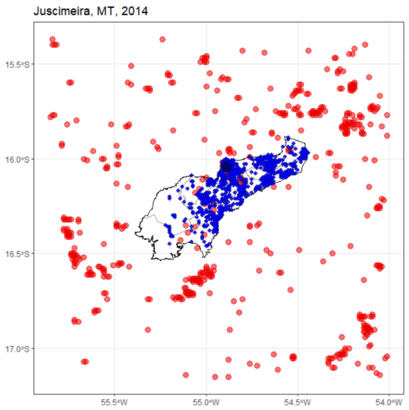

2015

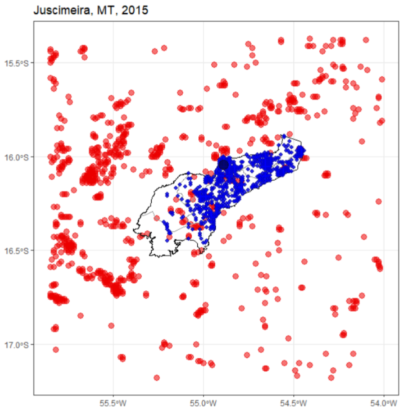

2016

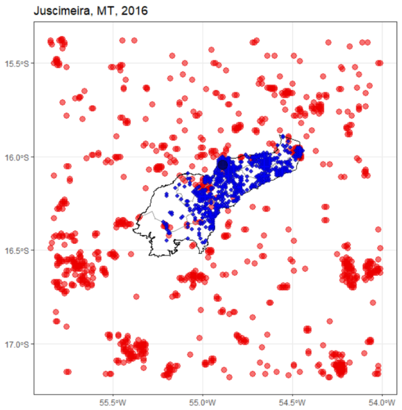

2017

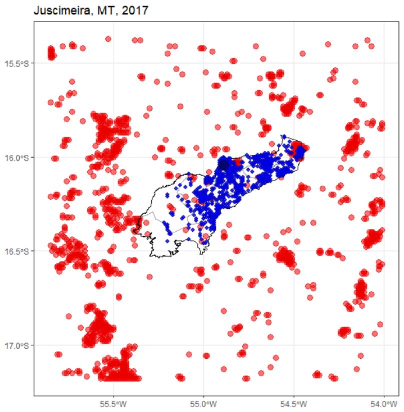

2018

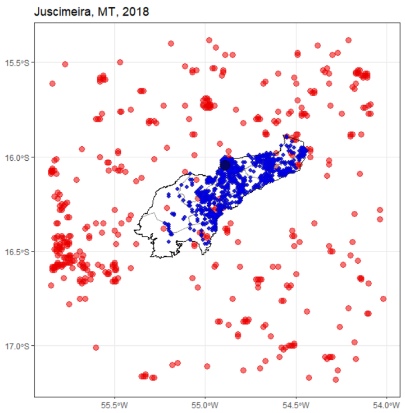

2019

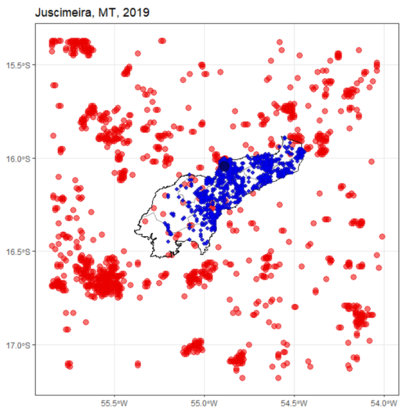

Year	Fire Outbreaks
2010	401
2011	126
2012	170
2013	197
2014	128
2015	284
2016	438
2017	534
2018	98
2019	217

Fig. S11 - Annual evolution of fire outbreaks in the city of Lambari D'Oeste - Mato Grosso.

2010

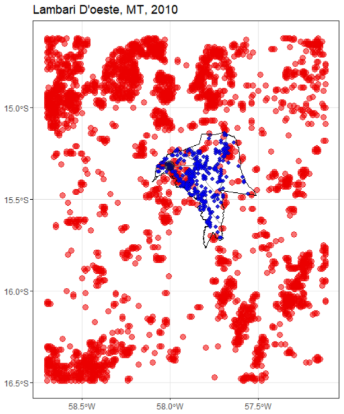

2011

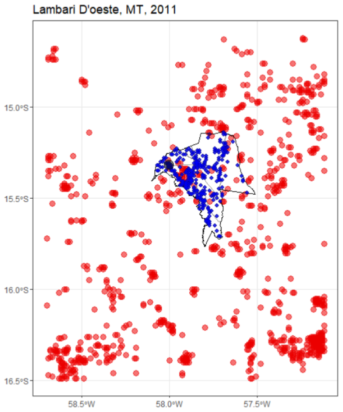

2012

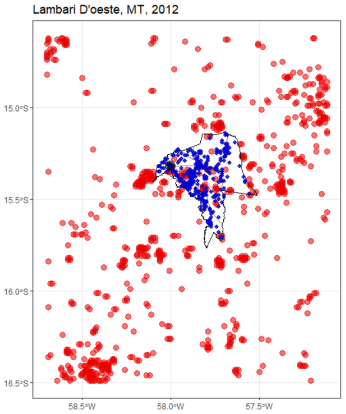

2013

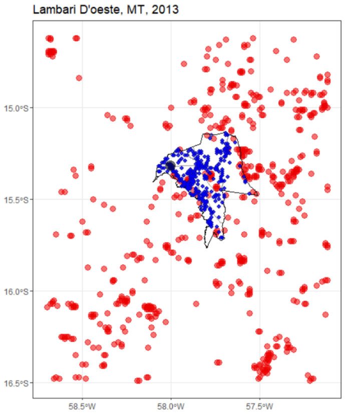

2014

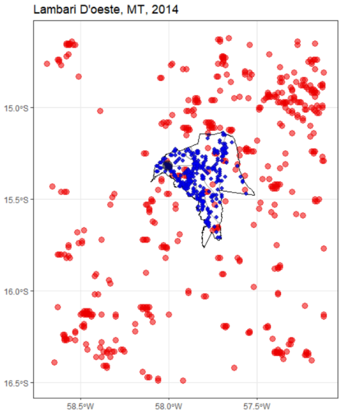

2015

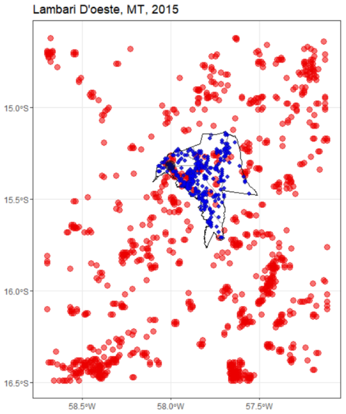

2016

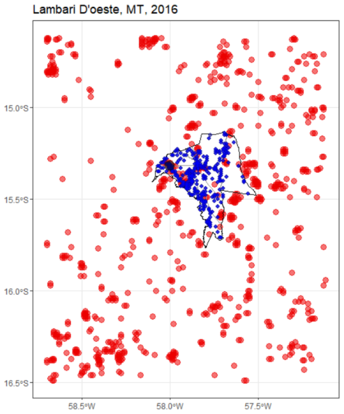

2017

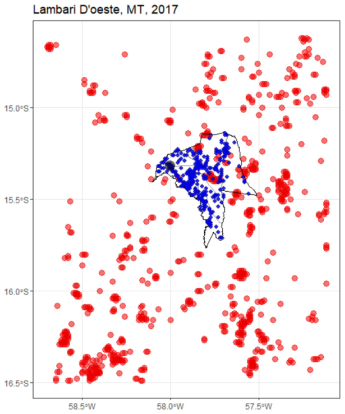

2018

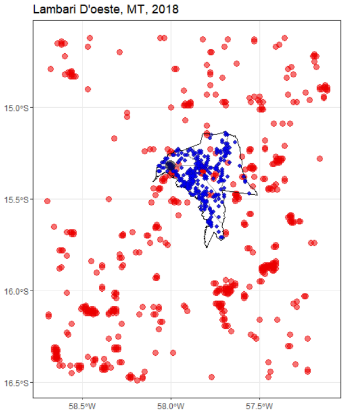

2019

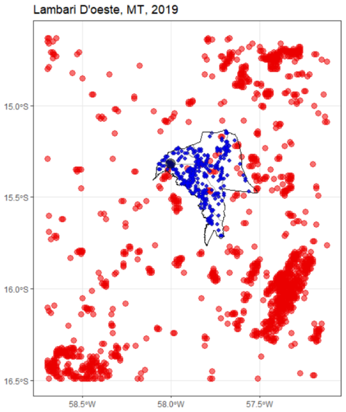

Year	Fire Outbreaks
2010	207
2011	160
2012	302
2013	282
2014	135
2015	212
2016	297
2017	384
2018	317
2019	364

Wildfire Overview

Fig. S12 - Annual evolution of fire outbreaks in the city of Miranda - Mato Grosso do Sul.

2010

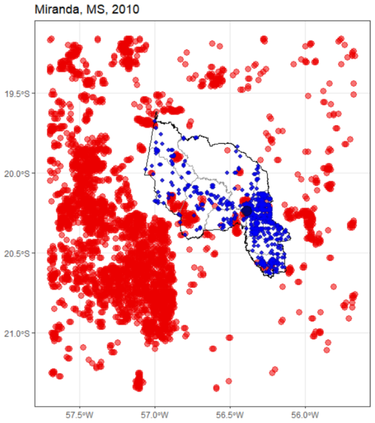

2011

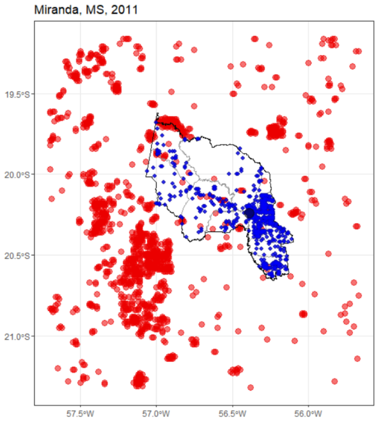

2012

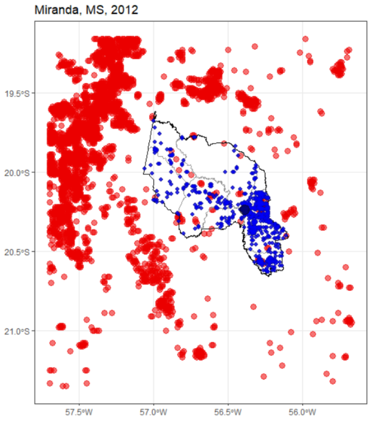

2013

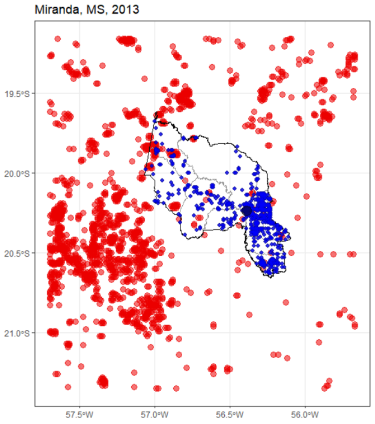

2014

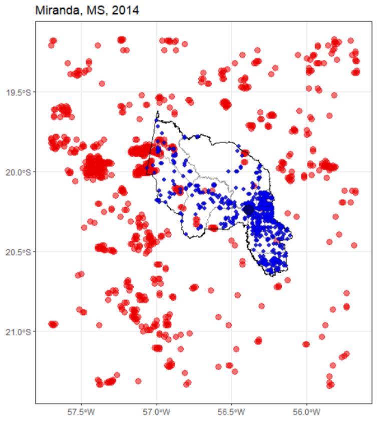

2015

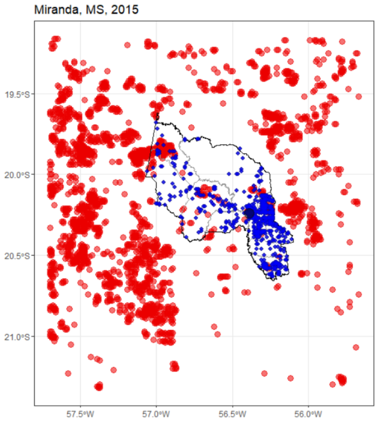

2016

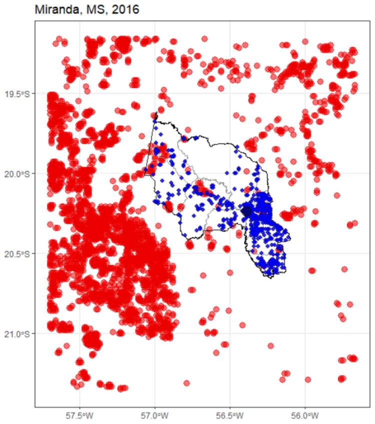

2017

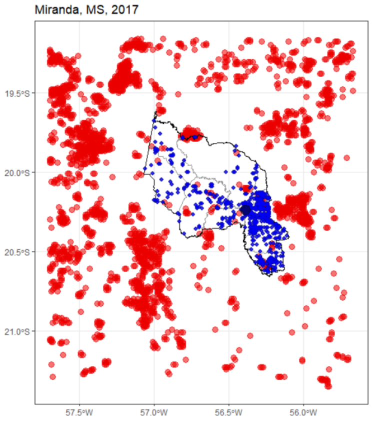

2018

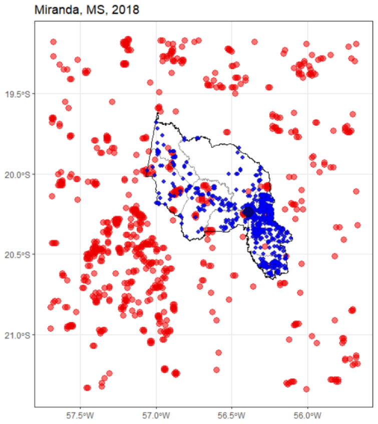

2019

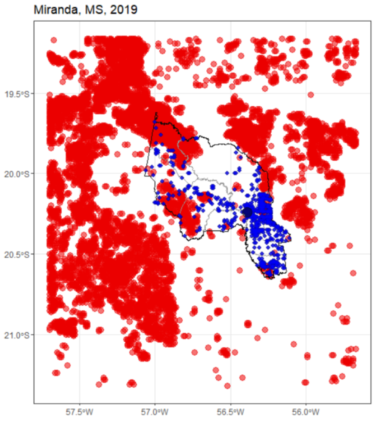

Year	Fire Outbreaks
2010	340
2011	222
2012	291
2013	665
2014	602
2015	894
2016	518
2017	602
2018	599
2019	13091

Wildfire Overview

Fig. S13 - Annual evolution of fire outbreaks in the city of Mirassol D’Oeste - Mato Grosso.

2010

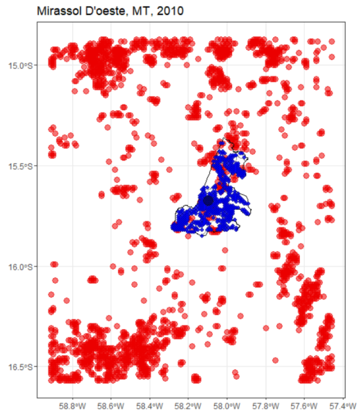

2011

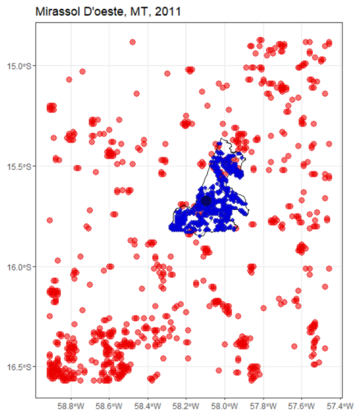

2012

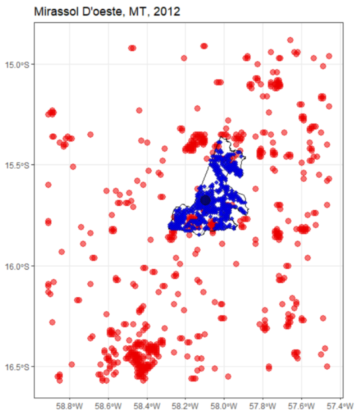

2013

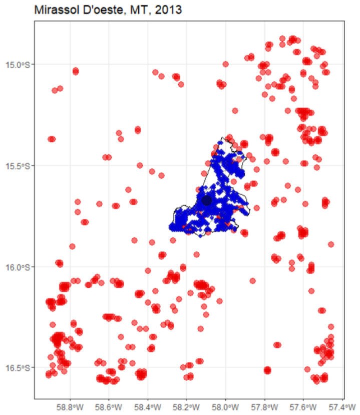

2014

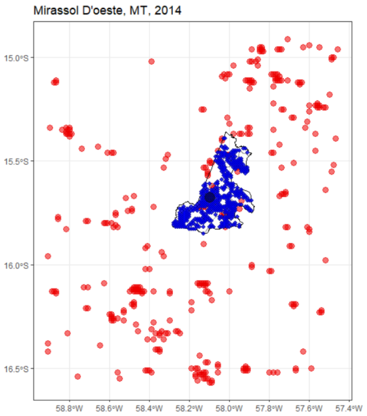

2015

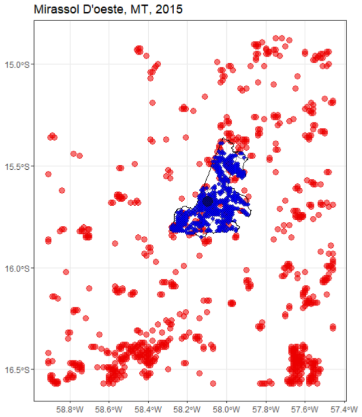

2016

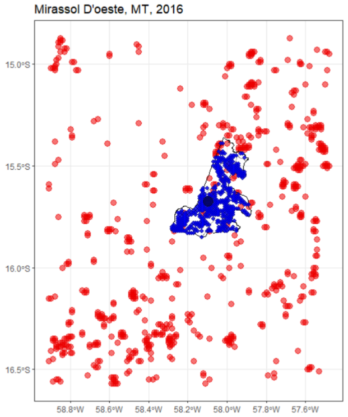

2017

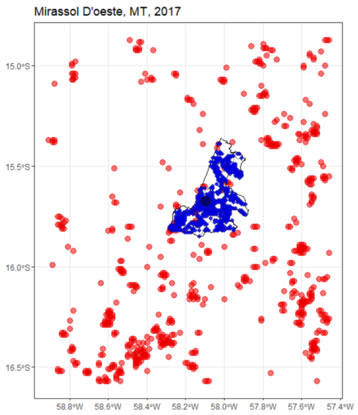

2018

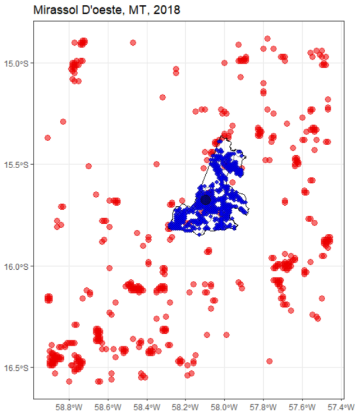

2019

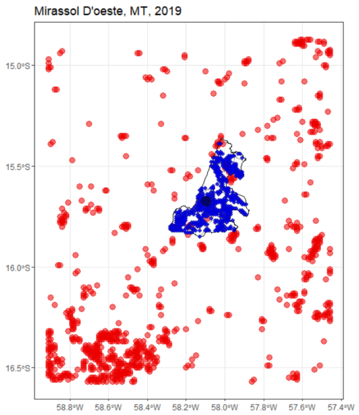

Year	Fire Outbreaks
2010	224
2011	93
2012	332
2013	159
2014	112
2015	292
2016	141
2017	193
2018	295
2019	423

Wildfire Overview

Fig. S14 - Annual evolution of fire outbreaks in the city of Nossa Senhora do Livramento - Mato Grosso.

2010

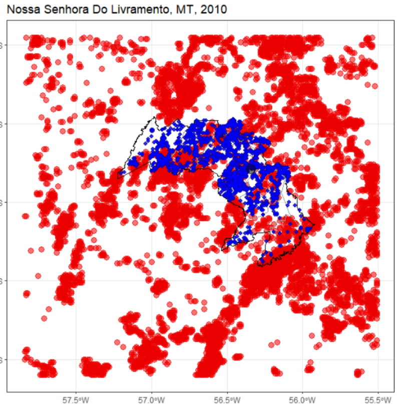

2011

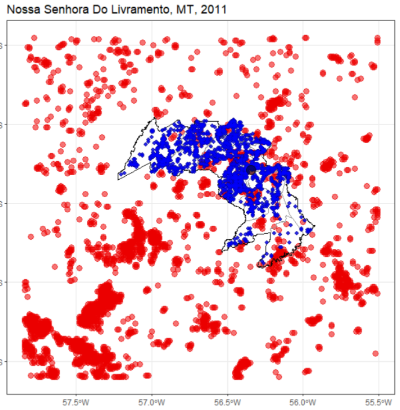

2012

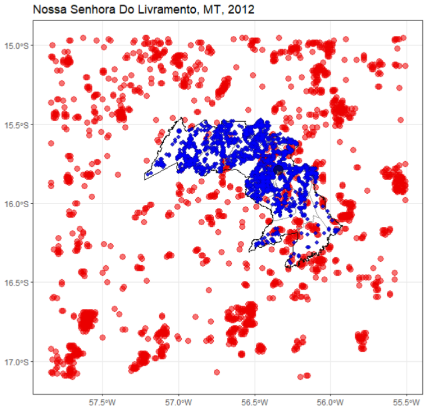

2013

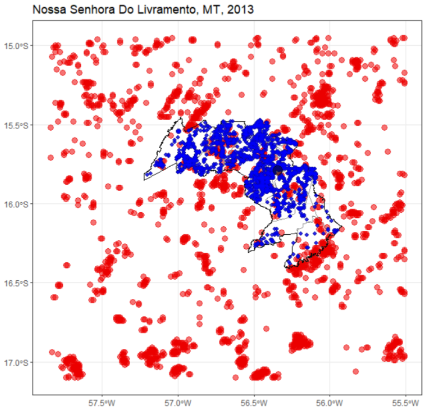

2014

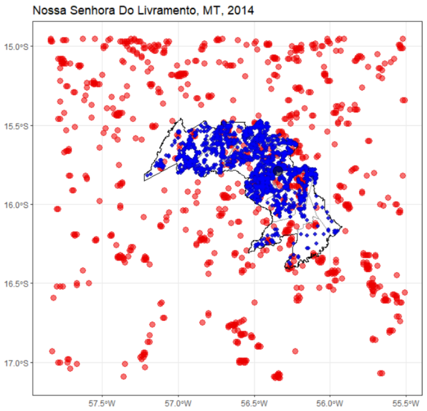

2015

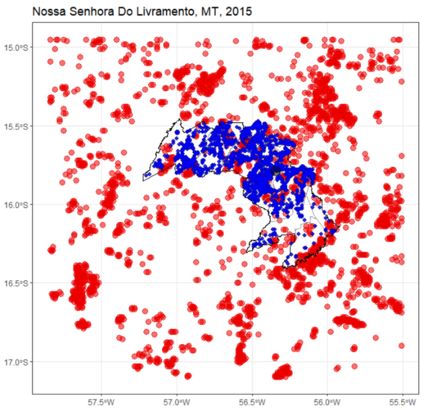

2016

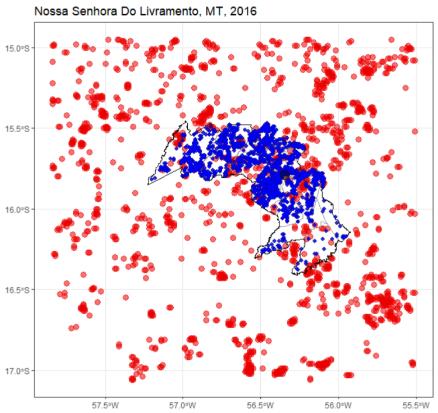

2017

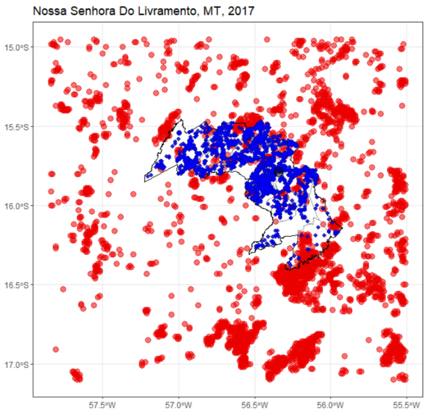

2018

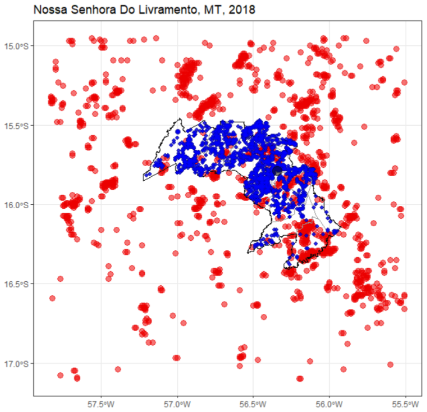

2019

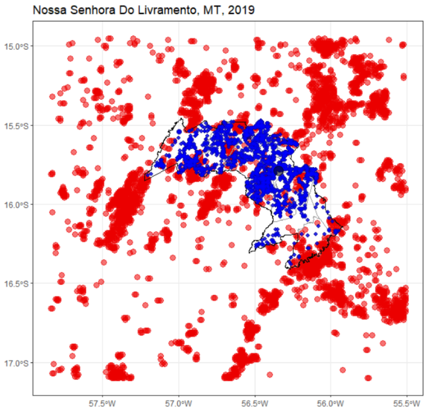

Year	Fire Outbreaks
2010	1383
2011	375
2012	823
2013	1070
2014	683
2015	1937
2016	1313
2017	2147
2018	2209
2019	3883

Wildfire Overview

Fig. S15 - Annual evolution of fire outbreaks in the city of Poconé - Mato Grosso.

2010

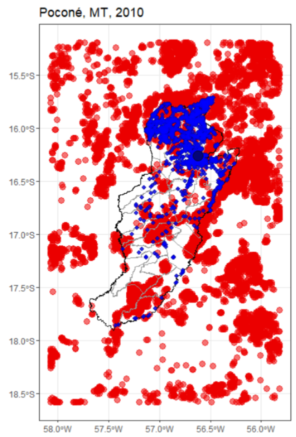

2011

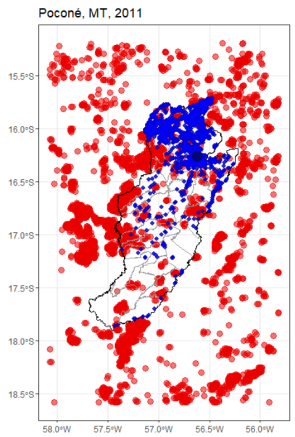

2012

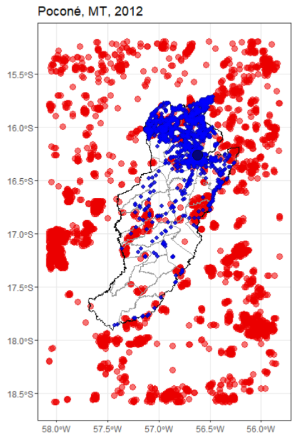

2013

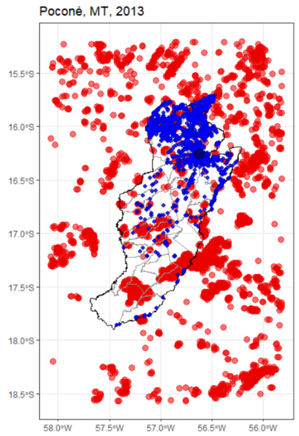

2014

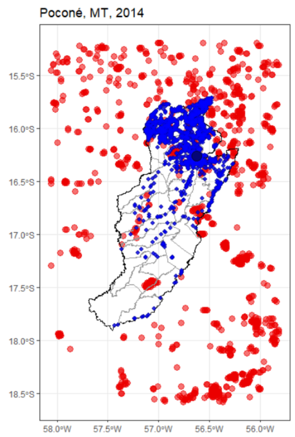

2015

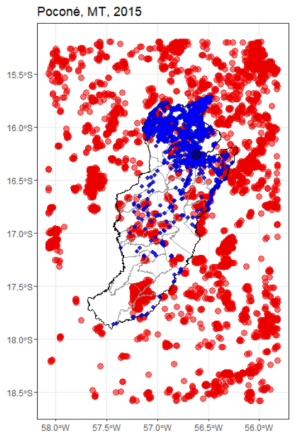

2016

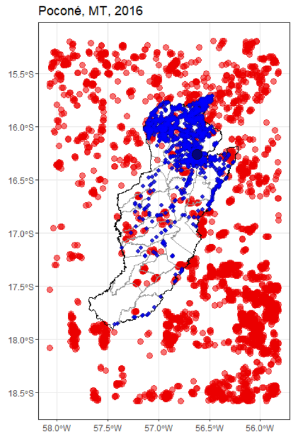

2017

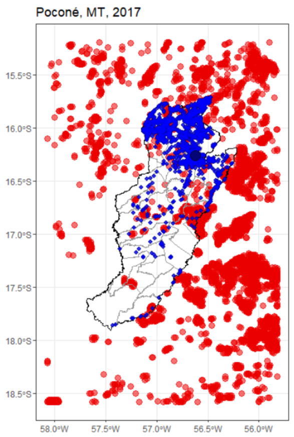

2018

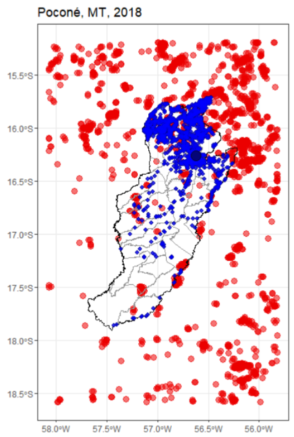

2019

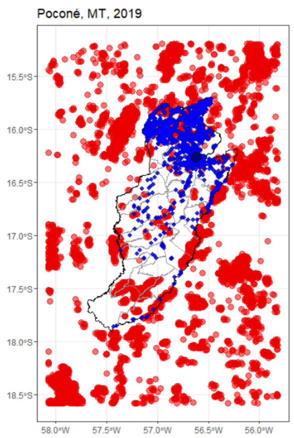

Year	Fire Outbreaks
2010	6426
2011	2864
2012	3521
2013	10197
2014	1189
2015	5938
2016	1918
2017	4635
2018	1764
2019	15594

Wildfire Overview

Fig. S16 - Annual evolution of fire outbreaks in the city of Porto Esperidião - Mato Grosso.

2010

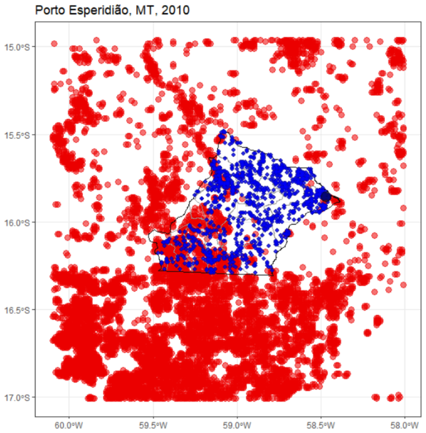

2011

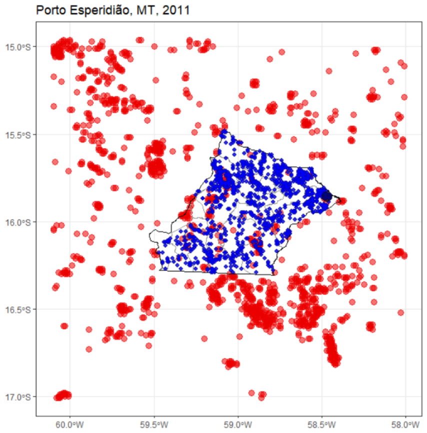

2012

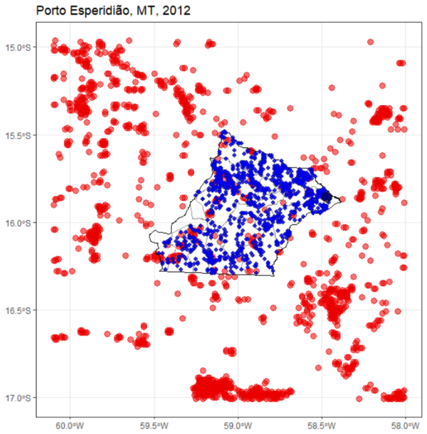

2013

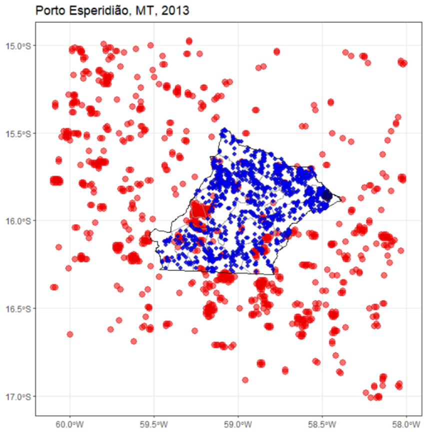

2014

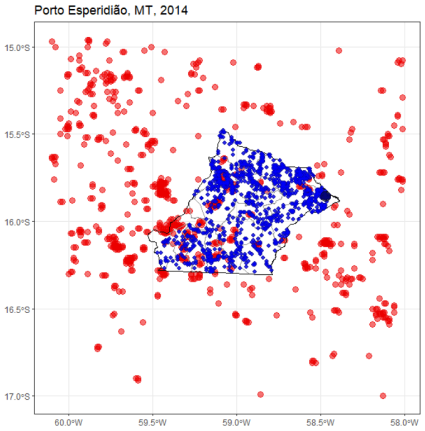

2015

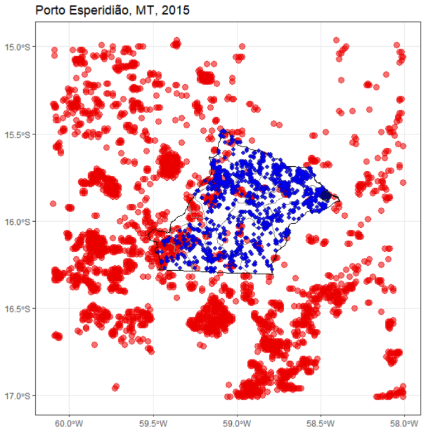

2016

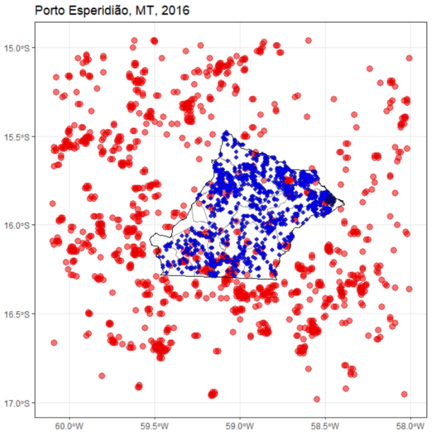

2017

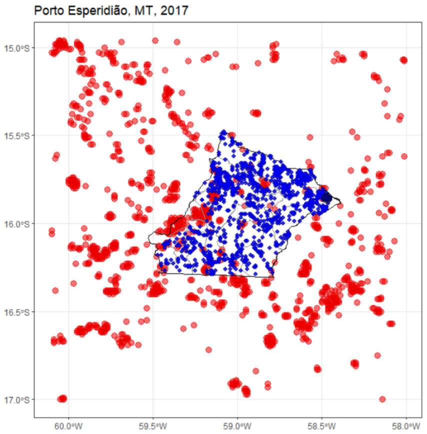

2018

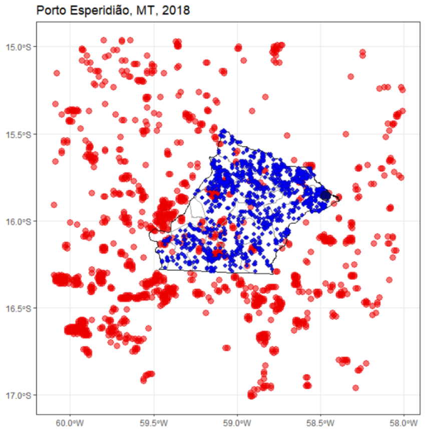

2019

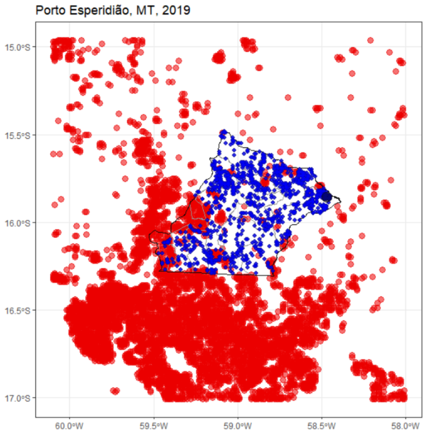

Year	Fire Outbreaks
2010	1409
2011	288
2012	856
2013	1129
2014	1046
2015	1994
2016	1022
2017	1904
2018	1692
2019	6787

Wildfire Overview

Fig. S17 - Annual evolution of fire outbreaks in the city of Porto Murtinho - Mato Grosso do Sul.

2010

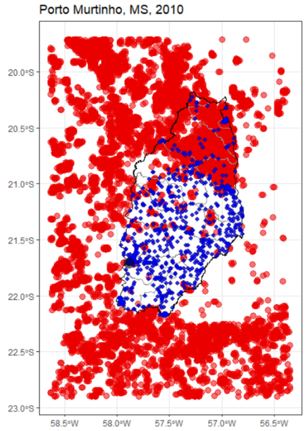

2011

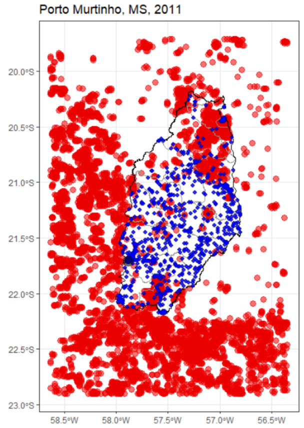

2012

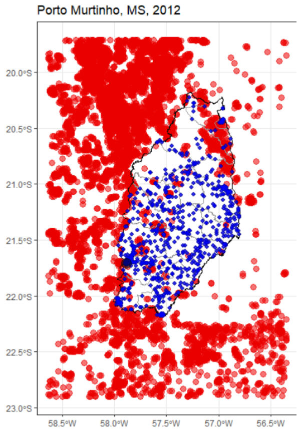

2013

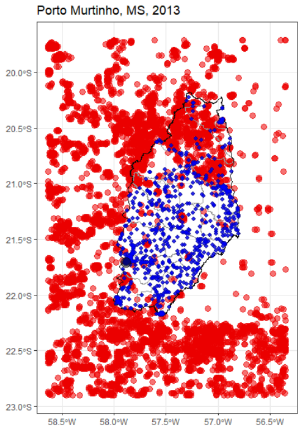

2014

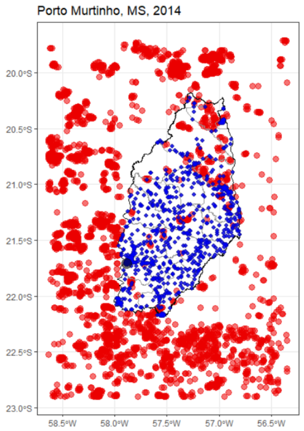

2015

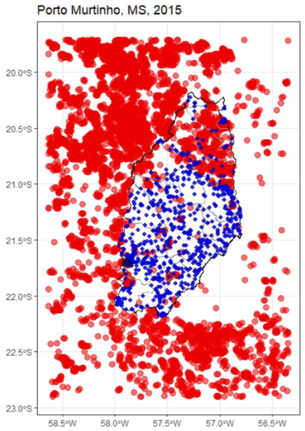

2016

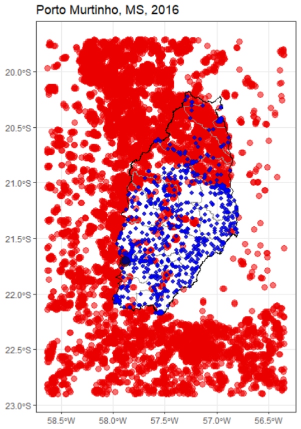

2017

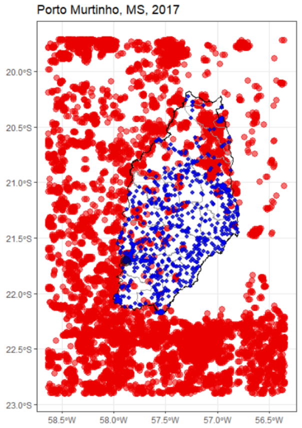

2018

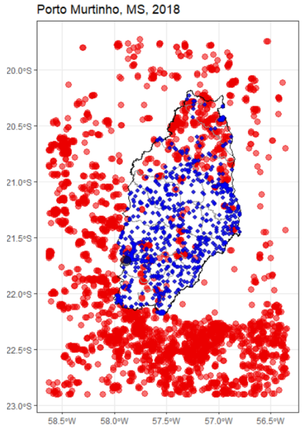

2019

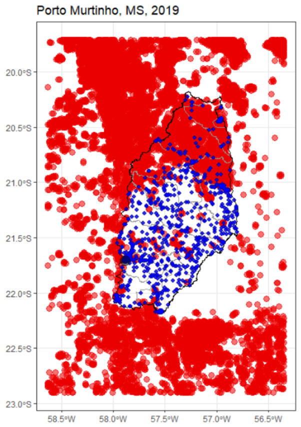

Year	Fire Outbreaks
2010	2961
2011	2139
2012	2953
2013	5764
2014	1491
2015	4877
2016	11211
2017	5475
2018	2762
2019	30399

Wildfire Overview

Fig. S18 - Annual evolution of fire outbreaks in the city of Rio Verde de Mato Grosso - Mato Grosso do Sul.

2010

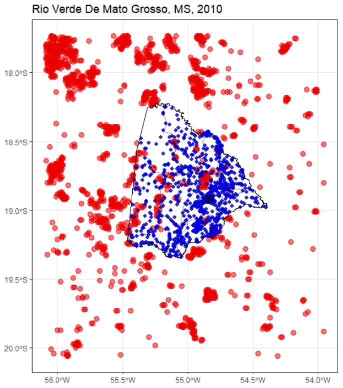

2011

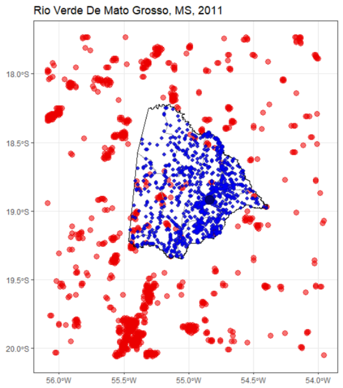

2012

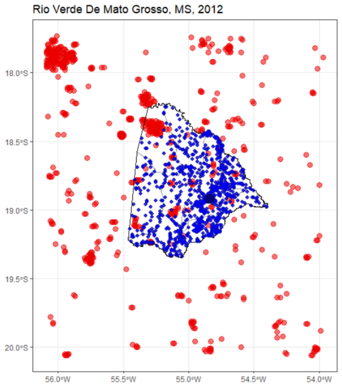

2013

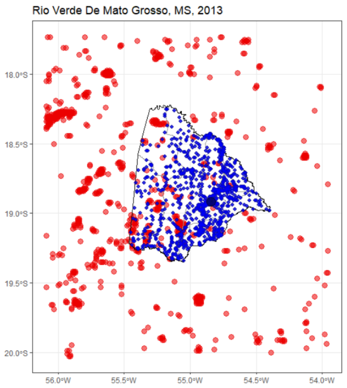

2014

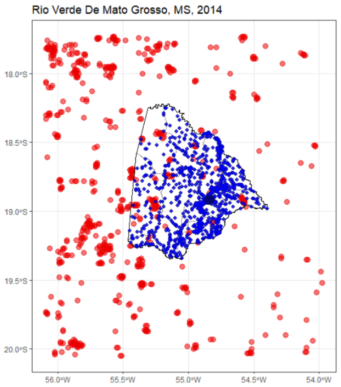

2015

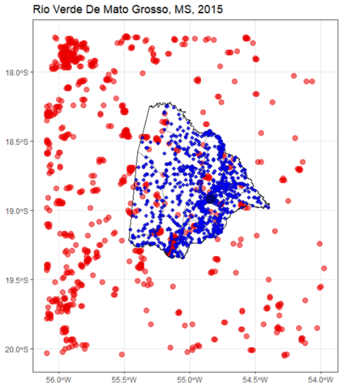

2016

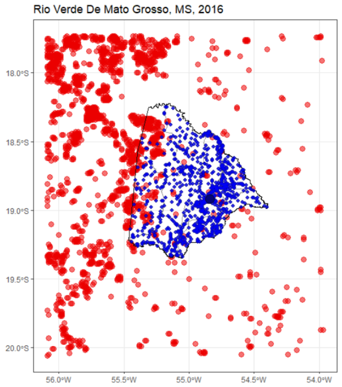

2017

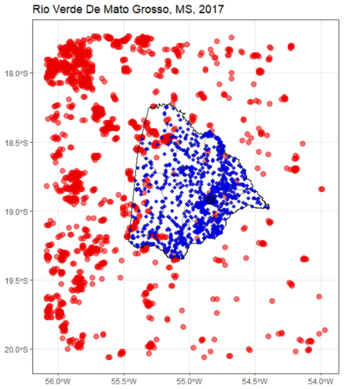

2018

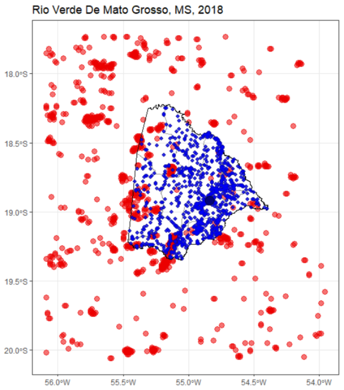

2019

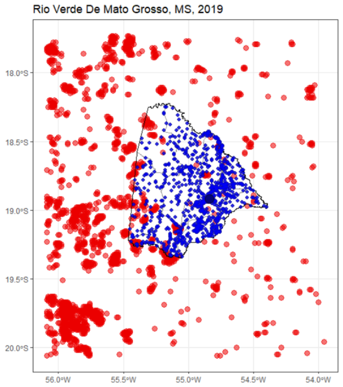

Year	Fire Outbreaks
2010	529
2011	189
2012	1251
2013	1226
2014	627
2015	929
2016	1745
2017	1109
2018	1557
2019	2072

Wildfire Overview

Fig. S19 - Annual evolution of fire outbreaks in the city of Rondonópolis - Mato Grosso

2010

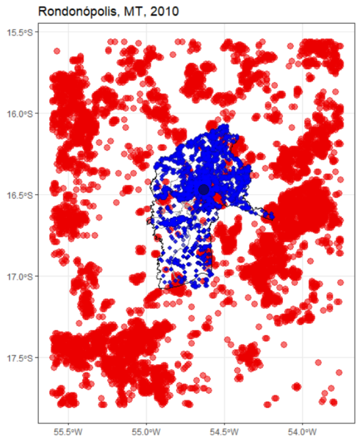

2011

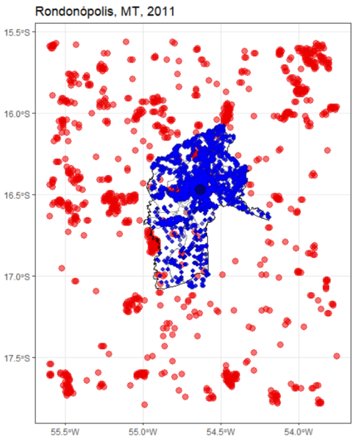

2012

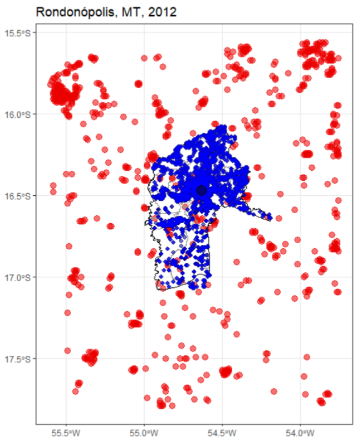

2013

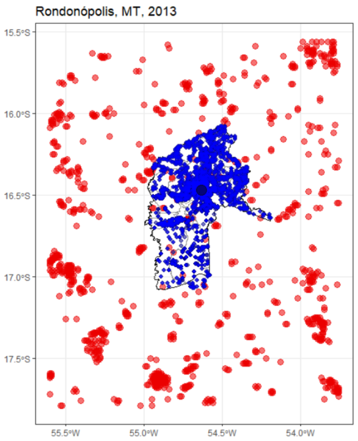

2014

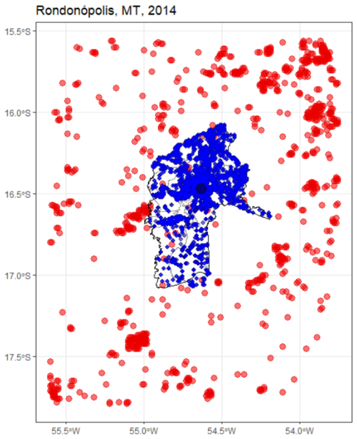

2015

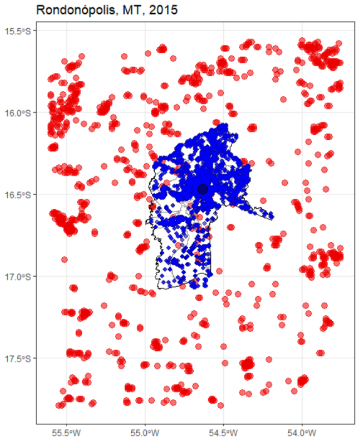

2016

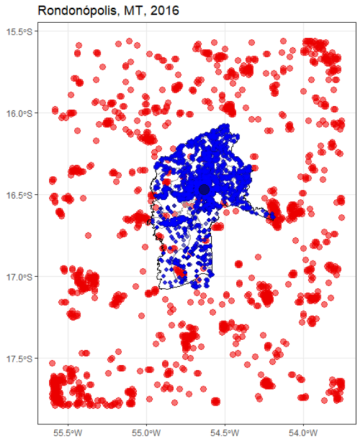

2017

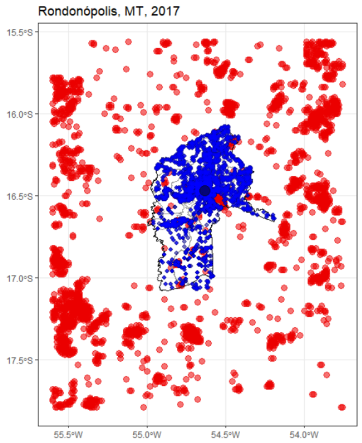

2018

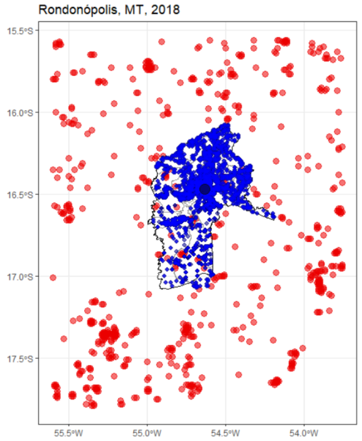

2019

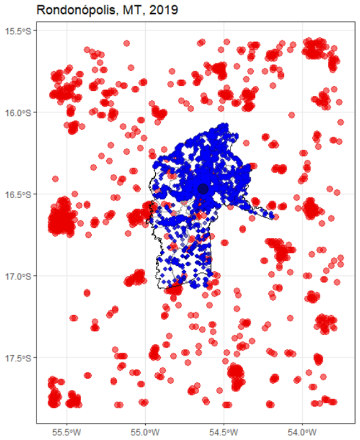

Year	Fire Outbreaks
2010	615
2011	134
2012	187
2013	240
2014	108
2015	183
2016	279
2017	649
2018	304
2019	764

Wildfire Overview

Fig. S20 - Annual evolution of fire outbreaks in the city of Santo Antônio do Leverger - Mato Grosso

2010

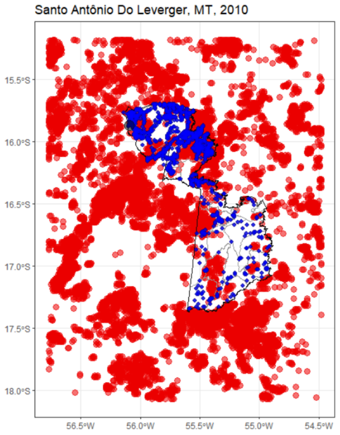

2011

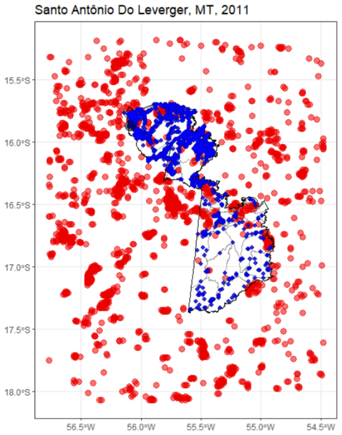

2012

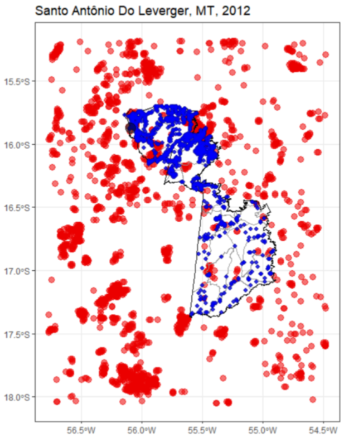

2013

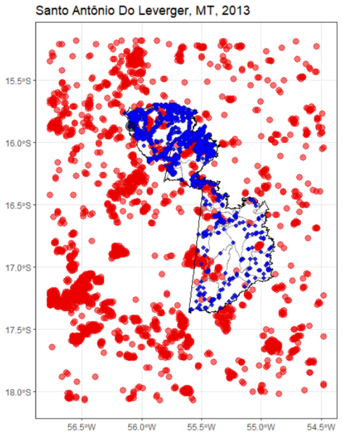

2014

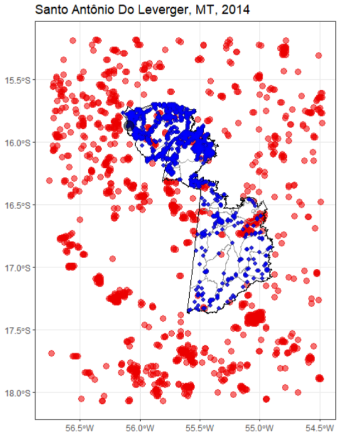

2015

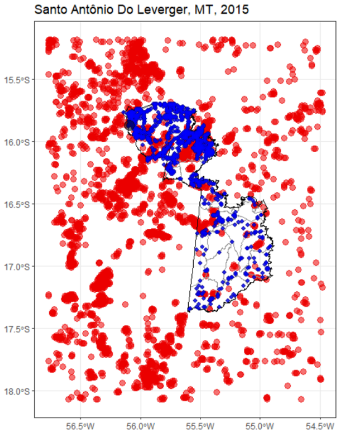

2016

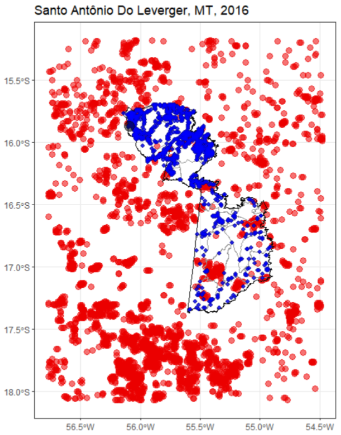

2017

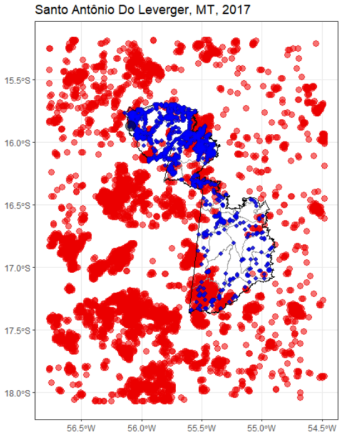

2018

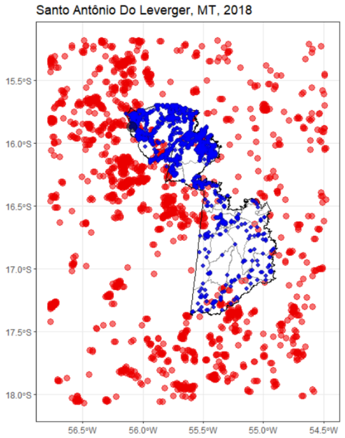

2019

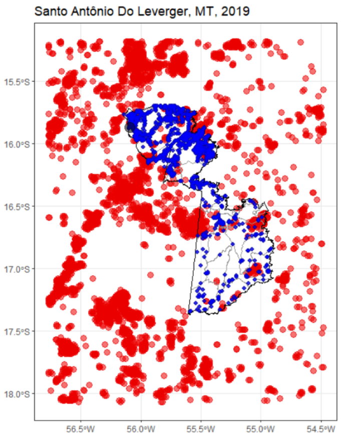

Year	Fire Outbreaks
2010	3221
2011	766
2012	2310
2013	1769
2014	762
2015	3048
2016	1778
2017	7303
2018	1559
2019	5294

Wildfire Overview

Fig. S21 - Annual evolution of fire outbreaks in the city of Sonora - Mato Grosso do Sul

2010

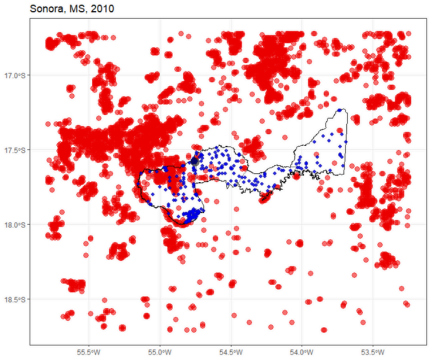

2011

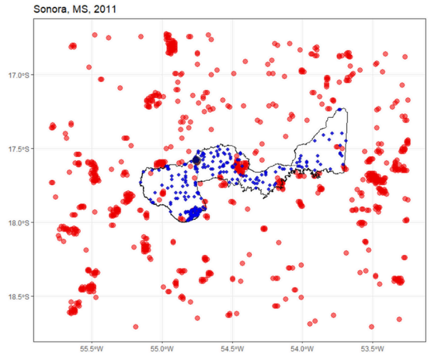

2012

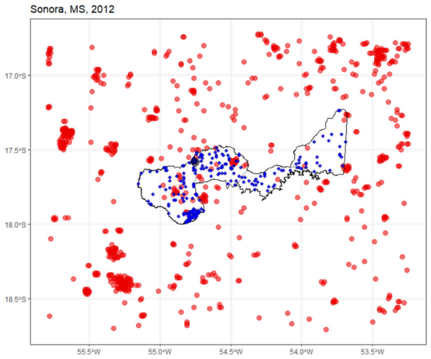

2013

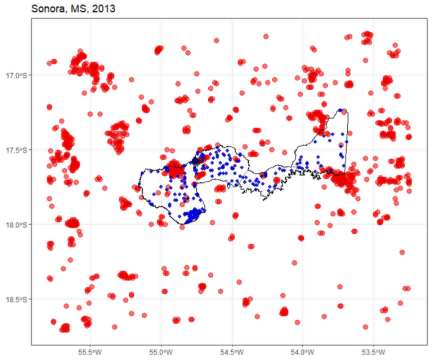

2014

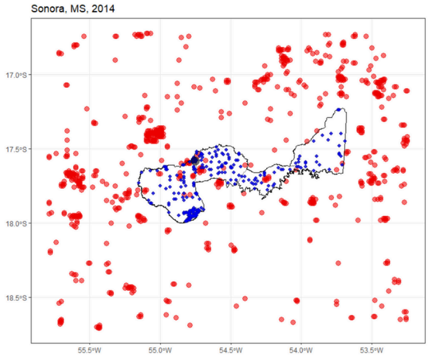

2015

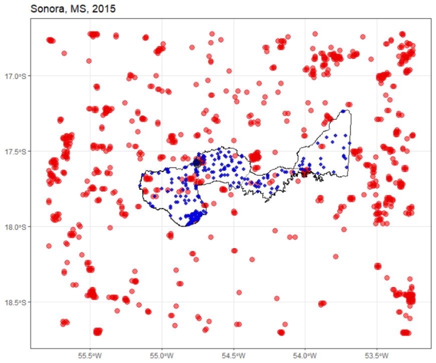

2016

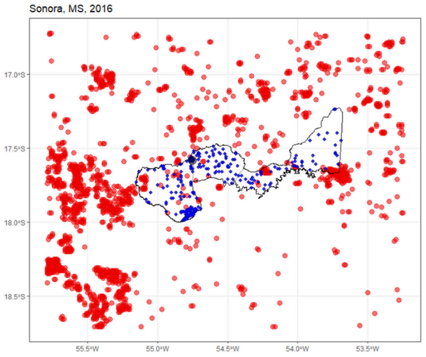

2017

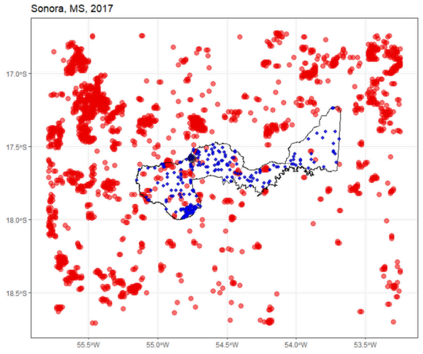

2018

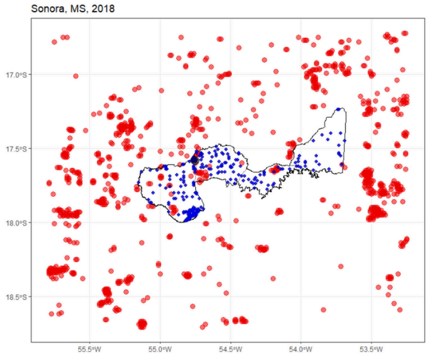

2019

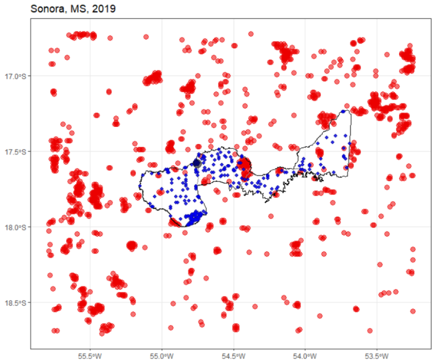

Year	Fire Outbreaks
2010	794
2011	290
2012	376
2013	948
2014	177
2015	389
2016	571
2017	388
2018	247
2019	1194

To address concerns regarding spatial overlap and clarity in Figure 6, we created annual raster maps aggregating both fire outbreak points and population zip code data into a fixed spatial grid (1 km²). Fire density (fires/km²) is represented by a red-to-yellow gradient, while population density is shown in blue shades. These heatmaps better illustrate the spatial distribution and yearly variation of fire outbreaks and population exposure across Corumbá. Additionally, time-series plots and a comparative difference map between 2011 and 2019 are provided to highlight temporal and spatial trends.

Fig. S22 – Annual total of fire outbreaks in the city of Corumbá, Mato Grosso do Sul (2010–2019)

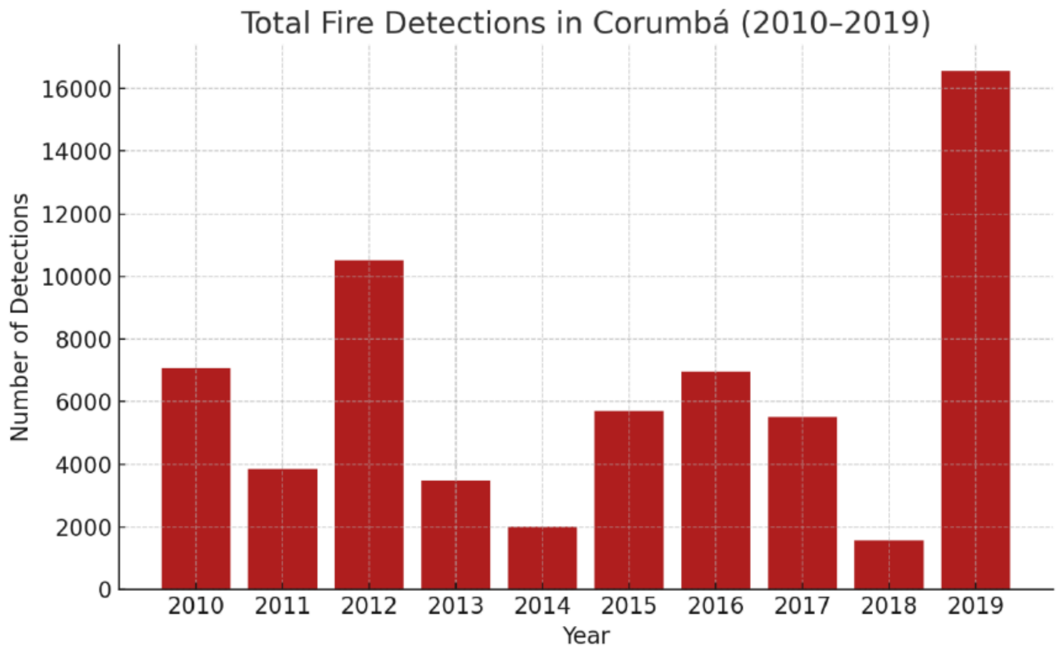

Temporal trend of fire detections in Corumbá based on MODIS satellite data. Each bar represents the total number of fire detections per year between 2010 and 2019.

Fig. S23 – Annual spatial distribution of fire outbreaks in Corumbá, Mato Grosso do Sul (2010–2019)

2010

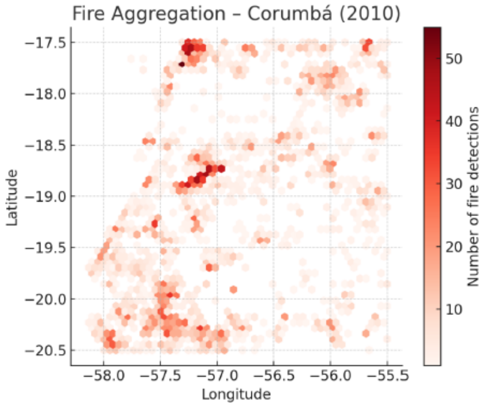

2011

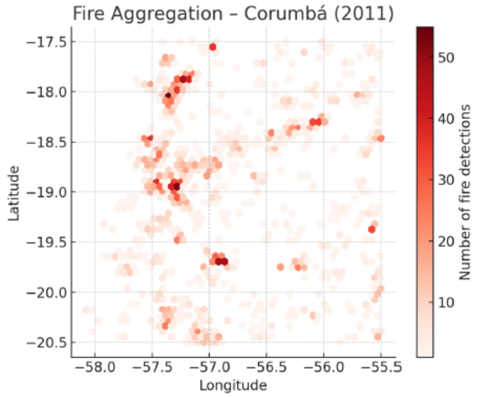

2012

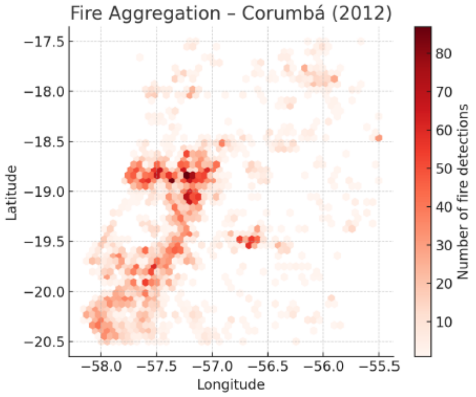

2013

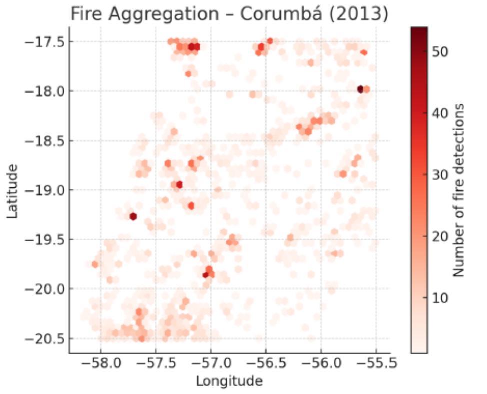

2014

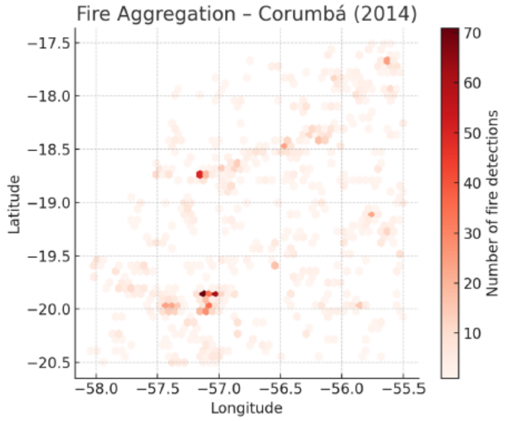

2015

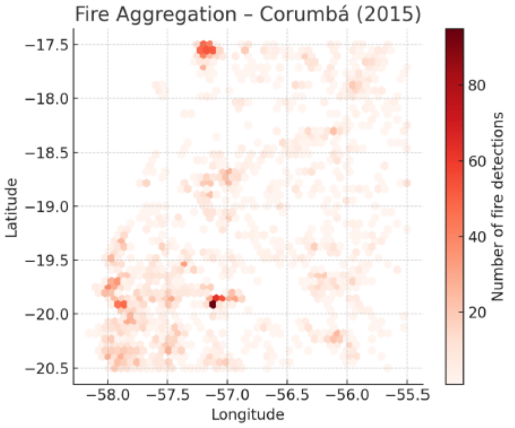

2016

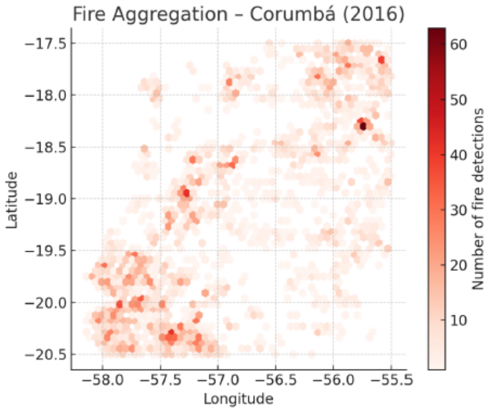

2017

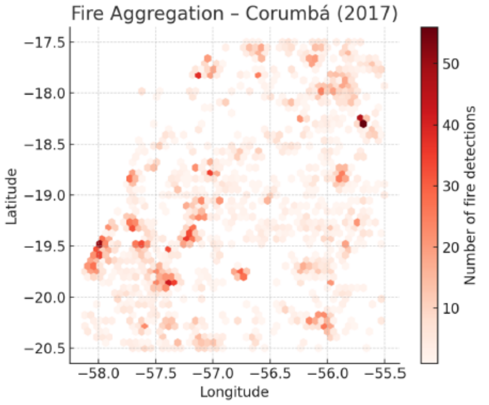

2018

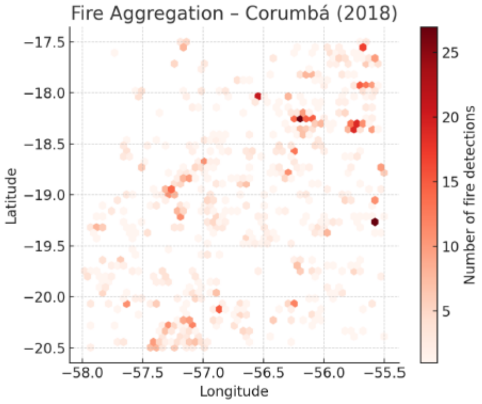

2019

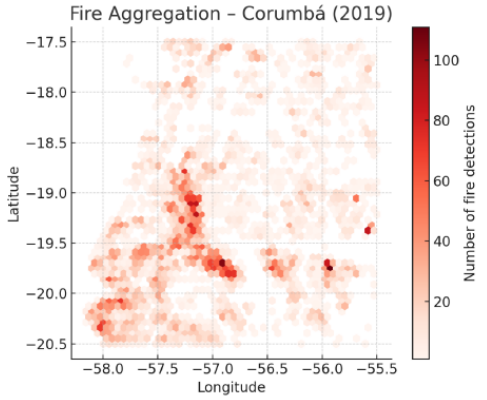

Supplement: Supplementary file 1 — Supplementary Material 1 [file 41598_2025_13257_MOESM1_ESM.pdf]
